# An Efficient CO_2_‐Upcycling Platform Based on Engineered *Halomonas* TD with Enhanced Acetate‐Utilizing Capacity via Adaptive Laboratory Evolution

**DOI:** 10.1002/advs.202513060

**Published:** 2025-10-24

**Authors:** Chi Wang, Ting‐Ting Chen, Yu‐Jiao Yang, Yu‐Xi Li, Yi‐Xin Chang, Yan‐Chun Xiao, Wen‐Tai Guo, Ye Zheng, Rui‐Zhe Deng, Yu‐Xiang Tian, Wei Situ, Hong‐Wei Shen, Yu Chen, Ya‐Bin Wang, Jie Xing, Hui Wang, Lin Xia, Yi‐Na Lin, Jian‐Wen Ye

**Affiliations:** ^1^ School of Biology and Biological Engineering South China University of Technology Guangzhou 510006 China; ^2^ School of Science China University of Geosciences Beijing 100083 China; ^3^ CAS Key Laboratory of Quantitative Engineering Biology Shenzhen Institute of Synthetic Biology Shenzhen Institutes of Advanced Technology Chinese Academy of Science Shenzhen 518055 China; ^4^ Food and Drug Department Liaoning Agricultural Vocational and Technical College Yingkou 115009 China; ^5^ Guangzhou Medpha Biotechnology Co., Ltd. Guangzhou 510006 China; ^6^ Guangdong Hefeng Biotechnology Co., Ltd. Zhanjiang 524300 China; ^7^ China Petroleum Engineering and Construction Corporation Beijing 100120 China; ^8^ Powered Carbon Biotechnology Co., Ltd Shenzhen 518055 China; ^9^ Department of General Surgery (Colorectal Surgery) The Sixth Affiliated Hospital Sun Yat‐sen University Guangzhou 510655 China; ^10^ Guangdong Provincial Key Laboratory of Colorectal and Pelvic Floor Diseases The Sixth Affiliated Hospital Sun Yat‐sen University Guangzhou 510655 China; ^11^ Biomedical Innovation Center The Sixth Affiliated Hospital Sun Yat‐sen University Guangzhou 510655 China

**Keywords:** acetate utilization, adaptive laboratory evolution, CO_2_‐derived electrolytes, *Halomonas*, metabolic engineering

## Abstract

Biohybrid conversion of carbon dioxide (CO_2_) into value‐added bioproducts via engineered microbes using CO_2_‐derived electrolytes (CDE) addresses global CO_2_ emissions, but most recombinants have poor saline CDE tolerance and low carbon conversion rate (CCR). Herein, *Halomonas* TD (salt‐resistant) was adaptively evolved into TD80, which efficiently uses acetate; its *aceE* gene mutation (encoding pyruvate dehydrogenase) drives acetate utilization. Subsequently, different biosynthesis pathways in TD80 enabled high yields of poly‐3‐hydroxybutyrate (PHB), poly‐3‐hydroxybutyrate‐*co*‐4‐hydroxybutyrate (P34HB), 3‐hydroxybutyrate (3HB), violacein, ectoine, 1,3‐diaminopropane (1,3‐DAP) and superoxide dismutase (SOD), respectively. Moreover, 26.0 g L^−1^ ectoine and 29.6 g L^−1^ PHB can be achieved by recombinant TD80 strains during fed‐batch studies. Finally, a non‐canonical pathway was designed to recycle the excess malonyl‐CoA into PHB. The resultant PHB content in fed‐batch study was increased from 60 wt% to 80 wt%. Moreover, co‐producing ectoine and PHB could further boost the CCR of CDE‐to‐product up to 53.7 mol%, which exemplified promising potential for biohybrid CO_2_ upcycling involved in carbon capture and utilization system. Furthermore, TD80 was engineered to grow on formate only aiming to achieve the full use of CDE. The establishment of technology and economy assessment (TEA) confirmed the *Halomonas*‐based platform’s efficiency and economic viability for carbon footprint reduction.

## Graphical abstract

An electrochemical system is designed to convert CO_2_ into CO_2_‐derived electrolytes (CDE), mainly containing acetate and formate. The underlying mechanism of acetate metabolism in *Halomonas* TD80 is explored via ALE. Engineered TD80 produced high‐yield diversified products from CDE. Furthermore, TD80 is engineered to utilize formate for PHB synthesis by the rGly pathway, demonstrating strong potential for full utilization of CDE in the future.

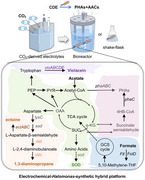

## 1 Introduction

The increasingly heavy emission of carbon dioxide (CO_2_) has caused significant crises such as climate change, which affects global economic and environmental sustainability.^[^ ^1^, ^2^, ^3^ ^]^ Although an extraordinary diversity of chemicals have been synthesized from fossil resources based on the advancement of chemical engineering over the past two centuries,^[^ ^4^ ^]^ the establishment of global carbon cycle dependent on the biological conversion from inorganic carbon toward usable biomass is becoming a key strategy to achieve a sustainable (bio) economy in the coming future, where the greenhouse gas (CO_2_) is one of the most important carbon source emitted from petrol‐based industries.^[^ ^5^ ^]^ Therefore, many efforts have been made to achieve upcycling of CO_2_ toward value‐added products powered by electrolysis,^[^ ^6^, ^7^ ^]^ chem‐biological system,^[^ ^8^, ^9^ ^]^ electrochemical–biological hybrid system,^[^ ^10^, ^11^, ^12^, ^13^, ^14^, ^15^, ^16^ ^]^ etc., which provide attractive solutions for carbon reduction to achieve a circular carbon economy.^[^ ^17^, ^18^, ^19^, ^20^ ^]^ Converting CO_2_ into chemicals, including methanol, formic acid, etc., based on electrolysis has been applied successfully in lab‐ and industry‐scale production levels.^[^ ^21^, ^22^ ^]^ Besides, chem‐biological synthesis of biomacromolecules, such as starch and poly‐3‐hydroxybutyrate (PHB), from CO_2_ directly via enzyme‐based catalysis has been developed in previous studies, demonstrating promising success in carbon‐chain lengthening from a monocarbon compound.^[^ ^8^, ^9^ ^]^ However, challenges still remain to further expand the production line that allows diverse value‐added compounds synthesis.^[^ ^15^, ^23^, ^24^ ^]^ Moreover, direct utilization of CO_2_, or CO_2_‐derived syngas, by recombinant microbes is an alternative strategy to produce bioproducts, including ethanol^[^ ^25^ ^]^ and sugar,^[^ ^26^ ^]^ but low productivity and explosion‐proof issues are potential limits for large‐scale industrial uses. Additionally, the electrochemical–biological hybrid system has been recently developed aiming to produce desired products of diversity, like isobutanol,^[^ ^11^ ^]^ protein,^[^ ^12^ ^]^ sugars,^[^ ^10^, ^13^ ^]^ Polyhydroxyalkanoates (PHA),^[^ ^15^ ^]^ etc., by engineered microbial cells, including recombinant *E.coli*,^[^ ^11^ ^]^ *Pichia pastoris*,^[^ ^12^ ^]^ *S. cerevisiae*,^[^ ^10^, ^13^ ^]^ *Pseudomonas putida* ^[^ ^15^ ^]^ and so on, using CDE as the sole carbon source generated from electrochemical CO_2_ reduction. However, for most recombinant strains, poor tolerance to CDE, which is a high‐salt solution mainly containing acetate, and low carbon‐to‐production conversion rate, still hinder their further industrial uses.^[^ ^10^ ^]^ To address these challenges, *Halomonas* TD, a well‐studied PHA‐producing strain with natural strong resistance to saline conditions containing high‐concentration sodium chloride,^[^ ^27^ ^]^ sodium acetate,^[^ ^28^ ^]^ etc., can be an ideal chassis for metabolic engineering with the aim of using CDE solution as the only feedstock.

In the past decade, *Halomonas* TD has been engineered to produce low‐cost PHA productions, such as PHB, poly(3‐hydroxybutyrate‐*co*‐4‐hydroxybutyrate) (P34HB),^[^ ^29^ ^]^ poly(3‐hydroxybutyrate‐*co*‐3‐hydroxypropionate) (PHBP),^[^ ^30^ ^]^ poly(3‐hydroxybutyrate‐*co*‐3‐hydroxyhexanoate)(PHBHHx)^[^ ^31^ ^],^ and high‐value compounds like ectoine^[^ ^32^, ^33^ ^]^ and threonine.^[^ ^34^ ^]^ This has been achieved using glucose as a carbon source under open and unsterile conditions from lab‐ to industrial pilot‐scale (5000‐L) fermentors.^[^ ^32^ ^]^ Nevertheless, engineering *Halomonas* TD to convert CDE into high‐value‐added compounds and biodegradable plastics (PHAs) could not only present a win‐win strategy for reducing CO_2_ emissions and plastic pollution,^[^ ^35^ ^]^ but also further broaden the application scopes and industrial value of the next‐generation industrial biotechnology (NGIB) platform based on *Halomonas*.^[^ ^36^, ^37^ ^]^ In previous studies, *Halomonas* TD has already been found able to survive under high concentrations of sodium acetate reaching up to 75 g L^−1[^ ^38^ ^]^ which is highly similar to the well‐known acetic acid bacteria like *Acetobacter aceti* and *Gluconobacter suboxydans*.^[^ ^39^ ^]^ Although *Halomonas* TD exhibits a relatively high tolerance to acetate, the cell growth was still restricted, with the OD_600_ value decreased by more than 50%. Adaptive laboratory evolution (ALE) was thus utilized to enhance cell growth and PHB accumulation from acetate during 150‐h fed‐batch fermentation, resulting in a 1.5‐fold improvement of PHB productivity (0.332 g L^−1^ h^−1^),^[^ ^28^ ^]^ which is still dramatically lower than that using glucose as a carbon source. Therefore, engineering *Halomonas* TD to be a chassis for an electrochemical‐biological hybrid system capable of synthesizing diversified products from acetate (or CDE) still remains unsolved despite its acetate‐resistant nature.

Herein, this study is aimed at developing an electrochemical–biological hybrid system based on *Halomonas* for effective CDE utilization of high‐performing CO_2_‐upcycling capacity. Bioproducts, including different PHA biopolymers (such as PHB and P34HB) and value‐added compounds (like violacein, ectoine, 1,3‐diaminopropane (1,3‐DAP), 3HB, and SOD) were successfully produced by metabolically engineered TD80, an ALE‐mutant chassis of strong CDE tolerance and utilization capability derived from *Halomonas* TD, grown on CDE as the sole carbon source. This study presents an efficient and economically viable strategy for biohybrid utilization of CO_2_.

## 2 Results and Discussion

### 2.1 Adaptive Laboratory Evolution of *Halomonas* TD for Enhanced Acetate Utilization

**Figure 1:**
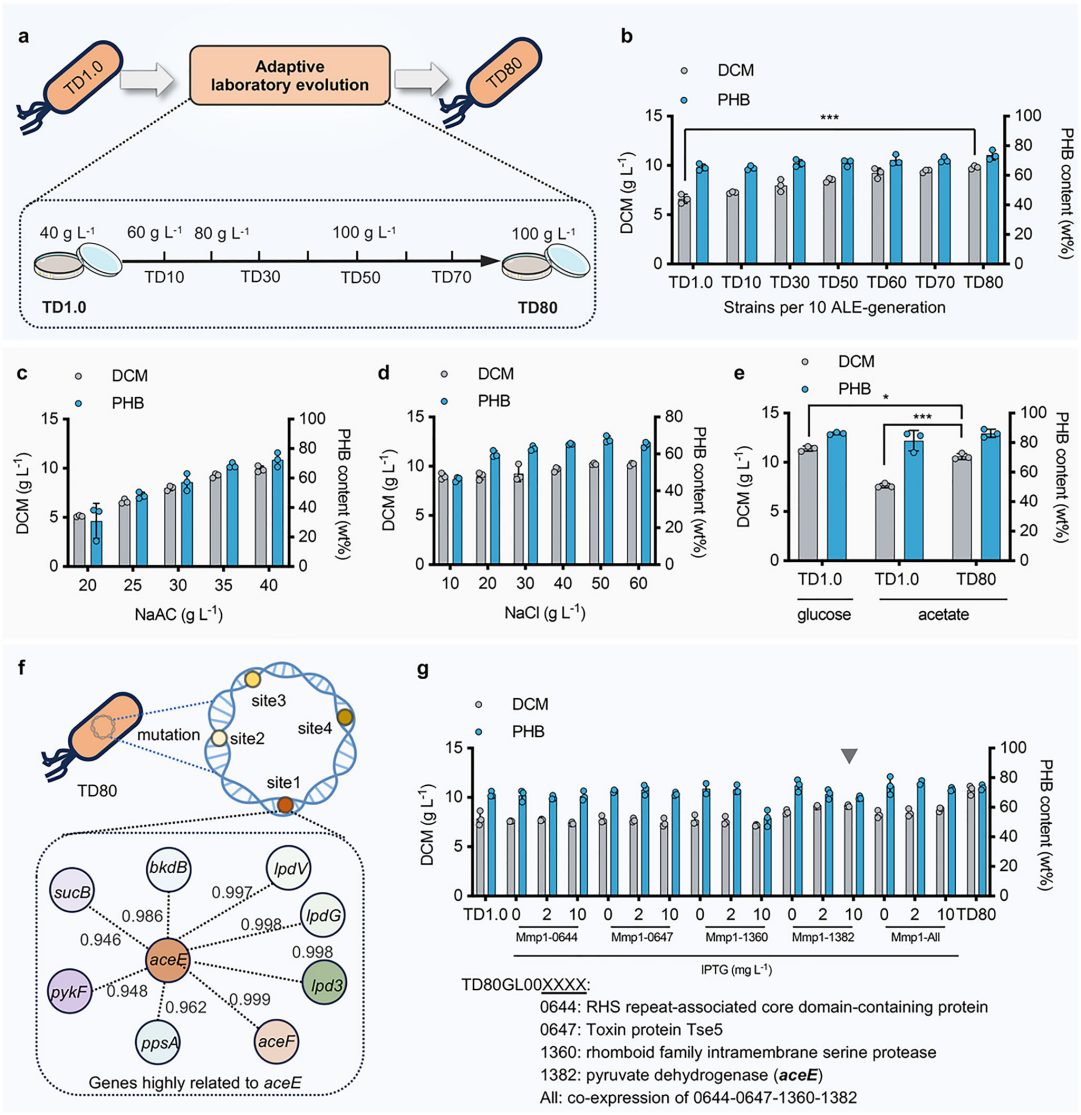
Adaptive laboratory evolution of *Halomonas* TD1.0 for enhanced acetate utilization. a) Schematic design of adaptive laboratory evolution (ALE) for the start host *Halomonas* TD1.0 grown on 50MM agar plates supplemented with increased acetate concentration (from 40 to 100 g L^−1^). The most dominant mutant TD80 with significantly improved acetate‐utilizing capability was screened out after 80 generations of passaging every 48 h. b) Shake flask studies of dry cell mass (DCM) and PHB content by evolved *Halomonas* TD1.0, namely TD10, TD30, TD50, TD60, TD70, and TD80, throughout the ALE process. The start host TD1.0 grown in 50MM medium supplemented with 40 g L^−1^ acetate was used as a negative control. c, d) Media optimization for improved cell growth and PHB accumulation by TD80 conducted in 500‐mL shake flasks. e) Shake flask studies of cell growth and PHB accumulation by TD80 and TD1.0 grown on acetate, in contrast to TD1.0 grown on glucose. f) Comparative genomic analysis of TD80 uncovered the dominant ALE‐mutated gene (*aceE*) encoding pyruvate dehydrogenase, resulting in enhanced acetate utilization. The major interactive network of *aceE* involved in acetyl transferation was identified by STRING. *aceF*, pyruvate dehydrogenase e2 component; *lpd3*, dihydrolipoamide dehydrogenase; *lpdG*, dihydrolipoamide dehydrogenase; *lpdV*, dihydrolipoamide dehydrogenase; *bkdB*, branched‐chain alpha‐keto acid decarboxylase; *ppsA*, phosphoenolpyruvate synthase; *pykF*, pyruvate kinase F; *sucB*, dihydrolipoyllysine‐residue succinyltransferase. g) Four major ALE‐mutated genes, including *aceE*, were overexpressed in the start host TD1.0 to study their contribution to acetate metabolism. TD1.0 and TD80 grown on glucose and acetate, respectively, were used as positive control groups. *n* = 3, ^*^ *p*<0.05; ^***^ *p*<0.001.

First, ALE was employed to improve the acetate tolerance and utilization of *Halomonas* TD. Before the execution of ALE, the start host, *Halomonas* TD1.0, grown on 50 MMA agar plates supplemented with 40–120 g L^−1^ acetate, respectively, was first used to assess its acetate‐tolerant capability. Compared with the fast growth (24‐h) of TD1.0 strain on a 50MMG agar plate supplemented with 20 g L^−1^ glucose, survival colonies could only be obtained after 48‐h incubation when the acetate concentration was no greater than 80 g L^−1^, indicating an upper growth limit of acetate for TD1.0. Therefore, TD1.0 strain was first grown on a 50MMA agar plate containing 40 g L^−1^ acetate to initiate the ALE experiment with acetate concentration increased by 20 g L^−1^ when the evolved cell colonies reached the same size as the start host grown on a 50MMG agar plate (control group) after 24‐h incubation (**Figure** **1**a). ALE of TD1.0 strain was terminated at the 80^th^ generation, namely TD80, since the colony size after 30‐generation passaging growth in 100 g L^−1^ acetate was still slightly smaller than that of the control group. Throughout the ALE process, the evolved mutants at different representative ALE‐generations, namely TD10, TD30, TD50, TD60, TD70, and TD80, were selected for shake flask study grown on 40 g L^−1^ acetate only to access the cell growth and PHB accumulation. Among the six mutants, TD80 exhibited the best performance in dry cell mass (DCM), PHB content and CCR of acetate‐to‐PHB, reaching 9.8, 7.2 g L^−1^ and 34.5 mol%, respectively, which were increased by 48.2%, 67.0%, and 30.7% compared to the start host TD1.0 (Figure 1b; Figure S1a, Supporting Information). Subsequently, liquid medium‐based ALE using TD80 as the start host was carried out to obtain the TD120 mutant after 40‐generation passaging growth in a 500‐mL shake flask containing 50 mL 50MMA medium supplemented with up to 120 g L^−1^ acetate (Figure S2a, Supporting Information). However, the DCM and PHB accumulation by TD120 (10.58 g L^−1^, 83.4%) showed slightly decrease in contrast to TD80 (10.59 g L^−1^, 86.3%) (Figure S2b, Supporting Information). Therefore, TD80 was selected as an acetate‐utilizing chassis for further studies. Medium optimization was then carried out to determine that 35 g L^−1^ acetate and 50 g L^−1^ NaCl were the optimal conditions (DCM, PHB titer) for TD80 growth, with the highest CCR of around 35.5 mol% (Figures 1c,d; Figure S1b,c, Supporting Information). Importantly, TD80 grown under the optimized conditions exhibited similar performance in DCM and PHB accumulation compared to the starting host (TD1.0) grown on 30 g L^−1^ glucose (Figure 1e). The success of sufficient acetate utilization by TD80 presents great potential for direct CDE utilization in further study.

After whole genome sequencing of TD80, 17 ALE‐mutated coding sequences (CDS) with over 80 amino acid mutation sites were found based on comparative genomic analysis against the TD1.0 strain (Table S1, Supporting Information). Of them, only 4 mutated genes, including genes TD80GL00: 0664/0647/1360/1382, were identified as the ones that probably contributed to the improved acetate tolerance and utilization of TD80 (Figures 1f,g, highlighted in red in Table S1, Supporting Information). These 4 genes were thus overexpressed under the control of IPTG‐induced promoter P_MmP1_,^[^ ^40^ ^]^ individually and simultaneously, in TD1.0 for function verification. Notably, only the mutated *aceE* gene (TD01GL001382) encoding pyruvate dehydrogenase exhibited obvious improvement in DCM (16.0%) and PHB titer (12.2%) compared with TD1.0 (Figure 1g, highlighted with an inverse triangle). Furthermore, protein‐protein interaction analysis by STRING (online tool, https://version‐11‐5.string‐db.org) showed that AceE displays high correlations to the metabolic networks involved in pyruvate‐to‐acetyl‐CoA and α‐keto‐glutarate‐to‐succinate reactions (Figure 1f; Figure S3, Supporting Information). Therefore, a deeper insight into acetate metabolism was tentatively uncovered, providing a useful foundation for metabolic engineering of TD80 to achieve enhanced acetate utilization capability in further study. Moreover, in addition to acetate, the utilization capability of various short‐chain length fatty acids (SCFAs, C_1_–C_5_), which are common components in CDE^[^ ^41^ ^]^ or orther industrial waste (e.g., 1,3‐propanediol‐derived mother liquor from Guangdong Hengtan Technology Co., Ltd), was also assessed by TD80, exhibiting strong capability in SCFAs utilization for PHB synthesis (Figure S4, Supporting Information).

In summary, an acetate‐utilizing halophilic chassis, TD80, was obtained for developing an electrochemical–biological hybrid system for CO_2_‐upcycling purposes.

### 2.2 Engineering TD80 for PHAs and 3HB Synthesis from Acetate

**Figure 2:**
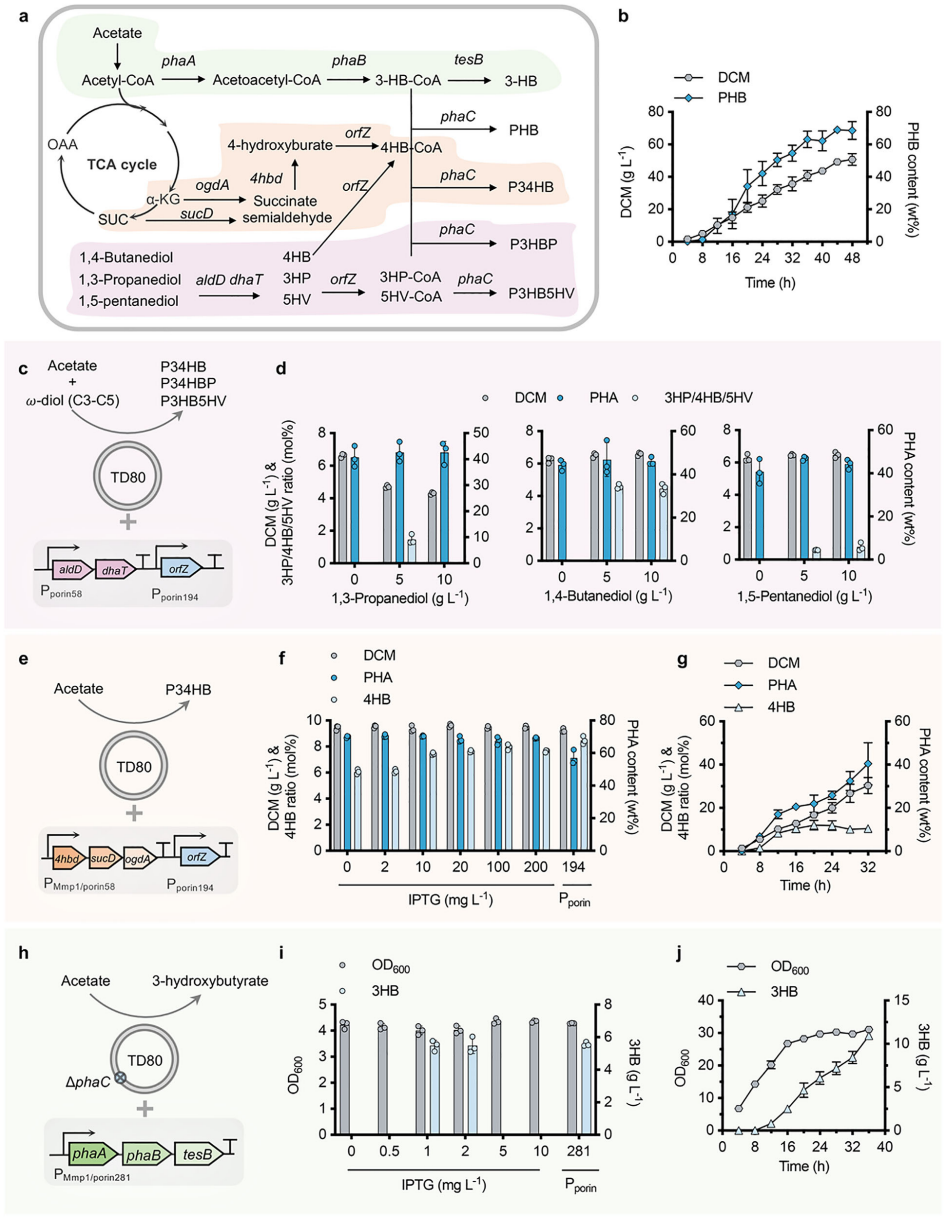
Engineering TD80 for effective production of PHA copolymers and 3HB from acetate. a) Metabolic pathway design for 3HB and different PHAs (PHB, P34HB, P3HBP, and P3HB5HV) synthesis by recombinant TD80. b) Fed‐batch study for PHB production by TD80 grown on acetate as a sole carbon source. c, d) DCM and PHA accumulation by recombinant TD80 harboring *aldD‐dhaT* and *orfZ* expression modules grown in 50MMA medium supplemented with different concentrations of *ω*‐diols (0, 5, and 10 g L^−1^ of 1,3‐propanediol, 1,4‐butanediol, and 1,5‐pentanediol). e) De novo synthesis of P34HB from acetate by recombinant TD80 harboring *4hbd‐sucD‐ogdA* and *orfZ* modules under the control of IPTG‐induced P_T7‐like_ promoter and P_porin194_ promoter, respectively. f) Expression tuning of the *4hbd‐sucD‐ogdA* module by adding different IPTG concentrations directs optimal constitutive promoter (P_porin58_) selection to achieve enhanced production of P34HB with a higher 4HB molar ratio. g) Fed‐batch study for P34HB production by recombinant TD80 harboring *4hbd‐sucD‐ogdA* and *orfZ* modules driven by P_porin58_ and P_porin194_, respectively. h) Schema for 3HB biosynthesis by recombinant TD80 from acetate. i) Expression tuning of the *phaA‐phaB‐tesB* module by IPTG‐induced P_T7‐like_ directs optimal constitutive P_porin_ promoter selection for enhanced 3HB synthesis. j) Fed‐batch study for 3HB production by recombinant TD80 harboring fine‐tuned *phaA‐phaB‐tesB* module. *phaA*, 3‐ketothiolase; *phaB*, acetoacetyl‐CoA reductase; *phaC*, PHA synthase; *sucD*, succinate semialdehyde dehydrogenase; *4hbD*, 4‐hydroxybutyrate dehydrogenase; ogdA, 2‐oxoglutarate decarboxylase; *orfZ*, CoA transferase; *tesB*, thioesterase; *dhaT*, 1,4‐butanediol reductase; *aldD*, aldehyde dehydrogenase. Fed‐batch studies in parts b, g, j were conducted in a 7‐L bioreactor. Error bars represent standard deviations, *n* = 3 (for fed‐batch studies, *n* = 2).

In previous studies, *Halomonas* TD has been engineered to be an industrial PHA‐producing chassis due to its outstanding PHB‐accumulating nature, aiming to store carbon source under extreme circumstances. High‐salt and high‐pH tolerance also confers strong contamination resistance capability of *Halomonas* TD and its derivatives, which enables an open and unsterile fermentation process of low cost. Nevertheless, engineering the synthesis of different PHA productions by TD80 from acetate by harnessing its salt‐resistance power not only can achieve a win‐win goal for reducing CO_2_ emissions and plastic pollution, but also is an effective strategy for energy‐rich carbon storage from CO_2_. Different synthesis pathways were thus designed to generate diversified PHA polymers consisting of 3‐hydroxybutyrate (3HB), an endogenously synthesized monomer for PHB accumulation, and other short‐chain‐length units, including 3‐hydroxypropionate (3HP), 4‐hydroxybutyrate (4HB), and 5‐hydroxyvalerate (5HV), respectively, from acetate with additional supplementation of structure‐related carbon sources whenever necessary (**Figure** **2**a).

First, fed‐batch studies (triplicate) of PHB by TD80 grown in a 7‐L bioreactor were assessed using acetate as a sole carbon source, which yielded 52.8 g L^−1^ DCM containing 71.8wt.% PHB with volumetric productivity reaching 0.8 g L^−1^ h^−1^ after 48 h cultivation (Figure 2b; Figure S5, Supporting Information). Based on the promising performance of PHB synthesis by TD80, two expression modules including *dhaT*‐*aldD* controlled by P_porin58_ promoter and *orfZ* gene controlled by P_porin194_ promoter (Figure 2c), which encode dihydric alcohol reductase, aldehyde dehydrogenase and 4HB‐CoA transferase, respectively, were constructed on pSEVA321 vector in TD80, namely TD80‐D, to synthesize 3HP, 4HB and 5HV monomers from structure‐related α,ω‐diols (1,3‐Propanediol, 1,3‐PDO; 1,4‐Butanediol, 1,4‐BDO; 1,5‐Pentanediol, 1,5‐PDO) for the co‐polymerization with native 3HB monomer (Figure 2a). Therefore, different PHA copolymers, namely PHBP, P34HB, and P3HB5HV, were obtained by recombinant TD80‐D grown in 500‐mL shake flasks containing 50‐mL 50MMA medium (35 g L^−1^ acetate) supplemented with 0, 5, and 10 g L^−1^ α,ω‐diols, respectively (Figure 2d). Notably, TD80‐D displayed better performance in 4HB monomer accumulation with a molar ratio reaching 4.6 mol%, which was over 3‐ and 5‐fold higher than 3HP and 5HV units, respectively. Besides, the recombinant TD80‐D strain grown on acetate and γ‐butyrolactone (GBL) as 4HB precursor showed improved performance in DCM and P34HB accumulation, yielding 8.41 g L^−1^ DCM containing 70.9 wt.% P(3HB‐*co*‐10.5 mol% 4HB) (Figure S6, Supporting Information).^[^ ^29^ ^]^ Obviously, TD80 inherited the excellent capability for P34HB synthesis from its mother host TD1.0.^[^ ^29^, ^42^, ^43^, ^44^ ^]^ Furthermore, the de novo synthesis pathway of 4HB monomer encoded by *4hbd*, *sucD*, *odgA*, and *orfZ* genes, which was grouped into two modules, namely *4hbd*‐*sucD*‐*odgA* and *orfZ*, driven by IPTG‐induced P_MmP1_ promoter and P_porin194_, respectively, was constructed in TD80 for P34HB synthesis from acetate only (Figures 2a,e). Varied expression levels of *4hbd*‐*sucD*‐*odgA* were first assessed in a shake flask study in the presence of 0, 2, 10, 20, 100, and 200 mg L^−1^ IPTG, respectively (Figure 2f). The highest 4HB molar ratio (8.0 mol%) was obtained by recombinant TD80 induced by 100 mg L^−1^ IPTG. Subsequently, the P_porin194_ promoter of similar expression strength was used to replace P_MmP1_, forming TD80‐GB strain (Table S2, Supporting Information), for more robust P34HB synthesis under inducer‐free conditions, which resulted in 8.5 mol% 4HB accumulation in the obtained P34HB using acetate as a sole carbon source (Figure 2f). Followed by triplicate fed‐batch studies by TD80‐GB conducted in a 7‐L bioreactor, over 12 g L^−1^ P(3HB‐*co*‐10.0 mol%4HB) was successfully obtained after 32‐h fermentation with DCM and PHA content reaching over 30 g L^−1^ and 40 wt.%, respectively (Figure 2g; Figure S7, Supporting Information). The above results thus provide promising foundations for varied PHA production synthesis by recombinant TD80 from acetate only.

In addition to PHA synthesis, 3HB, the monomer of PHB, which has great uses in healthcare and disease treatment, such as seizures, hypertension, NLRP3‐mediated inflammation, and neurodegenerative diseases,^[^ ^45^, ^46^ ^]^ is also a candidate compound for recombinant TD80 to produce. First, the *phaC*_1_ gene encoding PHA polymerase in TD80 was knocked out to block the accumulation of PHA, generating the TD80‐P strain. Subsequently, the 3HB synthesis pathway encoded by the *phaA* and *phaB* genes from different microbes, and the *tesB* gene from *E. coli* MG1655 genes, were grouped into one expression module controlled by the P_MmP1_ promoter, namely *phaA‐phaB‐tesB*, and constructed on the pSEVA321 vector (Figure 2h). Particularly, *phaA*_RE_ and *phaB*_RE_ genes from *Ralstonia eutropha* H16 exhibit better activity on 3HB synthesis from both glucose and acetate compared with the ones (*phaA*_TD_ and *phaB*_TD_) from *Halomonas* TD (Figures S8a,b, Supporting Information). Hence, the expression fine‐tuning of the *phaA*_RE_ *‐phaB*_RE_ *‐tesB* module in TD80‐P was carried out to achieve optimum 3HB production titer, reaching 5.5 g L^−1^ under 2 mg L^−1^ IPTG induction, via controlling the supplemented dosage of IPTG (Figure 2i; Figure S8c, Supporting Information). Furthermore, the P_porin281_ promoter with similar strength to P_MmP1_ induced by 2 mg L^−1^ IPTG was then used to express the *phaA*_RE_ *‐phaB*_RE_ *‐tesB* module in recombinant TD80 for inducer‐free 3HB synthesis, namely TD80‐B. Finally, 5.6 g L^−1^ 3HB was obtained by TD80‐B grown in 500‐mL shake flasks using acetate only. Besides, ≈1‐fold improvement of 3HB titer (11.0 g L^−1^), was achieved by TD80‐B after 36 h growth in a 7‐L bioreactor (Figure 2j).

### 2.3 Engineering TD80 for Diverse Bioproducts Synthesis from Acetate

**Figure 3:**
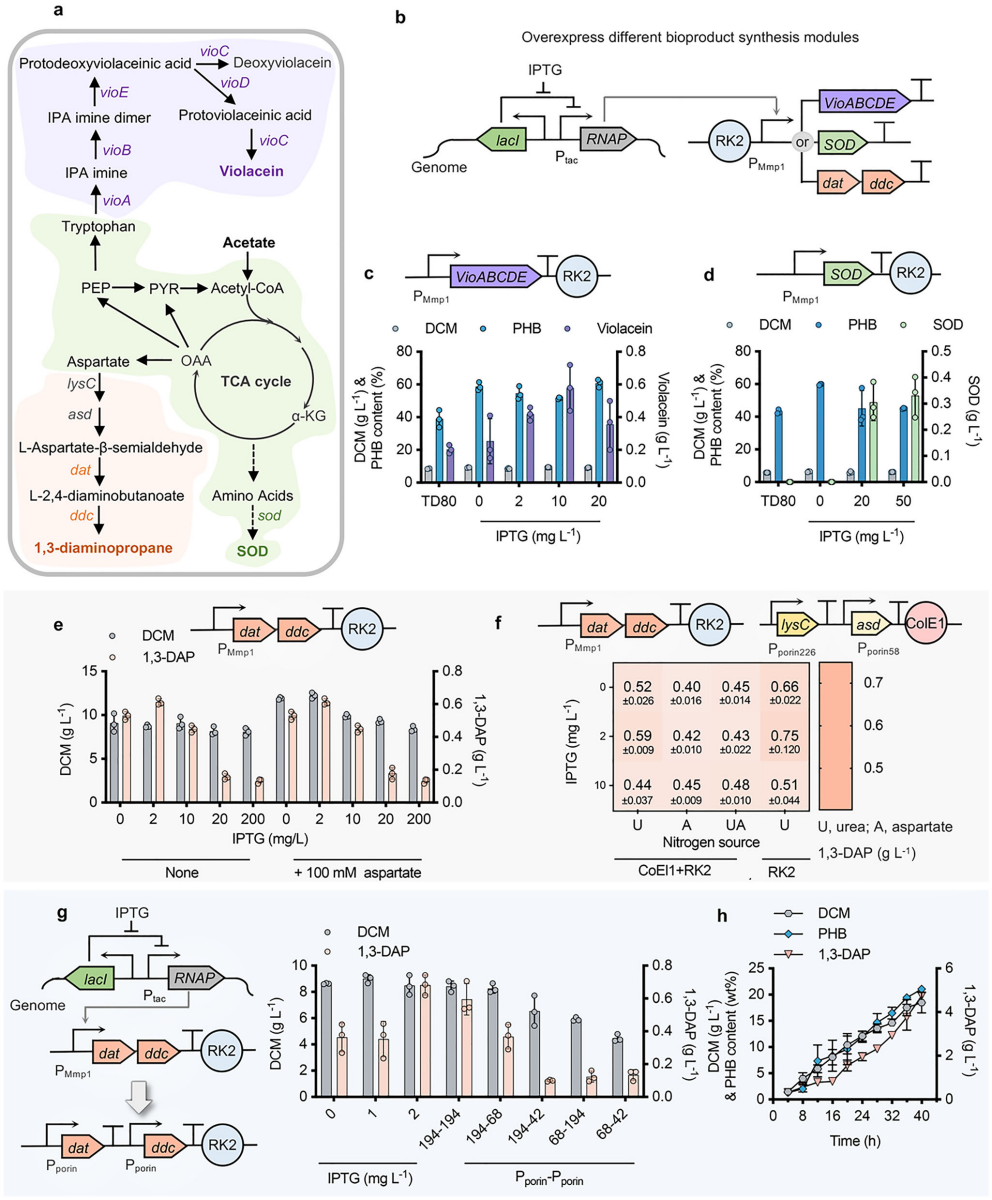
Engineering TD80 for diverse bioproducts synthesis from acetate. a) Metabolic pathway design for violacein, SOD, and 1,3‐DAP synthesis by recombinant TD80. b) Schematic diagram of promoter engineering strategy to tune different modules for violacein (*vioABCDE*), SOD (*sod*), and 1,3‐DAP (*dat‐ddc*) synthesis directed by P_T7‐like_ induced by IPTG. c, d) DCM, PHB, violacein (c) and SOD (d) production by recombinant TD80 harboring *vioABCDE* and *SOD* module, respectively, induced by a spectrum of IPTG grown in 50MMA medium (1 g L^−1^ urea). e) DCM and 1,3‐DAP accumulation by recombinant TD80 harboring the *dat‐ddc* module induced by a spectrum of IPTG in 50MMA medium in the presence of 3 g L^−1^ urea (None group) and 100 mm aspartate, respectively. f) Comparative analysis of 1,3‐DAP synthesis by recombinant TD80 harboring *dat‐ddc* module with (CoEl1+RK2) and without (RK2) *lysC‐asd* cluster overexpression. U, 3 g L^−1^ urea; A, 100 mm aspartate; UA, combination of U and A. g) Replacement of constitutive P_porin_ promoter for *dat‐ddc* expression instead of IPTG‐induced P_T7‐like_. h) Fed‐batch study for de novo synthesis of 1,3‐DAP by recombinant TD80 (194‐194) group in part (e) conducted in a 7‐L bioreactor. *vioABCDE*, tryptophan oxidase (A), indole‐3‐pyruvate (B), decarboxylase (C), oxidoreductase (D), cyclase and oxygenase (E); *SOD*, superoxide dismutase; *lysC*, aspartokinase; *asd*, L‐aspartate‐β‐semialdehyde dehydrogenase; *dat*, 2‐ketoglutarate 4‐aminotransferase; *ddc*, L‐2,4‐diaminobutanoate decarboxylase. Error bars represent standard deviations, n = 3 (for fed‐batch studies, *n* = 2).

In addition to biodegradable PHAs and 3HB, reducing the life cycle CO_2_ footprint of different bioproducts, such as high‐value proteins (SOD^[^ ^47^ ^]^), amino acid derivatives (violacien),^[^ ^48^ ^]^ and polyamide monomers (eg., 1,3‐DAP,^[^ ^49^ ^]^ putrescine,^[^ ^50^ ^]^ cadaverine^[^ ^51^ ^]^), are also an attractive requirement for industrial sustainability. Thereinto, effective production of SOD and violacein has been achieved in *Halomonas* in previous studies.^[^ ^47^, ^48^ ^]^ Besides, 1,3‐DAP derived from aspartate, an important intermediate for ectoine synthesis to resist the environmental stress in *Halomonas*,^[^ ^32^, ^33^ ^]^ is of great potential to be produced by engineered TD80 from acetate (**Figures** **3**a,b). For prototyping production of violacein and SOD, the key synthesis clusters encoded by the *vioABCDE* operon (*Chromobacterium violaceum*) and *sod* (*E. coli*), respectively, were constructed in TD80 controlled by IPTG‐induced P_MmP1_ independently. In the shake flask study, 0.6 g L^−1^ violacein and 0.3 g L^−1^ SOD can be obtained by recombinant TD80 grown on acetate only in the presence of 10 and 50 mg L^−1^ IPTG, respectively (Figures 3c,d; Figure S9, Supporting Information). Additionally, ≈40 to 50 wt.% of PHB within the DCM was co‐produced while the synthesis of violacein and SOD.

For 1,3‐DAP synthesis, a heterogenous gene cluster including genes *dat* encoding 2‐ketoglutarate 4‐aminotransferase and *ddc* encoding L‐2,4‐diaminobutanoate decarboxylase was constructed in TD80 under the control of P_MmP1_ (ultra‐high induction output) and P_tac_ (low induction output) promoters, respectively, to achieve fine‐tuned expression purpose under customized dynamic range induced by different concentrations of IPTG (Figure 3b; Figure S10, Supporting Information). Shake flask results showed that over a 2‐fold increase of 1,3‐DAP (≈0.7 g L^−1^) can be obtained by recombinant TD80 harboring the *dat‐ddc* module driven by P_MmP1_ compared to that controlled by P_tac_ grown in the same condition under 2 mg L^−1^ IPTG induction (Figure S10, Supporting Information). Unexpectedly, medium optimization, including additional urea (5 and 7 g L^−1^) and aspartate (100 mm) supplementation, or enhanced supply of L‐aspartate‐β‐semialdehyde by overexpressing *lysC* and *asd* genes (Figures 3e,f; Figure S11, Supporting Information), could not boost the synthesis of 1,3‐DAP. Instead, the supplementation of aspartate led to increased accumulation of DCM and by‐product ectoine, which competes with the same upstream synthesis pathway against 1,3‐DAP (Figure 3a; Figure S11c, Supporting Information). Subsequently, according to the expression level of P_MmP1_ induced by 2 mg L^−1^ IPTG, three constitutive promoters of higher (42, P_porin42_), similar (68, P_porin68_), and lower (194, P_porin194_) strength were selected to drive *dat* and *ddc* genes, respectively, aiming to obtain an inducer‐free recombinant for 1,3‐DAP synthesis (Figure 3g). Finally, the optimal combination ‘194‐194′, namely recombinant TD80‐DD, yielded 0.6 g L^−1^ 1,3‐DAP in shake flask study, which is close to the best‐induced group (0.7 g L^−1^) under 2 mg L^−1^ IPTG induction. Fed‐batch study by TD80‐DD was carried out in a 7‐L bioreactor using acetate as the sole carbon source. Over 4.8 g L^−1^ 1,3‐DAP was obtained after 40 h of fermentation, which is 8‐fold higher compared to that conducted in a shake flask (Figure 3h). The success of 1,3‐DAP synthesis by engineered TD80 from acetate exemplifies the great possibility for bio‐based polyamide manufacturing of reduced CO_2_ footprint.

### 2.4 Engineering TD80 for the Co‐Production of Ectoine and PHB from Acetate

**Figure 4:**
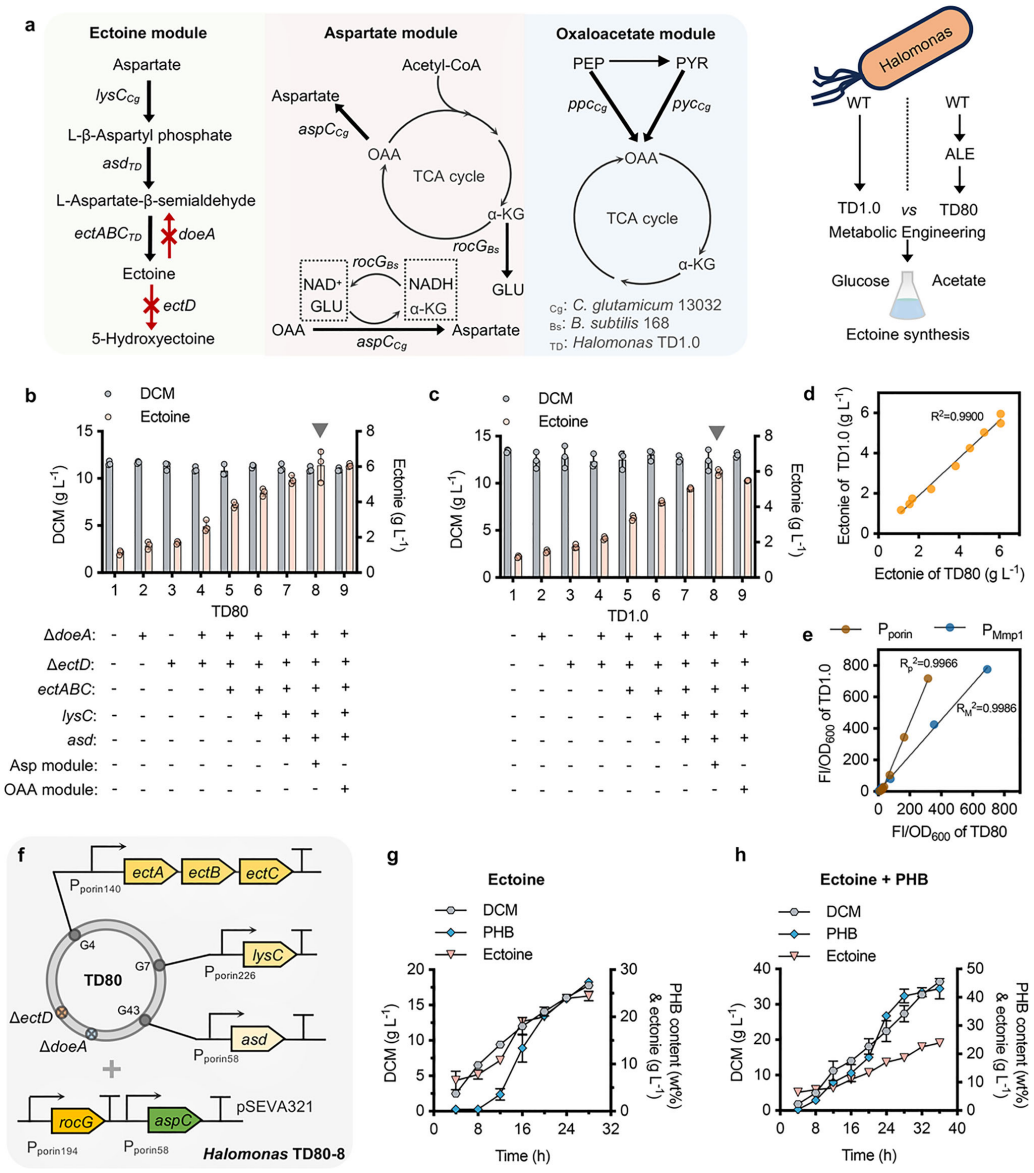
Engineering TD80 for the co‐production of ectoine and PHB. a) Metabolic engineering of TD80 for ectoine synthesis, consisting of three parts: the ectoine synthesis module, the aspartate and oxaloacetate supply modules. The black arrows in bold indicate gene overexpression, and the arrows in red with “×” indicate gene deletion, aiming to achieve reinforced fluxes and cofactors for ectoine synthesis. Schema of engineering ALE‐evolved TD80 for ectoine synthesis form acetate compared to recombinant TD1.0 from glucose. b, c) Effects on ectoine synthesis by introducing three metabolic engineering modules in recombinant TD80 (b) and TD1.0 (c). d) Linear correlation analysis of ectoine titers by recombinant TD80 strains (*x*‐axis, from part b) plotted against the ones by recombinant TD1.0 strains (*y*‐axis, from part c). e) Linear correlation analysis of different expression levels of sfGFP in TD80 (*x*‐axis) and TD1.0 (*y*‐axis) driven by P_porin_ promoter mutants (dots in brown) and P_MmP1_ induced by different concentrations of IPTG (dots in blue), respectively. Fluorescence intensity (FI) was normalized by dividing by OD_600_. f) Schema of constructing TD80‐8 strain by integrating ectoine synthesis (chromosome‐based) and aspartate supply (plasmid‐based) modules enables robust and effective biosynthesis of ectoine. g, h) Fed‐batch study for ectoine production, as well as coproduction of ectoine and PHB, by TD80‐8 conducted in a 7‐L bioreactor. *ectA*, L‐2,4‐diaminobutyrate acetyltransferase; *ectB*, L‐2,4‐diaminobutyrate transaminase; *ectC*, ectoine synthase; *doeA*, ectoine hydrolase; *ectD*, ectoine hydroxylase; *rocG*, glutamate dehydrogenase; *aspC*, aspartate aminotransferase; *pyc*, pyruvate carboxylase; *ppc*, phosphoenolpyruvate carboxylase. Error bars represent standard deviations, *n* = 3 (for fed‐batch studies, *n* = 2).

As mentioned above, ectoine is an important osmolyte synthesized by most halophilic bacteria to resist saline and alkaline stress. Recently, the biosynthesis of ectoine has been achieved by recombinant *Halomonas* TD,^[^ ^32^, ^33^ ^]^ *E. coli*,^[^ ^52^, ^53^ ^]^ *C. glutamicum*,^[^ ^54^ ^]^ etc. due to its growing market use in cosmetics, food, medicine, and so on.^[^ ^55^ ^]^ Therefore, engineering TD80 for ectoine synthesis would be a win‐win strategy for recombinant cells to grow with enhanced ectoine synthesis to resist the increased sodium salt accumulation resulting from the feeding of acetate (or CDE). Herein, three metabolic engineering modules, namely ectoine, aspartate (Asp), and oxaloacetate (OAA) modules, were designed to achieve reinforced fluxes toward ectoine synthesis (**Figure** **4**a). Specifically, the ectoine module includes: knocking out the degradation bypasses encoded by *doeA* and *ectD* genes, overexpression of the key synthesis pathway for reinforced flux from aspartate toward ectoine encoded by *lysC*_Cg_ gene from *C. glutamicum*, *asd*_TD_ and *ectABC*_TD_ genes from *Halomonas* TD; the Asp module includes: overexpression of *aspC*_Cg_ gene from *C. glutamicum* for enhanced aspartate supply from OAA and *rocG*_Bs_ gene from *B. subtillis* for improved NADH regeneration; the OAA module includes: overexpression of *ppc*_Cg_ and *pyc*_Cg_ genes from *C. glutamicum* for enhanced OAA supply.

First, each design of the ectoine module was implemented one by one to obtain chromosomally engineered strains derived from TD80, namely TD80‐2, TD80‐3, TD80‐4, TD80‐5, TD80‐6 and TD80‐7, respectively, according to the previous study reported by Ma et al.^[^ ^32^ ^]^ Subsequently, the expression design of genes involved in Asp and OAA modules was individually pre‐tested and fine‐tuned using P_MmP1_ and P_lux_ promoters induced by different combinatory dosages of IPTG and OC6 in TD80‐7 grown in 50MMA medium containing 3 g L^−1^ urea. This aimed to determine the optimum induction level for ectoine synthesis and then directed the constitutive promoter replacement by the P_porin_ mutant of the same strength (Figures S12a,b and S13a,b, Supporting Information). Two optimized constructs, including p321‐P_porin194_‐*rocG*_Bs_‐P_porin58_‐*aspC*_Cg_ from the Asp module and p321‐P_porin68_‐*ppc*_Cg_‐P_porin42_‐*ppc*_Cg_ from the OAA module, harbored by TD80‐7, namely TD80‐8 and TD80‐9, respectively, displaying optimum ectoine synthesis performance, were thus obtained for further fermentation test incorporating of ectoine module (Figures S12c and S13c, Supporting Information). Besides, 5 g L^−1^ of urea addition was found to be the optimal condition for ectoine synthesis in a shake flask study (Figure S14, Supporting Information). Therefore, comparative analysis of ectoine production was carried out by TD80 and its derivatives grown in 50MMA medium containing 5 g L^−1^ urea in a 500‐mL shake flask (Figure 4b). The titer of ectoine by TD80‐7 harboring a genomically integrated ‘ectoine module’ reached up to 5.3 g L^−1^, which was increased by 4.7‐fold compared to TD80. Over 15% improvement in titer was further achieved by TD80‐8 and TD80‐9 harboring Asp and OAA module, respectively, on plasmid‐carried system derived from TD80‐7, leading to over 6 g L^−1^ ectoine accumulation from acetate. However, the combination of three modules led to an obvious decrease in ectoine synthesis, probably due to the heavy burden of the double‐plasmid system (Figure S15, Supporting Information). In particular, to access the effect of carbon source replacement from glucose to acetate on ectoine production, each design involved in the three engineering modules was introduced into TD1.0 one by one following the same procedure to obtain 8 recombinants (TD1.0‐2 to TD1.0‐9 listed in Table S2, Supporting Information) for shake flask fermentation test under the same condition except using glucose as carbon source (Figures 4c,d). Moreover, constitutive and inducible promoters used in this study were also characterized in TD80 and TD1.0 using *sfGFP* as a reporter (Figure 4e). Expectedly, both the ectoine production titer and fluorescent intensity by recombinant TD80 and TD1.0 strains, respectively, showed a highly linear correlation with *Pearson* coefficient (*R* ^2^) reaching over 0.99 (Figures 4d,e). These results suggested that TD80 grown on acetate manifests negligible effect on gene expression activity, characterized by sfGFP,^[^ ^56^ ^]^ and ectoine‐synthesis capability when compared to its starting host TD1.0 grown on glucose, enabling diverse metabolic engineering for desired bioproduction synthesis by recombinant TD80 from acetate.

Finally, the best‐performing recombinant, TD80‐8 (Figure 4f), was selected for a fed‐batch fermentation test conducted in a 7‐L bioreactor. After 28 h cultivation, 24.4 g L^−1^ ectoine was obtained by TD80‐8 using acetate only (Figure 4g). Co‐production of ectoine and PHB were further studied by TD80‐8 via adjusting the feeding solution with reduced urea addition (see method) to yield 23.9 g L^−1^ ectoine and 17.6 g L^−1^ PHB (DCM, 41.0 g L^−1^; PHB content, 43.0 wt.%) after 36 h fermentation (Figure 4h), displaying better performance compared to the previous study (8.0 g L^−1^ ectoine & 24.0 g L^−1^ PHB) using glucose as carbon source.^[^ ^32^ ^]^

### 2.5 Engineering Reinforced PHB Synthesis to Achieve Improved CDE‐to‐Product Conversion for Industrial Scale‐Up Purpose

**Figure 5:**
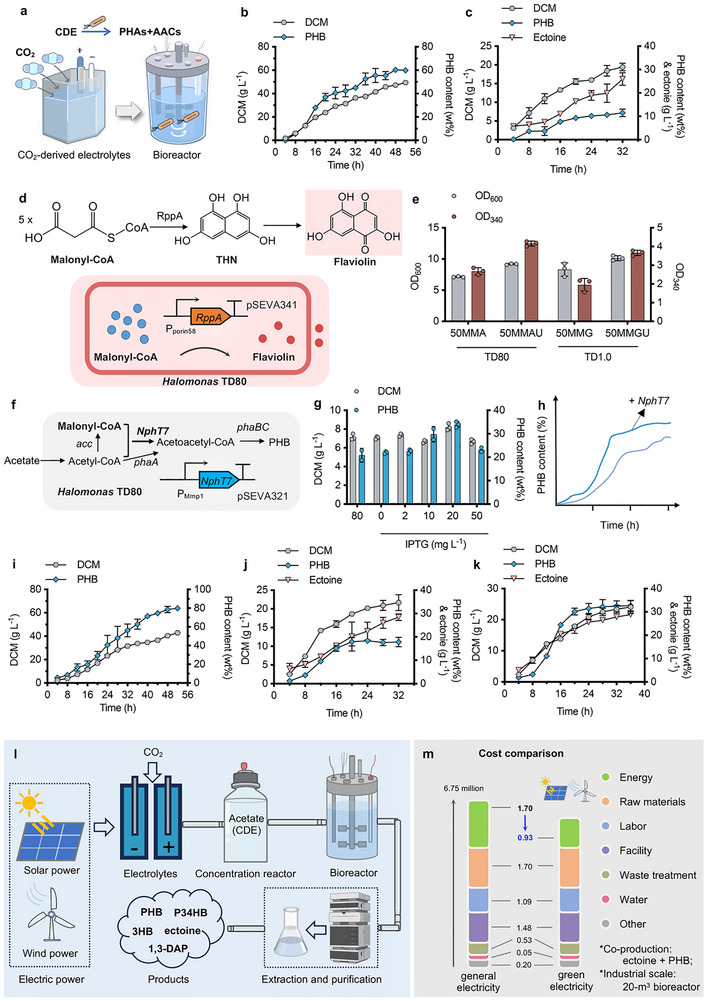
Biosynthesis assessment by recombinant TD80 strains grown on CDE. a) Schematic diagram for PHB and ectoine biosynthesis using CDE as a sole carbon source conducted in a 7‐L bioreactor. b) Fed‐batch study for PHB production by TD80 conducted in a 7‐L bioreactor using CDE as the sole carbon source. c) Fed‐batch study for the co‐production of ectoine and PHB by recombinant TD80‐8 was conducted in a 7‐L bioreactor using CDE as the sole carbon source. d) Biosensing design for quantitative analysis of malonyl‐CoA accumulation by engineered TD80 harboring the RppA encoded gene, a type III polyketide synthase able to convert five molecules of malonyl‐CoA into one molecule of flaviolin in red. e) Comparative analysis of OD_600_ (cell growth) and OD_340_ (malonyl‐CoA level) by both recombinant TD80 and TD1.0 harboring *rppA* expression module controlled by P_porin58_ grown in 50MMA medium (35 g L^−1^ acetate, 0.6 L^−1^ urea), 50MMAU medium (35 g L^−1^ acetate, 3 g L^−1^ urea), 50MMG medium (30 g L^−1^ glucose, 0.6 L^−1^ urea) and 50MMGU medium (30 g L^−1^ glucose, 3 g L^−1^ urea), respectively. f) Schematic diagram of a non‐canonical PHB biosynthesis pathway by recycling malonyl‐CoA flux mediated by NphT7, an acetoacetyl‐CoA synthase from *Streptomyces sp*. g) Shake flask studies of DCM and PHB content by TD80‐N harboring NphT7 expression module driven by P_MmP1_ promoter grown on 50MMAU medium in the presence of different IPTG concentrations. h) Schematic accumulation patterns of difference by recombinants with and without non‐canonical PHB synthesis pathway. i) Fed‐batch studies for PHB production by TD80‐N conducted in a 7‐L bioreactor. j) Fed‐batch studies for ectoine production by recombinant TD80‐8‐R conducted in a 7‐L bioreactor. k) Fed‐batch studies for co‐producing ectoine and PHB by recombinant TD80‐8‐R were conducted in a 7‐L bioreactor. l) Flow chart design for cost‐effective microbial electrosynthesis from CO_2_‐derived electrolytes toward different value‐added products by using wind and solar power. m) Techno‐economic assessment (TEA) of co‐production of ectoine and PHB based on scale‐up fermentation conducted in a 20‐m^3^ bioreactor using green electricity. Error bars represent standard deviations, *n* = 3 (for fed‐batch studies, *n* = 2).

Compared with the sodium acetate used for strain development in previous studies (Figures 2, 3, 4), the CDE used in this study is a mixture solution containing acetate (90 g L^−1^) and a small percentage of formic acid (10 g L^−1^) and carbonate (0.8 g L^−1^), which was acquired from a three‐step cascaded electrochemical catalytic system provided by Power Carbon Co., Ltd. (Figures S16–S18, Supporting Information). To validate the feasibility of direct CDE utilization by recombinant TD80 strains for various bioproducts synthesis like PHA, 3HB, 1,3‐DAP and ectoine (**Figure** **5**a; Figure S19, Supporting Information), it is thus significance to integrate two independent systems, including CDE generation system and multi‐production synthesis platform, to build a prototyping electrochemical‐biological hybrid system able to convert CO_2_ into value‐added products (Figure 5a; Figure S19a, Supporting Information). First, the cell mass accumulation by TD80 using CDE (or acetate) as a sole carbon source rather than other components in 50 MM medium was carefully verified again (Figure S19b, Supporting Information). Then, shake flask studies were carried out by five recombinants, including PHB producer TD80 and its derivatives, namely TD80‐GB, TD80‐B, TD80‐DD, and TD80‐8 for P34HB, 3HB, 1,3‐DAP ectoine synthesis, respectively, grown on CDE (Table S7, Supporting Information) and acetate, respectively, for comparative assessment of biosynthesis performance. Consistently, in contrast to the groups using acetate as a carbon source, the obtained CDE solution showed a negligible effect on the cell growth and production synthesis (Figures S19b–g, Supporting Information). Specifically, only a slight titer reduction of 1,3‐DAP (‐7.8%) and ectoine (‐5.3%) was observed, while the titers of PHB and 3HB were increased only by 2.7% and 4.8%, respectively (Figures S18f,g, Supporting Information). To verify the scale‐up feasibility of different products by recombinant TD80 grown on CDE only (Figure S20, Supporting Information), fed‐batch studies of violacein and SOD were conducted in a 7‐L bioreactor, yielding 0.7 g L^−1^ violacein (co‐producing 6.2 g L^−1^ PHB) and 1.2 g L^−1^ SOD (co‐producing 12.1 g L^−1^ PHB), respectively, after 28 h fermentation (Figure S21, Supporting Information). Particularly, fed‐batch study of PHB and ectoine with higher production yields by TD80 and TD80‐8 strain, respectively, were further investigated in a 7‐L bioreactor using CDE as the sole carbon source (Figure 5a; Figure S20, Supporting Information). Finally, 29.6 g L^−1^ PHB (49.4 g L^−1^ DCM containing 60.0 wt.% PHB) and 26.0 g L^−1^ ectoine were obtained after 52 and 32 h fermentation, respectively (Figures 5b,c). However, more attempts are still required to improve the PHB content to meet the demand of industrial uses.^[^ ^57^ ^]^

The PHA fermentation process is generally divided into two phases: the cell growth phase to reach a maximized cell population under rich‐nitrogen conditions and the PHA accumulation phase under limited‐nitrogen conditions, by modulating the carbon‐nitrogen ratio of the feeding solution.^[^ ^58^ ^]^ Therefore, the timing control of acetyl‐CoA toward cell mass synthesis or PHB accumulation is a key turning point to achieve high cell‐density fermentation (Figures 2a and 5h). The TCA cycle metabolism and malonyl‐CoA‐derived metabolites synthesis (polyketides, fatty acids, etc.) that support cell growth are dominant flux consumers of acetyl‐CoA.^[^ ^59^ ^]^ Therefore, the 1,3,6,8‐tetrahydroxynaphthalene synthase encoded by *rppA* was employed to construct an enzyme‐mediated malonyl‐CoA biosensor, converting malonyl‐CoA to flaviolin of red color can be quantitatively determined by OD_340_,^[^ ^59^ ^]^ to assess the surplus levels of malonyl‐CoA by TD80 and TD1.0 grown on rich‐ and limited‐nitrogen conditions (Figures 5d,e). Obviously, the malonyl‐CoA level by TD80 grown on acetate was higher than TD1.0 grown on glucose. Rich nitrogen conditions could significantly boost the surplus level of malonyl‐CoA, with OD_340_ value increased by over 55% either in TD80 or TD1.0 groups (Figure 5e). Recycling the surplus flux of malonyl‐CoA for PHB synthesis was thus proposed to improve the PHB content with a higher CCR value (Figure 5f). The *nphT7* gene from *Streptomyces* sp.^[^ ^60^ ^]^ encoding acetoacetyl‐CoA synthase was constructed in TD80 under the control of P_MmP1_, namely TD80‐N, to achieve joint forces for acetoacetyl‐CoA synthesis with the *phaA* gene. The expression of the *nphT7* gene in TD80‐N was fine‐tuned by supplementing different concentrations of IPTG during the shake flask study. The highest PHB content by TD80‐N grown in rich‐nitrogen medium (50MMAU) was achieved in the presence of 20 mg L^−1^ IPTG, which was over 70% higher than the control one (80) (Figure 5g). Therefore, this strategy was adopted for fed‐batch studies of PHB (Figure 5i), ectoine (Figure 5j), and their co‐production (Figure 5k), respectively. Expectedly, the PHB content of all groups was increased to varying degrees: from 60.0 to 79.9 wt.% for PHB synthesis (Figures 5b,i); from 11.5 to 17.9 wt.% for ectoine synthesis (Figures 5c,j); and 32.7 wt.% for co‐production (Figure 5k). More importantly, the CCR of CDE‐to‐PHB and CDE‐to‐PHB/ectoine were increased by 15.0% and 10.6%, respectively, compared to the groups without malonyl‐CoA recycling (Table S8, Supporting Information). In contrast to the productions from either CO_2_‐derived substrates or glucose, these results manifest strong competitiveness in carbon conversion rate and productivity (Table S8, Supporting Information). Therefore, our study exemplified promising advancement in chassis development to enhance the biosynthesis capacity and efficiency of an electrochemical‐biological hybrid system for industrially available CO_2_ upcycling.

Besides, a technology and economy assessment (TEA) model based on a 20‐m^3^ pilot‐scale plant (data generated from Hefeng Co., Ltd., Zhanjiang, Guangdong Province) was established to evaluate the industrial scale‐up feasibility of co‐producing ectoine and PHB by TD80‐8 and TD80‐8‐N from CDE according to the fed‐batch results conducted in a 7‐L bioreactor (Figures 4h and 5k; Data S1, Supporting Information). Despite the cost reduction of energy input resulting from open and unsterile fermentation using halophilic recombinant TD80 as producer strain, TEA showed that electricity consumption, mainly for CDE generation (provided by Power Carbon Co., Ltd., Shenzhen, Guangdong Province), still holds nearly a quarter of the total cost. Therefore, utilizing green electric power, such as solar and wind power (as low as 0.30 ¥/kW), instead of conventional electricity to generate CDE could dramatically reduce the total cost by over 11.4% when the electricity price is lower than 0.3 ¥/kW (0.18 ¥/kW: 3267 ¥/t CDE, or < 459 $/t CDE; 0.3 ¥/kW: 4400 ¥/t CDE, or < 619 $/t CDE), which would be of great competitive advantage in industrial bio‐manufacturing compared to the traditional way using sugar as feedstock (e.g., 3500–4500 ¥/t glucose, or 500–632 $/t glucose) (Figures 5l,m; Data S1, Supporting Information). Certainly, engineering TD80 for enhanced CDE utilization with higher production yield is also an alternative strategy to achieve further cost reduction.

### 2.6 Engineering TD80 to Grow on Formate by Introducing the Reductive Glycine Pathway

**Figure 6:**
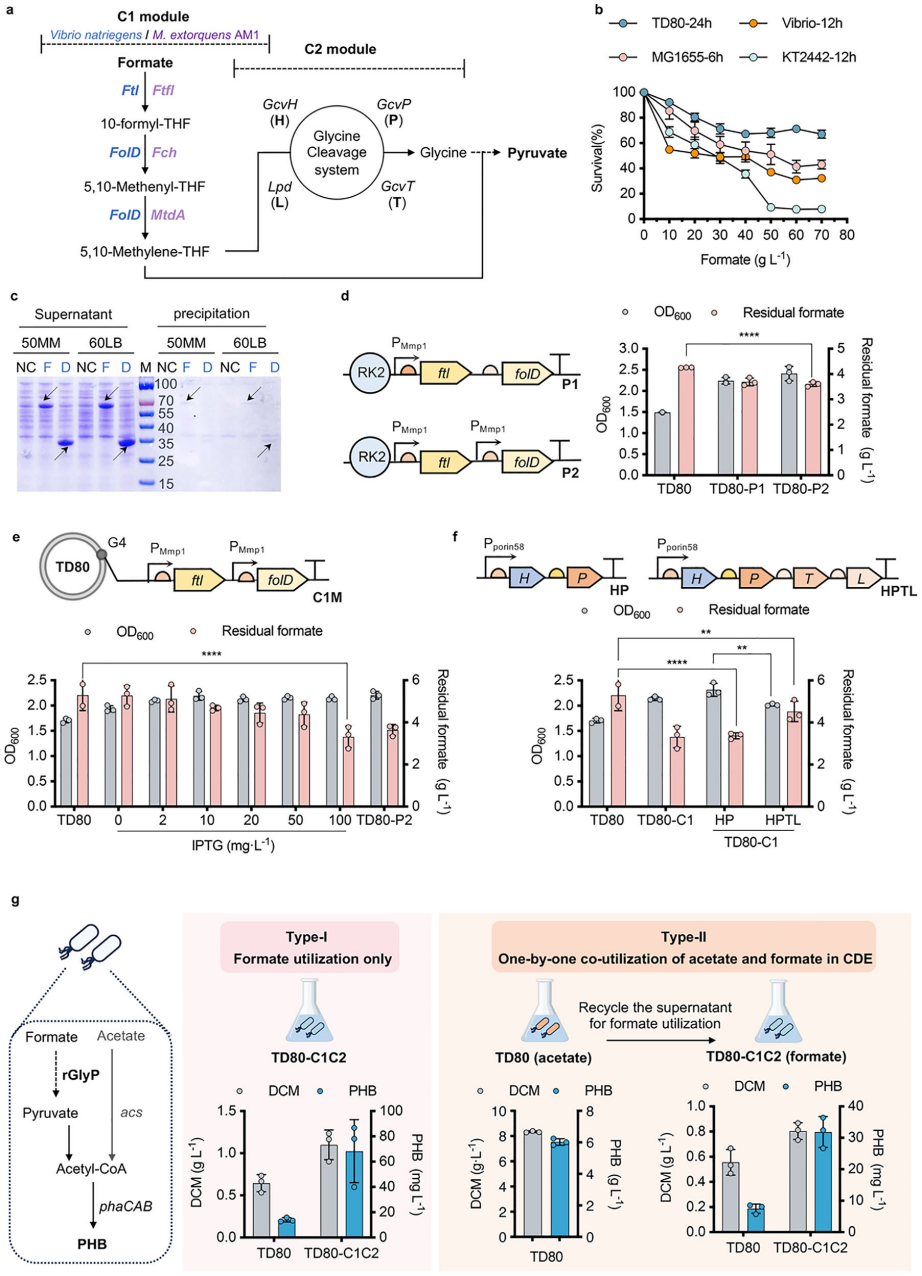
Engineering the formate utilization in TD80 based on the reductive glycine pathway. a) Schematic of the reductive glycine pathway‐based formate metabolism in TD80. The C1 module from *Methylobacterium extorquens* AM1 includes formate‐THF ligase (Ftl), formyl‐THF cycloligase (Fch), and methylene‐THF dehydrogenase (MtdA); the C1 module from *Vibrio natriegens* includes formate‐THF ligase (Ftl) and bifunctional cycloligase and dehydrogenase (FolD); the native C2 module includes glycine dehydrogenase (GcvP, P), glycine cleavage system aminomethyltransferase (GcvT, T), dihydrolipoamide dehydrogenase (Lpd, L), and glycine cleavage system protein (GcvH, H). b) Studying the formate tolerance of different strains, including TD80, *Escherichia coli* MG1655, *Vibrio natriegens*, and *Pseudomonas putida* KT2442. The cell density (OD_600_) of different strains grown in different concentrations of formate, from 0 to 70 g L^−1^, in MM medium (TD80, 50MM + 15 g L^−1^ acetate; MG1655 & KT2442, 10MM + 11 g L^−1^ glucose; *Vibrio*, 20MM + 11 g L^−1^ glucose) was recorded to calculate the survival rate (%) by dividing to each control group without formate addition. c) SDS‐PAGE analysis of the induced (50 mg L^−1^ IPTG) expression of Ftl and FloD (C1 module) in recombinant TD80 grown in 60LB and 50MMAU medium, respectively. d) Shake flask studies of OD_600_ and residual formate by recombinant TD80 harboring C1 module (P_MmP1_‐*ftl‐floD)* grown in 50MMFU (5 g L^−1^ formate, 3 g L^−1^ urea) medium in the presence of 2 mg L^−1^ IPTG. e) Comparative analysis of OD_600_ and formate utilization (residual concentration) by recombinant TD80 harboring P_MmP1_‐*ftl‐floD* on G4 locus (TD80‐C1) grown in 50MMFU (5 g L^−1^ formate, 3.0 g L^−1^ urea) medium in the presence of different IPTG concentrations. f) Shake flask studies of OD_600_ and formate utilization (residual concentration) by recombinant TD80‐C1 harboring different C2 module (GCS system) driven by P_porin58_ promoter (TD80‐C1C2) grown in 50MMFU (5 g L^−1^ formate, 3 g L^−1^ urea, 100 mg L^−1^). g) Schema and shake flask studies by TD80‐C1C2 grown on formate only, and harvested supernatant of fermentation broth by TD80 grown on CDE (acetate+formate) for further formate utilization that allows the full use of CDE. Error bars represent standard deviations, *n* = 3. ^*^ *p*<0.05; ^**^ *p*<0.01, ^***^ *p*<0.001.

Because formate is one of the major components within the CDE solution. Recently, the synthetic reductive glycine pathway (rGlyP) has been developed as an energy‐efficient route for formate assimilation in different chassis^[^ ^61^, ^62^, ^63^, ^64^ ^]^(**Figure** **6**a). Herein, the formate tolerance by four chassis, including TD80, *E. coli* MG1655, *Vibrio natriegens*, and *Pseudomonas putida* KT2442, was first characterized. Compared to the other three ones, TD80 exhibits the strongest tolerance to formate with a survival rate reaching 67% when grown at 70 g L^−1^ formate (Figure 6b; Figure S22, Supporting Information). Therefore, TD80 can be potentially engineered to grow on formate only. For most synthetic rGlyP systems, the C1 (converting formate into methylene‐tetrahydrofolate) and C2 (glycine cleavage system, GCS) modules are of great importance for formate utilization. First, two C1 modules from *Methylobacterium extorquens* AM1^[^ ^65^ ^]^ (AM1‐C1M) and from *Vibrio natriegens* ^[^ ^63^ ^]^ (Vio‐C1M) were constructed in *E. coli* BL21 for expression test via SDS‐PAGE analysis (Figure S23, Supporting Information). Subsequently, Vio‐C1M of a higher expression level was selected for hetergourous expression in TD80 under the control of IPTG‐induced P_MmP1_. The SDS‐PAGE results showed effective soluble expression of both Ftl and FloD (Figure 6c). Two plasmids containing *ftl* and *folD* genes controlled by P_MmP1_ simultaneously (P1, P_MmP1_‐*ftl*‐*folD*) and independently (P2, P_MmP1_‐*ftl*‐ P_MmP1_‐*folD*) were constructed in TD80 for growth test in 50MMFU medium containing 5 g L^−1^ formate and 3 g L^−1^ urea in the presence of 2 mg L^−1^ IPTG (Figure 6d). Additionally, the significantly improved cell growth via formate assimilation was further verified by recombinant TD80‐sfGFP. The introduction of the P2 construct, which exhibited better formate utilization and growth performance (Figure 6d), in TD80‐sfGFP led to an obvious increase in fluorescent intensity (Figure S24, Supporting Information).

The P_MmP1_‐*ftl*‐ P_MmP1_‐*folD* module in P2 was thus integrated on the G4 locus in TD80, namely TD80‐C1, which showed similar performance (100 mg L^−1^ IPTG) compared to the plasmid‐carried recombinant TD80‐P2 grown in the same condition (Figure 6e). Therefore, the chromosomally engineered TD80‐C1 with validated formate utilization capability provides a valuable foundation for subsequent C2 module engineering.

According to the transcriptomic result reported in a previous study,^[^ ^66^ ^]^ the genes encoding glycine cleavage system (GCS, namely C2 module), including *gcvH*, *gcvP*, *gcvT*, and *lpd* genes,^[^ ^67^ ^]^ were identified in TD with active transcription (Figure 6a; Figure S25, Supporting Information). Besides, previous studies focusing on GCS^[^ ^68^, ^69^ ^]^ have shown that the carboxylation reaction catalyzed by GcvP (P) is a rate‐limiting step in GCS, and the increase of GcvH (H) level can enhance the synthesis rate effectively, whereas excess Lpd (L) and GcvT (T) showed a negligible effect on GCS or even worse. Therefore, two expression modules, including P_porin58_‐*gcvH*‐*gcvP* (HP) and P_porin58_‐*gcvH*‐*gcvP‐gcvT‐lpd* (HPTL), were constructed in TD80‐C1 for shake flask study. As shown in Figure 6f, the HP group, the best‐performing one, showed 8% improvement in OD_600_ compared to TD80‐C1. By contrast, the introduction of Lpd and GcvT in the HPTL group displayed a negative effect on both cell growth and formate utilization efficiency with higher residual formate concentration. Therefore, the recombinant TD80‐C1 harboring the HP module, namely TD80‐C1C2, was selected for further study on formate utilization (Figure 6g). Two strategies were thus designed for recombinant TD80‐C1C2 to use formate under different scenarios: Type I, using formate as a sole carbon source directly; Type II, one‐by‐one utilization of acetate and formate within CDE by TD80 and TD80‐C1C2, respectively. For the Type‐I test, TD80‐C1C2 was grown in 50 MM medium in the presence of 5 g L^−1^ formate and 0.6 g L^−1^ urea able to accumulate 1.1 g L^−1^ DCM and 68.3 mg L^−1^ PHB, which was over 71.9% and 384.4% higher than that by TD80 grown in the same condition. For Type‐II test, TD80 was first grown on CDE (30 g L^−1^ acetate + 3.3 g L^−1^ formate) for 48 h to consume the acetate, which yielded 8.3 g L^−1^ DCM and 6.0 g L^−1^ PHB; followed by centrifugation, the supernant of the fermentation broth mainly containing formate was recycled to culture TD80‐C1C2 for further formate utilization, yielding 0.8 g L^−1^ DCM and 31.9 mg L^−1^ PHB (Figure 6g).

In summary, TD80 was successfully engineered to use formate for PHB synthesis by introducing the C1 and C2 modules involved in the rGlyP system, demonstrating strong potential for the full use of CDE by recombinant *Halomonas* in the coming future.

## 3 Conclusion

In this study, an efficient electrochemical‐biological hybrid system for CO_2_ upcycling has been successfully developed by coupling the CDE generation module powered by three‐step cascaded electrocatalysis and the CDE utilization platform based on recombinant *Halomonas* TD80 strains. The ALE‐evolved chassis, *Halomonas* TD80, manifests dominant advantages in CDE (acetate) utilization for bioproduct synthesis. Different value‐added products of high‐level carbon storage capacity (g_CO2_/g_product_), such as biopolyesters like PHB and P34HB (2.1) with long carbon‐chain length, 3HB (1.7), ectoine (1.9), and 1,3‐DAP (1.8) (Table S9, Supporting Information), were successfully synthesized by engineered TD80 using CDE as a sole carbon source. Notably, after introducing a non‐canonical PHB synthesis pathway to recycle the surplus malonyl‐CoA derived from acetyl‐CoA flux, enhanced PHB accumulation with PHB content reaching 80 wt% (34.3 g L^−1^) can be obtained by recombinant TD80 from CDE only in fed‐batch studies (Figure 5i). Moreover, co‐producing PHB and ectoine was able to further increase the CCR of CO_2_ from 45.4 to 53.7 mol% (Figures 5j,k), indicating a higher carbon‐storage efficiency and capacity. Therefore, compared to the previous reports focusing on CO_2_ utilization based on electrocatalysis,^[^ ^6^, ^7^ ^]^ chem‐biological system,^[^ ^8^, ^9^ ^]^ electrochemical‐biological hybrid system and microbial electrocatalysis system,^[^ ^10^, ^13^, ^14^, ^70^ ^]^ our *Halomonas*‐based hybrid platform not only significantly expanded the production lines derived from CO_2_, but also dramatically enhanced the microbial synthesis performance from CDE including CDE‐to‐product's CCR, carbon‐storage capacity and productivity (Tables S8 and S9, Supporting Information) by reprograming the ALE‐evolved halophilic chassis TD80, which enables lower energy input under open and unsterile condition and stronger salt resistance against the continuous feeding of CDE.

More importantly, the establishment of the TEA model provides a brief overview of the cost structure for directing how to achieve further cost reduction in practical industrial processes, such as using green electricity to reduce power cost (Figure 5m). Furthermore, engineering TD80 to enhance the biosynthesis flux from CDE to targeted products, like PHA, violacein,^[^ ^48^ ^]^ SOD^[^ ^47^ ^],^ and ectoine,^[^ ^32^ ^]^ is a proven strategy to achieve higher‐level production titer and productivity, thereby further decreasing the energy consumption and labor requirements during industrial‐scale manufacturing. In addition to being an excellent PHA producer, *Halomonas* TD has been engineered to synthesize different compounds, such as 3‐hydroxypropionate (3HP),^[^ ^30^ ^]^ lysine and cadaverine,^[^ ^51^ ^]^ 5‐Aminovaleric acid (5‐AVA)^[^ ^71^ ^],^ etc., from sugars. Nevertheless, there is still great potential for the metabolic engineering of TD80 to produce more diversified products by leveraging synthetic and systems biotechnological tools. Generally, formate and ethanol are major byproducts generated in the electrolytes from Cell‐II and Cell‐III, respectively (Figures S15–S17, Supporting Information). Therefore, based on the success of engineering formate utilization (Figure 6), engineering TD80 for synergistic and enhanced utilization of acetate, formate^[^ ^61^, ^63^, ^72^, ^73^ ^]^ and ethanol^[^ ^74^, ^75^ ^]^ can not only achieve the full‐use of CDE, but also reduce the process complexity.

## 4 Experimental Section

### Strains, Plasmids, and Media

All strains used in this study are listed in Table S2 (Supporting Information). *Escherichia coli* S17‐1pir (*E. coli* S17‐1) was used as a conjugation donor and as a host for plasmid construction. *Halomonas* TD01 was isolated from Aydingol Lake of China, which was stored in CGMCC (China General Microbial Culture Collection Center, Beijing) numbered 4353. The derivative strain *Halomonas* TD1.0, starting chassis in this study, was chromosomally integrated with the RNA polymerase MmP1 expression module between the genomic loci GME_16862 and GME_16867.^[^ ^40^ ^]^ The strains generated from the ALE process were named the TDX series (“X” represents the defined generation time). Plasmids used in this study were assembled based on the vectors pSEVA321/341^[^ ^76^ ^]^ through the Gibson Assembly kit (NEB, Britain) from PCR‐amplified DNA fragments, which were purified via Universal DNA Purification Kit (TIANGEN, China) (Table S3, Supporting Information). All plasmids were purified using the TIANprep Mini Plasmid Kit (TIANGEN, China). The primer synthesis and DNA sequencing were performed by Songong Co., Ltd. (Shanghai, China). Primer design and sequence alignments were performed by SnapGene software (v6.0.2). Additionally, genome editing of *Halomonas* TD01 and its derivatives was performed using CRISPR/Cas9^[^ ^77^ ^]^‐ and/or homologous recombination^[^ ^78^ ^]^‐based methods developed in previous studies. The genes required to be synthesized or manipulated were listed in Table S4 (Supporting Information). The P_porin_ library was summarized in Table S5 (Supporting Information).

*E. coli* S17‐1 was cultured in Luria‐Bertani (LB) medium consisting of (g L^−1^) 10 tryptone, 5 yeast extract, and 10 NaCl. *Halomonas* TD01 and its derivatives were grown in a 60LB medium containing (g L^−1^) 10 tryptone, 5 yeast extract, and 60 NaCl, or mineral medium (50MMG) containing (g L^−1^) 50 NaCl, 30 glucose, 1 yeast extract, 0.6 urea. (3 urea for ectoine and 1,3‐DAP production), 0.2 MgSO_4_, 9.65 Na_2_HPO_4_· 12H_2_O, 1.5 KH_2_PO_4_, trace element solution I 10 mL·L^−1^ and trace element solution II 1 mL·L^−1^. The composition of trace element solution I is (g L^−1^) 5 Fe(III)‐NH_4_‐citrate, 2 CaCl_2,_ and 1 m HCl. The composition of trace element solution II was (mg L^−1^) 100 ZnSO_4_·7H_2_O, 30 MnCl_2_·4H_2_O, 300 H_3_BO_3_, 200 CoCl_2_·6H_2_O, 10 CuSO_4_·5H_2_O, 20 NiCl_2_·6H_2_O, 30 NaMoO_4_·2H_2_O and 1 m HCl. Especially, TD80 and its recombinant strains were cultivated in a 60LB medium supplemented with 20 g L^−1^ sodium acetate (NaAC), which is termed 60LBA medium, or 50MMA medium, which was derived from 50MMG medium by replacing 30 g L^−1^ glucose with 35 g L^−1^ NaAC. As for solid media, 2% (wt/vol) agar was added to the corresponding media. The pH of culture media was adjusted to 8.0–9.0 using a 5 m NaOH solution for shake flask and fed‐batch studies by *Halomonas* TD01 and its derivatives. Furthermore, ambenomycin (50 mg L^−1^), chloramphenicol (25 mg L^−1^) and/or spectinomycin (100 mg L^−1^) were included in the culture media when necessary to maintain the stability of target plasmids in different recombinant *Halomonas*.

### ALE‐Based Strain Screening

ALE of *Halomonas* TD1.0 was performed at 37 °C using 50MMA agar plates initially supplemented with 40 g L^−1^ NaAC. A single colony was isolated on a 60LBA agar plate and then spread on a 50MMA agar plate, followed by incubation at 37 °C for 48 h. Based on the characteristic of *Halomonas* TD producing high yields of PHB in 50MMA medium, the accumulation of PHB in the resulting monoclonals makes them exhibit regular shapes and appear notably white. Then the single colony with optimal cell growth and white on the 50MMA agar plates was chosen and spread on another new 50MMA agar plate every 48 h. The NaAC supplementation was increased in a stepwise manner from 40 to 100 g L^−1^ in a gradient of 20 g L^−1^ to achieve high‐NaAC tolerance. The standard for changing NaAC concentration was the cell morphology on the agar plate, when the single colony tends to be morphologically complete and translucent. After ALE, one of the TD80 colonies was selected on 50MMA agar medium supplemented with 100 g L^−1^ NaAC for further whole genome sequencing and other experimental analysis.

### Whole Genome Sequencing of Evolved Mutants

For whole genome sequencing of the evolved *Halomonas*, seed cultures of TD80 were inoculated from a single colony in 100 mL of 50MMA medium containing 35 g L^−1^ NaAC in 500‐mL conical flasks at 37 °C and cultivated for 12 h with shaking at 220 rpm. The cells were harvested by centrifugation (3000 rpm, 20 min, 4 °C; TGL‐16, Cence, China) and washed twice with sterile water to obtain the cell pellet. Subsequently, the samples were sent to BGI Genomics Co., Ltd. (Shenzhen, China) for genomic DNA extraction, library construction, and genome sequencing. Genomic DNA was extracted using the Tianamp Bacteria DNA Kit (TIANGEN, China). Library construction and sequencing were performed by PacBio (3rd) and DNBSEQ (2nd) platforms, and then de novo assembled into a complete genome.

### Conjugation

Constructed plasmids were transferred into *Halomonas* through a modified conjugation method^[^ ^36^ ^]^ using *E. coli* S17‐1 as donor cells. The donor cells harboring target plasmids were grown in LB medium with corresponding antibiotics overnight, while the recipient cells were cultured in 60LB medium to reach 0.6–0.8 of OD_600_. Both donor and recipient cells were harvested via centrifugation at 4500 rpm for 2 min, and washed twice with fresh 20LB medium containing (g L^−1^) 10 tryptone, 5 yeast extract, and 20 NaCl. After mixing at a ratio of 1:1, 50–100 µL of the cell mixture was dropped onto an antibiotic‐free 20LB agar medium at 37 °C for 6–8 h. Finally, the conjugated bacterial lawn was re‐suspended using 60LB medium and spread on a 60LBA agar plate containing relevant antibiotics for 48 h incubation to acquire positive colonies via PCR verification.

### Shake Flask and Fed‐Batch Studies

For shake flask studies, 1 mL of overnight seed cultures from a single colony was inoculated (5% vol) into 20 mL 50MMA medium supplemented with 35 g L^−1^ NaAC in 150‐mL conical flasks (OD_600_ reached 2.5 ± 0.2). 25 µg mL^−1^ chloramphenicol was added to the medium when necessary. After 48 h of incubation, 15 mL of the cell cultures were harvested via centrifugation at 4500 rpm for 10 min for analysis of dry cell mass (DCM), PHA content, 3HB, ectoine, acetate, and 1,3‐DAP.

For fed‐batch studies, 2.5 mL of overnight seed cultures (OD_600_ reached 2.5 ± 0.2) was inoculated in 50 mL 60LBA medium in 500 mL conical flasks for obtaining the 300‐mL secondary seed culture, which was used as the inoculum in a 7‐L bioreactor (T&J‐Intelli‐Ferm B 7L, China). The 4‐L basic cultural medium contains (g L^−1^) 2 MgSO_4_, 3.5 KH_2_PO_4_, 50 NaCl, 10 trace element solution I, and 1 trace element solution II, 12 yeast extract, 35 NaAC, and appropriate urea (1.6 urea for PHA and 3HB production; 3 urea for ectoine and 1,3‐DAP production). The fermentation process maintained dissolved oxygen (DO%) at ≈30% of air saturation by injecting air with a maximum flow rate of 1 vvm (air volume per culture volume per minute) and coupling with agitating at no more than 800 rpm. Additionally, the pH of culture media was adjusted using a 50% acetic acid solution. The feeding solution for the first phase (Feed‐I) comprises (g) 10.4 urea for PHA and 3HB production (36 urea and 8 yeast extract for ectoine, violacine, and 1,3‐DAP production; 13 urea for SOD) and 292 NaAC. Feed‐II consists of (g) 1.3 urea for PHA and 3HB production, 300 NaAC, and 1.5 (NH_4_)_2_SO_4_. During fermentation, the concentration of residual NaAC was monitored by high‐performance liquid chromatography (HPLC, LC‐16, Shimadzu, Japan) every hour and maintained within the range of 10–15 g L^−1^. Then, cell samples were harvested every 4 h for analyses of CDW, PHA, 3HB, acetate, ectoine, and/or 1,3‐DAP contents.

### DCM and PHA Content Assays

After 48 h of growth, cells were harvested via centrifugation at 12 000 rpm for 10 min and washed once with 20 mL of distilled water. DCM was determined using lyophilized cells after 24 h of vacuum freeze‐drying treatment. PHA content was evaluated using gas chromatography (GC‐2014 Pro, Shimadzu, Japan) following the method described by Tan et al.^[^ ^27^ ^]^ To determine PHA content, 20–30 mg of powdered lyophilized cells were sampled into a 15‐mL tube for esterification by adding 2‐mL chloroform and 2‐mL esterification solution (containing 97% v/v methanol, 3% v/v H_2_SO_4_ and 1 g L^−1^ benzoic acid) under 100 °C for 4 h. Then, after cooling to room temperature, 1 mL of distilled water was added for extraction and phase separation. Subsequently, 1 mL of the chloroform phase was sampled and further analyzed by GC. Additionally, 15–25 mg of PHB (Sigma–Aldrich) and 10–15 mg of γ‐butyrolactone (Sigma–Aldrich) were used as 3HB and 4HB standards, respectively.

### 4.1 3HB and Acetate Assays

For 3HB and acetate assays, 1 mL of the cell cultures was subjected to a 20‐fold dilution at the end of shake flask cultivation or a 50‐fold dilution during the fed‐batch study. For 3HB assays, the cells were then disrupted using an ultrasonic crusher (Supmile, KQ‐200VDE, CHN), followed by centrifugation at 12 000 rpm for 10 min, while for acetate assays, the sample only require centrifugation at 12 000 rpm for 10 min, Subsequently, the supernatants were filtered through a 0.22 µm filter membrane for 3HB and acetate quantification via HPLC equipped with an Aminex HPX‐87H column (300*7.8 mm, 9 µm, Bio‐Rad, USA) and a refractive index detector (RID‐20A). The mobile phase was 5 mm H_2_SO_4_ with a flow rate of 0.8 mL min^−1^, and the column temperature was maintained at 60 °C. 3HB was purchased from Sigma–Aldrich (USA) as a standard.

### 1,3‐DAP and Ectoine Assays

For 1,3‐DAP and ectoine assays, the supernatants were obtained as mentioned above (in section 3HB and Acetate Assays). Next, 20 µL of filtered supernatants was derivatized by diethyl ethoxymethylene malonate (DEEMM) for 1,3‐DAP quantification using a modified method described in a previous study.^[^ ^79^ ^]^ In detail, the DEEMM derivatization solutions containing (µL) 600 borate buffer (0.05 m, pH 9), 200 100% methanol, 170 distilled water, 20 supernatant samples, and 10 DEEMM (200 mm) were prepared and then heated at 70 °C for 2 h, followed by centrifugation at 12 000 rpm for 10 min after cooling. Subsequently, the treated solutions were filtered through a 0.22 µm filter membrane. The concentrations of 1,3‐DAP were determined via HPLC equipped with an Eclipse XDB‐C18 column (250*4.6 mm, 5 µm, Agilent, USA) and an UV detector (Essentia SPD‐16). The column temperature and detection were maintained at 35 °C and 284 nm, respectively. Two kinds of mobile phases were used for 1,3‐DAP measurement. Among them, mobile phase A was composed of a 25 mm sodium acetate buffer with a pH of 4.8, while mobile phase B comprised 100% acetonitrile. The assay was carried out at flow rate of 1 mL min^−1^ and a following gradient program of mobile phases A and B: 0–2 min, 80–75% A and 20–25% B; 2–32 min, 75–40% A and 25–60% B; 32–40 min, 40–80% A and 60–20% B. Additionally, for the ectoine assay, the supernatants of lysed cell samples were detected via HPLC with a C18 column and an acetonitrile‐deionized water mixture (70:30, v/v) as the mobile phase at a column temperature of 35 °C and UV absorption at 210 nm. The flow rate of 1 mL min^−1^ was used during the detection. 1,3‐DAP and ectoine were purchased from Sigma–Aldrich (USA) as 1,3‐DAP and ectoine standards, respectively.

### Violacein and SOD Assays

For total violacein assays, the extraction and quantification of violacein (including violacein and deoxyviolacein) was carried out using a modified method described in a previous study.^[^ ^48^ ^]^ First, 300 µL of fermentation broth, from either shake flask or fed‐batch study, was collected and centrifuged at 12 000 rpm for 5 min. Followed by 4‐ to 8‐fold dilution of the harvested supernatant, anhydrous ethanol was then 1:1 (vol) added and mixed to prepare samples for extracellular violacein analysis. Meanwhile, the cell pellet was resuspended using 600 µL of anhydrous ethanol for violacein extraction. The resultant cell suspension was sonicated (Supmile, KQ‐200VDE, China) for cell disruption, and subsequently centrifuged again at 12 000 rpm for 5 min to harvest the supernatant as a sample for intracellular violacein quantification. Finally, the concentration of violacein in total was measured by a Varioskan LUX microplate reader (570 nm, Thermo Scientific, VL0L00D0, USA).

For SOD analysis,^[^ ^47^ ^]^ 1 mL of cell culture was sampled and centrifuged at 12 000 rpm for 5 min at 4 °C to collect the cell pellet, which was then resuspended in PBS buffer for cell disruption using an ultrasonic homogenizer (JY92‐II N, SCIENTZ, China). The obtained homogenate was subsequently centrifuged again under the same conditions (12 000 rpm, 4 °C, 5 min) to obtain the supernatant containing soluble SOD for SDS‐PAGE analysis, which was performed using 4%–20% Bis‐Tris YoungPAGE gels (GenScript Biotech, M00928, USA). Meanwhile, the total soluble protein concentration of the supernatant was determined via a BCA protein assay kit (SmartBuffers, DFR‐0500, China). After electrophoresis, gel lanes were scanned by a flatbed scanner (EPSON, Perfection V39IIA4, Japan), and the mass ratio of SOD to total soluble proteins was calculated based on gel band intensity using Quantity One software (version 4.6.2, Bio‐Rad, USA). Finally, the SOD titer can be determined by the total protein concentration and the SOD/total protein ratio.

### Quantification and Statistical Analysis

Data were plotted and analyzed statistically using GraphPad Prism 10.0 (GraphPad Software, San Diego, CA). Two‐tailed unpaired Student's t‐test was used for statistical comparisons between two independent groups. One‐way analysis of variance (ANOVA) followed by post‐hoc Tukey's test was used for multiple comparisons. The n presents the number of biologically independent samples, as indicated in the figure captions. *p* < 0.05 was considered significant, and error bars represent standard error in all figures.

## Conflict of Interest

The authors declare no conflict of interest.

## Author Contributions

C.W., T.T.C., and Y.J.Y. contributed equally to this work. J.W.Y. conceived the idea and directed the project. L.X., Y.N.L., and J.W.Y. designed and supervised the experiments; C.W. performed strain modifications, adaptive laboratory evolution, constructed plasmids, and genome editing experiments. C.W., Y.X.L., and T.T.C. performed shake flash and fed‐batch fermentation. Y.J.Y.and Y.X.C. performed strain development and fermentation for formate utilization. Y.X.T. performed the electrochemical experiments. Y.C.X., W.T.G., Y.Z., R.Z.D., Y.X.T., W.S., H.W.S., Y.C., Y.B.W., J.X. and H.W. performed all other experiments; C.W., T.T.C., Y.X.T., L.X., Y.N.L. and J.W.Y. analyzed the data and wrote the manuscript. All authors discussed the results and approved the final manuscript.

## Supporting information



Supporting Information

Supplemental Data

## Data Availability

The data that support the findings of this study are available in the supplementary material of this article.
