# Supplementary material for: An Efficient CO2‐Upcycling Platform Based on Engineered Halomonas TD with Enhanced Acetate‐Utilizing Capacity via Adaptive Laboratory Evolution
Source: Adv Sci (Weinh). 2025 Oct 24;13(3):e13060. doi: 10.1002/advs.202513060 (PMC12806439; doi:10.1002/advs.202513060)
Supplement: Supplementary file 1 — Supporting Information [file ADVS-13-e13060-s001.docx]

Supplementary Information for

**An efficient CO_2_-upcycling platform based on engineered *Halomonas* TD with enhanced acetate-utilizing capacity via adaptive laboratory evolution**

**Authors:**

Chi Wang^1#^, Ting-Ting Chen^1#^, Yu-Jiao Yang^1#^, Yu-Xi Li^1^, Yi-Xin Chang^1^, Yan-Chun Xiao^4^, Wen-Tai Guo^9,10,11^, Ye Zheng^9,10,11^, Rui-Zhe Deng^9,10,11^, Yu-Xiang Tian^2,3^, Wei Situ^5^, Hong-Wei Shen^6^, Yu Chen^7^, Ya-Bin Wang^7^, Jie Xing^2^, Hui Wang^9,10,11^, Lin Xia^3,8*^, Yi-Na Lin^1*^, Jian-Wen Ye^1*^

**Affiliations:**

^1^ School of Biology and Biological Engineering, South China University of Technology, Guangzhou 510006, China

^2^ School of Science, China University of Geosciences, Beijing 100083, China

^3^ CAS Key Laboratory of Quantitative Engineering Biology, Shenzhen Institute of Synthetic Biology, Shenzhen Institutes of Advanced Technology, Chinese Academy of Science, Shenzhen 518055, China

^4^ Food and Drug Department, Liaoning Agricultural Vocational and Technical College, Yingkou, China

^5^ Guangzhou Medpha Biotechnology Co., Ltd., Guangzhou 510006, China

^6^ Guangdong Hefeng Biotechnology Co., Ltd., Zhanjiang, China

^7^ China Petroleum Engineering and Construction Corporation, 100120, China

^8^ Powered Carbon Biotechnology Co., Ltd., Shenzhen, 518055, China

^9^ Department of General Surgery (Colorectal Surgery), The Sixth Affiliated Hospital, Sun Yat-sen University, Guangzhou 510655, China

^10^ Guangdong Provincial Key Laboratory of Colorectal and Pelvic Floor Diseases, The Sixth Affiliated Hospital, Sun Yat-sen University, Guangzhou 510655, China

^11^ Biomedical Innovation Center, The Sixth Affiliated Hospital, Sun Yat-sen University, Guangzhou 510655, China

^#^Co-first authors contribute equally in this study: Chi Wang, Ting-Ting Chen, Yu-Jiao Yang.

*Corresponding author:

Lin Xia: lin.xia@siat.ac.cn

CAS Key Laboratory of Quantitative Engineering Biology, Shenzhen Institute of Synthetic Biology，Shenzhen Institutes of Advanced Technology, Chinese Academy of Science, Shenzhen 518055, China

Yi-Na Lin: linyn22@163.com (LIN YN)

School of Biology and Biological Engineering, South China University of Technology, Guangzhou 510006, China

Jian-Wen Ye: yejianwen@scut.edu.cn (YE JW);

School of Biology and Biological Engineering, South China University of Technology, Guangzhou 510006, China

Supplementary materials and methods

***Cathode catalyst preparation***

The Copper oxide (CuO) nanoparticles (Sigma Aladdin, less than 50 nm) were used as the cathodic catalyst for CO reduction reaction(CORR). 10 mg of copper oxide (CuO) was dispersed in 2 ml ethanol solution (25 vol%) and sonicated for 30 min. Followed by supplementing 100 µl Nafion (5 wt%), the obtained slurry was sprayed onto a carbon paper (JNTG20-A6L) using an ultrasonic spraying device (Shanghai Yonmi Intelligent Technology Co., Ltd.) with a loading capacity of 1 mg cm^-2^. The Ag nanoparticles (Sigma-Aladdin, less than 50 nm) and Bi_2_O_3_ the procedure was adjusted from previous protocol^1^, which were used as the cathodic catalyst for CO_2_ reduction reaction (CO_2_RR), were pretreated by the same method as CuO used, while the ethanol solution volume and ratio, as well as the loading capacity were changed to 2.9 mL, 10.71 vol%, and 1 mg cm^-2^, respectively.

***Measurement of electrochemical CO reduction reaction***

The prepared CuO and IrO_2_/Ti felts were used as cathode and anodefor the electrochemical CO reduction reaction to C_2+_ (eCORR), respectively. A layer of pre-treated FAA-3-50 membrane (fuma tech) was placed between the anode and cathode. The end plates on both sides of the cathode and anode use a domestic serpentine runner design.Then, 1 M KOH aqueous solution was used as the anode solution and circulated at a rate of 20 mL min^-1^ by using a peristaltic pump (KXP 100). The CO flow rate (40 sccm) was controlled on the cathode side by a mass flow controller (Sevenstar CS 200). The eCORR performance was evaluated by applying different voltages using an electrochemical workstation (Admiral Instruments). The gases generated during the electrocatalytic process were online detected by a gas chromatography (GC2060, Shanghai Ruimin Instrument Co., Ltd). The liquid product was measured using a Bruker 400 MHz liquid nuclear magnetic spectrometer (Bruker 400 MHz).

The Faraday efficiency (FE) of the gas and liquid products was calculated by Eq1:

$FE=\frac{m\times n\times F}{I\times t}$ (Eq1)

Where *m* is the mass of obtained liquid product (mol), *n* is the number of electron transfers. *F*  is the Faraday constant (C mol^-1^), *I* is the current (A) and *t* is the reaction time (s).

***Measurement of electrochemical CO_2_ reduction reaction***

The pretreated Ag and commercially purchased iridium oxide titanium felt anode was used as cathode and anode for the electrochemical CO_2_ reduction reaction to CO (eCO_2_RR), respectively. A pretreated PiperIon-A-40 membrane (Versogen) was placed between the anode and cathode. The end plates on both sides of the cathode are in line with the eCORR's. Then, 0.1 M KOH aqueous solution was used as the anode solution and circulated at a rate of 20 mL min^-1^ with a peristaltic pump (KXP 100). The CO_2_ flow rate (20 sccm) was controlled by a mass flow controller (Sevenstar CS 200) on the cathode side.

The pretreated Bi_2_O_3_ and commercially purchased iridium oxide titanium felts anode were used as cathode and anode for the electrochemical CO_2_ reduction reaction to formic acid (eCO_2_RR), respectively, with a pre-treated DuPont 117 membrane placed in the centre. The end plates on both sides of the cathode are in line with the eCORR's. Then 1 M KHCO_3_ aqueous solution was used as the electrolyte, which was circulated by a peristaltic pump at a rate of 15 mL min^-1^. The CO_2_ flow rate (40 sccm) was controlled by a mass flow controller on the cathode side. The performance of eCO_2_RR was evaluated by applying different cell voltages using an Admiral Instyments electrochemical workstation.

The gases generated during the electrocatalytic process were detected online by a gas chromatography (GC2060, Shanghai Ruimin Instrument Co., Ltd). The liquid products were measured using a Bruker 400 MHz liquid nuclear magnetic spectrometer (Bruker 400 MHz). The Faraday efficiency (FE) of a gas and liquid product were calculated by Eq1, respectively.

***Cascaded electrochemical reduction of CO_2_ and CO***

Due to the difficulty in achieving a one-step conversion of CO_2_ to C_2+_ by electrochemical carbon dioxide reduction at an industrial relevant scale and configeration, a two-step approach was taken in this experiment. Schematic diagram of the electrochemical reaction process is shown in Fig. 5a. Firstly, CO_2_ is supplied into the first electrolysis cell (Cell-I) for electrochemical reduction to obtain tail gas consisting of CO and CO_2_. Considering that most of the CO_2_ is still not involved in the reaction, the mixture is directly passed into the second electrolysis cell (Cell-II) to reduce the residual CO_2_ into formic acid only. Subsequently, the generated CO was passed into Cell-III for generating C_2+_ products mainly containing acetate. The formate and acetate were separated from KHCO₃ and KOH using a fully automatic universal distillation instrument (SQ-ZL600B). Finally, products from Cell-II and Cell-III would be mixed together as feed stock for downstream biofermentation.

Chronoamperometry (CA) at different current densities was performed during the electrochemical experiments of the reduction of CO_2_ to CO and their gas phase products were examined (Supplementary Fig. 17b). In the CO_2_RR to formic acid experiment, the CA at different current densities was performed, and liquid products were detected (Supplementary Fig. 17c). For the reduction of CO to C_2+_, the CA at different current densities was carried out and the resultant products were examined (Supplementary Fig. 17d).

**Supplementary results**

***Developing efficient electrochemical system for CO_2_-to-C_2+_ conversion.***

To generate a CDE solution rich in acetate for recombinant TD80 to use in this study, an efficient electrochemical system consisting of three cascaded reactions conducted in each individual electrolysis cell was developed to convert CO_2_ into C_2+_ electrolyte, mainly containing acetate, step-by-step (Supplementary Figs. 16 and 17a)^2^. Specifically, CO_2_ was initially introduced into the first electrolysis cell (Cell-I) for reducing CO_2_ into CO, namely eCO_2_RR-to-CO. However, the tail gas from Cell-I was generally a mixture of CO and residual CO_2_. Therefore, the tail gas was next transferred into the second reactor cell (Cell-II, eCO_2_RR-to-C_1+_) to partially convert unreacted CO_2_ into formic acid, aiming to obtain CO-enriched tail gas for effective electrochemical conversion towards C_2+_ in final step conducted in Cell-III, namely eCO_2_RR-to-C_2+_. Specifically, the components of gas phase product generated from Cell-I at different current densities, including 100, 200, 400 and 500 mA cm^-2^, were examined to assess the selectivity of CO, namely Faraday efficiency (FE), respectively. The highest FE value reached up to 99% at 500 mA cm^-2^ (Supplementary Fig. 17b). Besides, for electrochemical reduction of residual CO_2_ to formic acid in Cell-II, the selectivity of formic acid could maintain over 97.8% at a current density of 200 mA cm^-2^ (Supplementary Fig. 17c). In cell-III, Faraday efficiency of C_2+_ at different current densities was also studied, yielding 87.6% of C_2+_ selectivity at 200 mA cm^-2^ (the Faraday efficiency of acetate was 59.6%), of which the partial current density reached up to 175 mA cm^-2^ (Supplementary Fig. 17d). Since the reactions were carried out in different electrolytic cells under independently modulated environments, the concentration of formic acid (C_1+_ product) and acetate (C_2+_ product) can be detected separately by HPLC (Supplementary Fig. 18), respectively. Throughout the electrochemical system, the resultant electrolytes from Cell-II and Cell-III were recycled and mixed together, namely CDE, as feedstock solution for downstream microbial biosynthesis uses by recombinant TD80 when the mass ratio of acetate against formic acid reaching over 9:1(Supplementary Table 6).

Obviously, in contrast to the case of directly feeding tail gas from Cell-I to Cell-III^3^_,_ the introduction of Cell-II can effectively increase the CO content by 29.1%, reaching up to 25%, which enables higher selectivity of C_2+_ products in eCO_2_RR-to-C_2+。_Notably, no other products except formic acid were detected in electrolyte from Cell-II after five-times repeated studies. The liquid product of C_2+_ from Cell-III dominated by acetate and ethanol (acetic acid *vs* ethanol, 2.6:1) was heated during the electrochemical process for vaporizing ethanol to generate distilled ethanol and acetate-enriched electrolytes (Supplementary Fig. 17). The conversion rate of CO_2_ reached over 33%, which is significant improvement over previously reported studies^4, 5^. Besides, the tandem reactors can be run separately with different catalysts as well as reaction conditions, this will enable not only the precise control of each step, but also standard modularization and unitization for on-demand intermediate reactant input for subsequent chemical engineering or biofermentation process. Thus, the tandem reactor setup is promising for futural CO_2_ initiated power-to-X industry. In conclusion, considering industrial and commercial feasibility, this method not only improves the efficiency of the one-way conversion rate of CO_2_, but also provides an economically viable and favorable strategy for CO_2_ upcycling of reduced carbon footprint compared to the traditional alkali absorption-based separation methods that yield low value-added carbonates only^6, 7, 8, 9^.

Supplementary Tables

**Supplementary Table 1 Details of mutated genes in TD80 compared to the start host TD1.0.**

| **NCBI access No.** | **Descriptions** | **Gene name** | **Mutation sites** |
| --- | --- | --- | --- |
| WP_234667903.1 | RHS repeat-associated core domain-containing protein | TD01GL000644 | A213G/G215G/R215R/V242V/H243R |
| WP_009724971.1 | Rhomboid family intramembrane serine protease | TD01GL001360 | V120G |
| WP_234668036.1 | Pyruvate dehydrogenase **AceE** (acetyl-transferring) | TD01GL001382 | I601V, multiple mutations in 86-130 amino acids |
| WP_234667824.1 | Pyruvate dehydrogenase (acetyl-transferring, homodimeric type) | TD01GL003063 | /^a^ |
| [CAH1042028.1](https://www.ncbi.nlm.nih.gov/protein/CAH1042028.1?report=genbank&log$=protalign&blast_rank=1&RID=H20ZN3W0016) | Toxin protein Tse5 | TD01GL000647 | I25T/E32A/V35L/S187A/I188V/M189I/I193V/R218S/L224S/A225V/L274P/G309E/I451V/V461R/H481Q |
| [WP_267965106.1](https://www.ncbi.nlm.nih.gov/protein/WP_267965106.1?report=genbank&log$=protalign&blast_rank=1&RID=H22BNSJX01R) | Hemagglutinin repeat-containing protein | TD01GL001386 | N3084S/G3094K/A3096V/S3097T/V3100T/G3101A/V3102A/V3109I/D3114N/T3116A/T3123D/K3133N/R3134S |
| [WP_095602591.1](https://www.ncbi.nlm.nih.gov/protein/WP_095602591.1?report=genbank&log$=protalign&blast_rank=1&RID=H236GTE7016) | LysR^b^ substrate-binding domain-containing protein | TD01GL000842 | P120S |
| [WP_234667724.1](https://www.ncbi.nlm.nih.gov/protein/WP_234667724.1?report=genbank&log$=protalign&blast_rank=1&RID=H25PTZ5301R) | LapA^c^ adhesin domain-containing protein | TD00GL2614 | V1374A, code-shifting mutation at amino acid 1398 |
| [WP_234667724.1](https://www.ncbi.nlm.nih.gov/protein/WP_234667724.1?report=genbank&log$=protalign&blast_rank=1&RID=H27C3X87016) | LapA adhesin domain-containing protein | TD00GL2613 | V134A/D135*^d^ |
| [WP_234667724.1](https://www.ncbi.nlm.nih.gov/protein/WP_234667724.1?report=genbank&log$=protalign&blast_rank=1&RID=H27C3X87016) | LapA adhesin domain-containing protein | TD00GL2612 | 186 amino acid shift mutation |
| [WP_234667724.1](https://www.ncbi.nlm.nih.gov/protein/WP_234667724.1?report=genbank&log$=protalign&blast_rank=1&RID=H27C3X87016) | LapA adhesin domain-containing protein | TD00GL2611 | V348A/V562A, code-shifting mutation at amino acid 801 |
| [WP_234667724.1](https://www.ncbi.nlm.nih.gov/protein/WP_234667724.1?report=genbank&log$=protalign&blast_rank=1&RID=H27C3X87016) | LapA adhesin domain-containing protein | TD00GL2610 | code-shifting mutation at amino acid 159 |
| [WP_234667724.1](https://www.ncbi.nlm.nih.gov/protein/WP_234667724.1?report=genbank&log$=protalign&blast_rank=1&RID=H27C3X87016) | LapA adhesin domain-containing protein | TD00GL2609 | V134A/V348A/V562A, code-shifting mutation at amino acid 1015 |
| [WP_234667724.1](https://www.ncbi.nlm.nih.gov/protein/WP_234667724.1?report=genbank&log$=protalign&blast_rank=1&RID=H27C3X87016) | LapA adhesin domain-containing protein | TD00GL2608 | Code-shifting mutation at amino acid 134 |
| [WP_234667724.1](https://www.ncbi.nlm.nih.gov/protein/WP_234667724.1?report=genbank&log$=protalign&blast_rank=1&RID=H27C3X87016) | LapA adhesin domain-containing protein | TD00GL2607 | V134A, code-shifting mutation at amino acid 348 |
| [WP_234667724.1](https://www.ncbi.nlm.nih.gov/protein/WP_234667724.1?report=genbank&log$=protalign&blast_rank=1&RID=H27C3X87016) | LapA adhesin domain-containing protein | TD00GL2606 | V134A, code-shifting mutation at amino acid 343 |
| [WP_234667724.1](https://www.ncbi.nlm.nih.gov/protein/WP_234667724.1?report=genbank&log$=protalign&blast_rank=1&RID=H27C3X87016) | LapA adhesin domain-containing protein | TD00GL2602 | Code-shifting mutation at amino acid 134 |

^a^ Nonsense mutation at V489V, T499T;

^b^ LysR: a DNA-binding transcriptional regulator, LysR family;

^c^ LapA: Lipopolysaccharide assembly protein A;

^d^ Symbol of ‘*’ indicates stop codon amino acid;

**Supplementary Table 2 Strains used in this study.**

| **Strains** | **Descriptions** | **References** |
| --- | --- | --- |
| *Escherichia coli* S17-1 | A vector donor used for conjugation, harboring the *tra* genes from plasmid RP4 on the chromosome | Ref. ^10^ |
| *Halomonas* TD1.0  (TD1.0) | Recombinant derived from *Halomonas* TD01 with chromosomally integrated MmP1 RNA polymerase induced by IPTG | Ref. ^11^ |
| *Halomonas* TD80  (TD80) | ALE-evolved mutant of TD1.0 with strong acetate tolerance and utilization capability | This study |
| TD1.0-P | Derivate of TD1.0 by deleting *phaC* gene for PHA polymerization | This study |
| TD80-P | Derivate of TD80 by deleting *phaC* gene for PHA polymerization | This study |
| TD80-B | TD80P harboring plasmid containing P_porin281_-*phaA*-*phaB-tesB* expression module, Cm^R^ | This study |
| TD80-GB | TD80 harboring plasmid containing P_porin194_-*4hdb-sucD-ogdA* / P_porin194_*-orfZ* expression modules, Cm^R^ | This study |
| TD80-D | TD80 harboring plasmid containing P_porin58_-*aldD-dhaT* / P_porin194_*-orfZ* expression modules, Cm^R^ | This study |
| TD80-2 | TD80 with *doeA* deletion | This study |
| TD80-3 | TD80 with *ectD* deletion | This study |
| TD80-4 | TD80 with *doeA* and *ectD* deletion | This study |
| TD80-5 | TD80-4 with chromosomally integrated P_porin141_-*ectABC* module on G4 site | This study |
| TD80-6 | TD80-5 with chromosomally integrated P_porin226_-*lysC* module on G7 site | This study |
| TD80-7 | TD80-6 with chromosomally integrated P_porin58_-*asd* module on G43 site | This study |
| TD80-8 | TD80-7 harboring plasmid containing P_porin194_-*rocG* / P_porin58_*-aspC* expression modules, Cm^R^ | This study |
| TD80-9 | TD80-7 harboring plasmid containing P_porin68_-*pyc* / P_porin42_*-ppc* expression modules, Cm^R^ | This study |
| TD80-10 | TD80-7 harboring P_porin68_-*pyc-*P_porin42_*-ppc* module on pSEVA321 (Cm^R^) and P_porin194_-*rocG-*P_porin58_*-aspC* module on pSEVA341 (Sp^R^) | This study |
| TD80-DD | TD80 harboring P_porin194_-*dat* / P_porin194_*-ddc* on plasmid-carried system, Cm^R^ | This study |
| TD1.0-2 | TD1.0 with *doeA* deletion | This study |
| TD1.0-3 | TD1.0 with *ectD* deletion | This study |
| TD1.0-4 | TD1.0 with *doeA* and *ectD* deletion | This study |
| TD1.0-5 | TD1.0-4 with chromosomally integrated P_porin141_-*ectABC* module on G4 site | This study |
| TD1.0-6 | TD1.0-5 with chromosomally integrated P_porin226_-*lysC* module on G7 site | This study |
| TD1.0-7 | TD1.0-6 with chromosomally integrated P_porin58_-*asd* module on G43 site | This study |
| TD1.0-8 | TD1.0-7 harboring plasmid containing P_porin194_-*rocG* / P_porin58_*-aspC* expression modules, Cm^R^ | This study |
| TD1.0-9 | TD1.0-7 harboring plasmid containing P_porin68_-*pyc* / P_porin42_*-ppc* expression modules, Cm^R^ | This study |
| TD1.0-R | TD1.0 harboring plasmid containing P_porin58_-*rppA* expression module, Sp^R^ | This study |
| TD80-R | TD80 harboring plasmid containing P_porin58_-*rppA* expression module, Sp^R^ | This study |
| TD80-N | TD80 harboring plasmid containing P_Mmp1_-*nphT7* expression module, Cm^R^ | This study |
| TD80-8-N | TD80-8 harboring plasmid containing P_Mmp1_-*nphT7* expression module, Sp^R^ | This study |
| TD80-sfGFP | TD80 with chromosomally integrated Pporin140-*sfGFP* module on G4 site | This study |
| TD80-P1 | TD80 harboring plasmid containing P_Mmp1_-*ftl-folD* expression module, Cm^R^ | This study |
| TD80-P2 | TD80 harboring plasmid containing P_Mmp1_-*ftl*-P_Mmp1_-folD expression module, Cm^R^ | This study |
| TD80-C1 | TD80 with chromosomally integrated P_Mmp1_-*ftl*-P_Mmp1_-*folD* module on G4 site | This study |
| TD80-C1C2 | TD80-C1 harboring plasmid containing P_porin58_-*gcvH-gcvP* expression module, Cm^R^ | This study |

**Supplementary Table 3 Plasmids used in this study.**

| **Plasmids** | **Descriptions** | **References** |
| --- | --- | --- |
| pSEVA321 (p321) | p321 with RK2 replication origin, containing the *oriT* sequence for conjugation transformation, was used as an expression vector in *Halomonas* TD1.0 and its derivates in this study, Cm^R^. | This study |
| pSEVA341 (p341) | P341 with pRO1600 / ColE1 replicons, containing the *oriT* sequence for conjugation transformation, was used as an expression vector in *Halomonas* TD1.0 and its derivates in this study, Km^R^ and Sp^R^. | This study |
| p321-P_porinxxx_-*sfgfp*^a^ | p321 derivates harboring P_porin_ promoter mutants of different strength using sfGFP as reporter, details promoter sewuences were listed in Supplementary Table 5 | This study |
| p321-P_MmP1_-*sfgfp* | p321 derivate containing *sfgfp* expression module controlled by IPTG-induced P_MmP1_ | This study |
| p321-P_Lux_-*sfgfp* | p321 derivate containing *sfgfp* expression module controlled by P_Lux_ | This study |
| p321-P_MmP1_-*4hdb-sucD-ogdA-*P_porin194_-*orfZ* | p321 derivate containing *4hdb-sucD-ogdA* controlled by P_MmP1_ and *orfZ* controlled by P_porin194_ | This study |
| p321-P_porin58_-*4hdb-sucD-ogdA-*P_porin194_-*orfZ* | p321 derivate containing *4hdb-sucD-ogdA* controlled by P_porin58_ and *orfZ* controlled by P_porin194_ | This study |
| p321-P_porin58_-*aldD-dhaT-* P_porin194_-*orfZ* | p321 derivate containing *aldD-dhaT* and *orfZ* clusters controlled by P_porin58_ and P_porin194_, respectively | This study |
| p321-P_MmP1_-*phaA*-*phaB-tesB* | p321 derivate containing *phaA*-*phaB-tesB* cluster controlled by P_MmP1_ | This study |
| p321-P_porin281_-*phaA*-*phaB-tesB* | p321 derivate containing *phaA*-*phaB-tesB* cluster controlled by P_porin281_ | This study |
| p321-P_porin141_-*ectABC* | p321 derivate containing *ectABC* cluster controlled by P_porin141_ | This study |
| p321-P_porin226_-*lysC* | p321 derivate containing *lysC* cluster controlled by P_porin226_ | This study |
| p321-P_porin58_-*asd* | p321 derivate containing *asd* cluster controlled by P_porin_ | This study |
| p341-P_porin140_-*ectABC* | p341 derivative containing P_J23119_-sgRNA (IR 00227-00228, G4)::P_porin140_-*ectABC*, 1 kp donor, Km^R^ and Sp^R^ | This study |
| p341-P_porin226_-*lysC* | p341 derivative containing P_J23119_-sgRNA (IR 02654-02655, G7)::P_porin226_-*lysC*, 1 kp donor, Km^R^ and Sp^R^ | This study |
| p341-P_porin58_-*asd* | p341 derivative containing P_J23119_-sgRNA (IR 00653-00654, G43)::P_porin58_-*asd*, 1 kp donor, Km^R^ and Sp^R^ | This study |
| p321-P_MmP1_-*rocG*-P_Lux_-*aspC* | p321 derivate containing *rocG* and *aspC* controlled by P_MmP1_ and P_Lux_, repsectively | This study |
| p321-P_porin194_-*rocG*-P_porin58_-*aspC* | p321 derivate containing *rocG* controlled by P_porin194_ and *aspC* controlled by P_porin58_ | This study |
| p321-P_MmP1_-*ppc*-P_Lux_-*pyc* | p321 derivate containing *ppc* controlled by P_MmP1_ and *pyc* controlled by P_Lux_ | This study |
| p321-P_porin68_-*ppc*-P_porin42_-*ppc* | p321 derivate containing *ppc* controlled by P_porin68_ and *pyc* controlled by P_porin42_ | This study |
| p321-P_MmP1_-*dat-ddc* | p321 derivate containing *dat-ddc* cluster controlled by P_MmP1_ | This study |
| p321-P_porin194_-*dat-*P_porin194_-*ddc* | p321 derivate containng *dat* and *ddc* modules controlled by P_porin194_ and P_porin194_, respectively | This study |
| p341-P_porin58_-*rppA* | p321 derivate containing *rppA* controlled by P_porin58_ | This study |
| p321-P_MmP1_-nphT7 | p321 derivate containing *nphT7* controlled by P_MmP1_ | This study |
| p341-P_MmP1_-nphT7 | p341 derivate containing *nphT7* controlled by P_MmP1_ | This study |
| p321-P_MmP1_-*ftl-folD* | p321 derivate containing *ftl-folD* controlled by P_MmP1_ | This study |
| p321-P_MmP1_-*ftl-* P_MmP1_-*folD* | p321 derivate containing *ftl-folD* controlled by dual P_MmP1_ | This study |
| p321-P_MmP1_-*vioABCDE* | p321 derivate containing *vioABCDE* controlled by P_MmP1_ | Ref.^12^ |
| p321-P_MmP1_-*sod* | p321 derivate containing *sod* controlled by P_MmP1_ | Ref.^13^ |

^a^ ‘XXX’ with bottom line indicates promoter No. of P_porin_ promoter.

**Supplementary Table 4 Genes used in this study**

| **Genes** | **Descriptions** | **References** |
| --- | --- | --- |
| *4hbD* | Encoding 4-hydroxybutyrate dehydrogenase | This study |
| *sucD* | Encoding succinate semialdehyde dehydrogenase | This study |
| *ogdA* | Encoding 2-oxoglutarate decarboxylase | This study |
| *orfZ* | Encoding CoA transferase | This study |
| *phaA* | Encoding 3-ketothiolase | This study |
| *phaB* | Encoding acetoacetyl-CoA reductase | This study |
| *tesB* | Encoding thioesterase | This study |
| *dhaT* | Encoding 1, 4-butanediol reductase | This study |
| *aldD* | Encoding aldehyde dehydrogenase | This study |
| *dat* | Encoding 2-ketoglutarate 4-aminotransferase | This study |
| *ddc* | Encoding L-2,4-diaminobutanoate decarboxylase | This study |
| *ectA* | Encoding L-2,4-diaminobutyrate acetyltransferase | This study |
| *ectB* | Encoding L-2,4-diaminobutyrate transaminase | This study |
| *ectC* | Encoding ectoine synthase | This study |
| *doeA* | Encoding ectoine hydrolase | This study |
| *ectD* | Encoding ectoine hydroxylase | This study |
| *lysC* | Encoding aspartokinase | This study |
| *asd* | Encoding L-aspartate-semialdehyde-dehydrogenase | This study |
| *rocG* | Encoding glutamate dehydrogenase | This study |
| *aspC* | Encoding aspartate aminotransferase | This study |
| *pyc* | Encoding pyruvate carboxylase | This study |
| *ppc* | Encoding phosphoenolpyruvate carboxylase | This study |
| *rppA* | Encoding 1,3,6,8-tetrahydroxynaphthalene synthase | This study |
| *nphT7* | Encoding acetoacetyl-CoA synthase | This study |
| *vioABCDE* | Encoding tryptophan oxidase, indole-3-pyruvate, decarboxylase, oxidoreductase, cyclase and oxygenase | Ref.^12^ |
| *sod* | Encoding superoxide dismutase | Ref.^13^ |
| *ftl* | Formate-THF ligase | This study |
| *folD* | Methylenetetrahydrofolate dehydrogenase/methenyltetrahydrofolate cyclohy-drolase | This study |
| *fch* | ormyl-THF cycloligase | This study |
| *mtdA* | methylene-THF dehydrogenase | This study |

**Supplementary Table 5 Sequences of P_porin_ promoters used in this study.**

| **Genes** | **Descriptions^a^** | **References** |
| --- | --- | --- |
| P_porinWT_ | TTGCGTTCACTGGAATCCCAGTATAGAGTTTGACCTGCGAGCA | Ref. ^14^ |
| P_porin281_ | TTGCGTTCACTGGAATCCCAGTATTACCTTTGACCTGCGAGCA | Ref. ^14^ |
| P_porin194_ | TTGCGTTCACTGGAATCCCAGTATCTAATTTGACCTGCGAGCA | Ref. ^14^ |
| P_porin58_ | TTGCGTTCACTGGAATCCCAATATAGAGTTTGACCTGCGAGCA | Ref. ^14^ |
| P_porin226_ | TTGCGTTCACTGGAATCCCAGTATAAAGTTTGACCTGCGAGCA | Ref. ^14^ |
| P_porin140_ | TTGCGTTCACTGGAATCCCAGTATAAGATTTGACCTGCGAGCA | Ref. ^14^ |
| P_porin68_ | TTGCGTTCACTGGAATCCCAGACTAGAGTTTGACCTGCGAGCA | Ref. ^14^ |

^a^ letters with bottom line indicate mutation site of P_porin_ mutants compared to the wild type one (P_porinWT_).

**Supplementary Table 6** **Components of CDE solutions with max- and min-acetate concentration.**

|  | Acetate (g L^-1^) | Formate (g L^-1^) | Bicarbonate (g L^-1^) |
| --- | --- | --- | --- |
| Min-acetate | 60 | 20 | 80 |
| Max-acetate | 90 | 10 | 0.8 |

**Supplementary Table 7 Production titers of different products from max- and min-acetate CDE, respectively.**

|  | PHB  (g L^-1^) | P34HB  (g L^-1^) | 3HB  (g L^-1^) | 1,3-DAP  (g L^-1^) | Ectoine (g L^-1^) |
| --- | --- | --- | --- | --- | --- |
| Min-acetate | 5.48 | 3.13,  6.54 mol% 4HB | 4.07 | 0.56 | 3.38 |
| Max-acetate | 7.72 | 5.50,  8.36 mol% 4HB | 6.93 | 0.59 | 5.59 |
| 50MMA | 7.55 | 5.36,  8.29 mol% 4HB | 6.62 | 0.64 | 5.89 |

**Supplementary Table 8 Comparison of production yields in this study against previous studies.**

| Conversion design from CO_2_ to product | Host | Titer  (g L^-1^) | Productivity  (g L^-1^ h^-1^) | Carbon conversion rate (mol%) | References |
| --- | --- | --- | --- | --- | --- |
| CO_2_-to-acetate-to-glucose | *Saccharomyces cerevisiae* | / | / | / | Ref. ^15^ |
| CO_2_-to-acetate-to-food^b^ | *Saccharomyces cerevisiae* | / | / | / | Ref. ^5^ |
| Ethaol-to-glucose | *Saccharomyces cerevisiae* | 18.28 | 0.078 | 6.9 | Ref. ^16^ |
| Ethaol-to-sucrose | *Saccharomyces cerevisiae* | 24.15 | 0.093 | 6.8 | Ref. ^16^ |
| Methaol-to-glucose | *pichia pastoris* | 13.41 | 0.047 | 8.5 | Ref. ^16^ |
| CO_2_-to-formate-to-PHB | *Cupriavidus necator* | 1.38 | 0.012 | / | Ref. ^17^ |
| CO_2_-to-formate-to-PHB | *Cupriavidus necator* | / | 0.017 | 5.2 | Ref. ^18^ |
| CO_2_-to-formate-to-PHB | *Cupriavidus necator* | 0.056 | 0.002 | / | Ref. ^19^ |
| CO_2_-to-C_2+_-to-PHA | *Pseudomonas putida* | 0.56 | 0.023 | / | Ref. ^20^ |
| CO_2_-to-acetate | *Sporomusa ovata* | / | / | / | Ref. ^21^ |
| Acetate-to-PHB | *Halomonas* TD | 49.78 | 0.332 | 33.5 | Ref.^22^ |
| CO_2_-to-PHB | *Ralstonia eutropha* | / | / | / | Ref. ^23^ |
| CO_2_-to-formate-to-protein | *Paracoccus communis* MA5 | 2.60 | 0.026 | / | Ref. ^24^ |
| Flue gas(CO_2_)-to-PHB | *Synechococcus elongatus* UTEX 2973 | 0.22 | 0.003 | / | Ref. ^25^ |
| Glucose-to-ectoine | *Halomonas* TD | 28 | 0.99 | 26.6 | Ref.^26^ |
| Glucose-to-PHB & ectoine | *Halomonas* TD | (24.0_PHB_ + 8.0_ectoine_) | 0.73 (0.55_PHB_ + 0.18_ectoine_) | / | Ref.^26^ |
| Glucose-to-ectoine | *Halomonas* TD | 84.6 | 1.63 | 36.7 | Ref.^27^ |
| Acetate-to-PHB | TD80 | 37.9 | 0.8 | 29.8 | This study^a^ |
| Acetate-to-PHB & ectoine | TD80 | (17.6_PHB_ + 23.9_ectoine_) | 1.2 (0.5_PHB_ + 0.7_ectoine_) | 38.3 | This study^b^ |
| CO_2_-to-CDE-to-PHB | TD80 | 34.3 | 0.7 | 39.3 | This study^c^ |
| CO_2_-to-CDE-to-ectoine | TD80 | 28.4 | 0.9 | 39.7 | This study^d^ |
| CO_2_-to-CDE-to-ectoine & PHB | TD80 | (28.9_ectoine_ + 7.5_PHB_) | 1.0 (0.8_ectoine_ + 0.2_PHB_) | 53.7 | This study^e^ |

/, Not mentioned in the references

^a and b^ Figs. 4g and 4h in this study

^c, d and e^ Figs. 5i,5j, and 5k in this study

**Supplementary Table 9 Different products of high-level carbon storage capacity.**

|  | Glucose^15,16^ | Starch^18^ | Sucrose^16^ | PHB/P34HB^a^ | 3HB^a^ | 1,3-DAP^a^ | Ectoine^a^ |
| --- | --- | --- | --- | --- | --- | --- | --- |
| g_CO2_/g_product_ | 1.5 | 1.6 | 1.5 | 2.1 | 1.7 | 1.8 | 1.9 |

^a^ Figs. 2-4 in this study

Supplementary figures

**
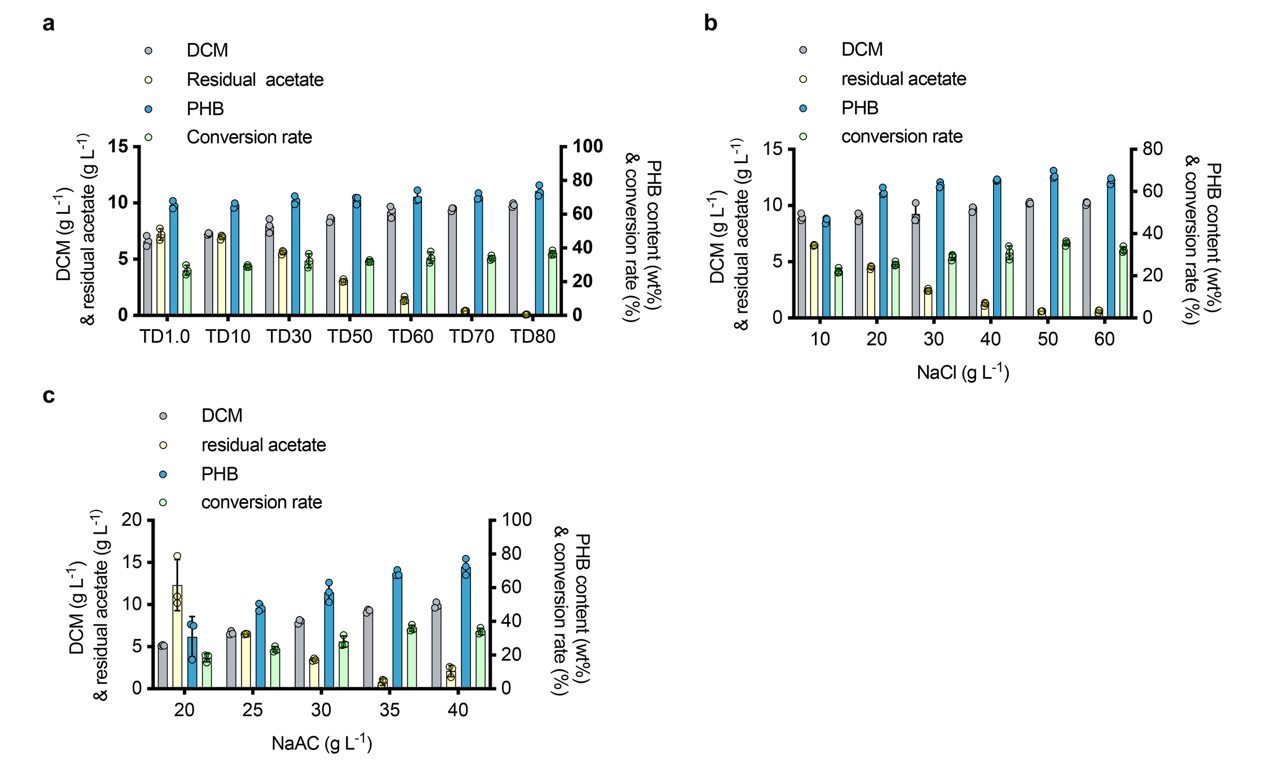
**

**Supplementary Fig. 1 Medium optimization for TD80.**

**a** Shake flask studies of DCM, PHB accumulation, residual acetate and conversion rate of acetate-to-PHB by different ALE-mutant strains from TD10 to TD80. **b** and **c** Shake flask studies of CDW, PHB accumulation, residual acetate and conversion rate of acetate-to-PHB by TD80 grown in MMA medium supplemented with different concentration of NaCl (**b**, 10-60 g L^-1^) and acetate (**c**, 20-40 g L^-1^), respectively. Error bars represent standard deviations, n = 3

**
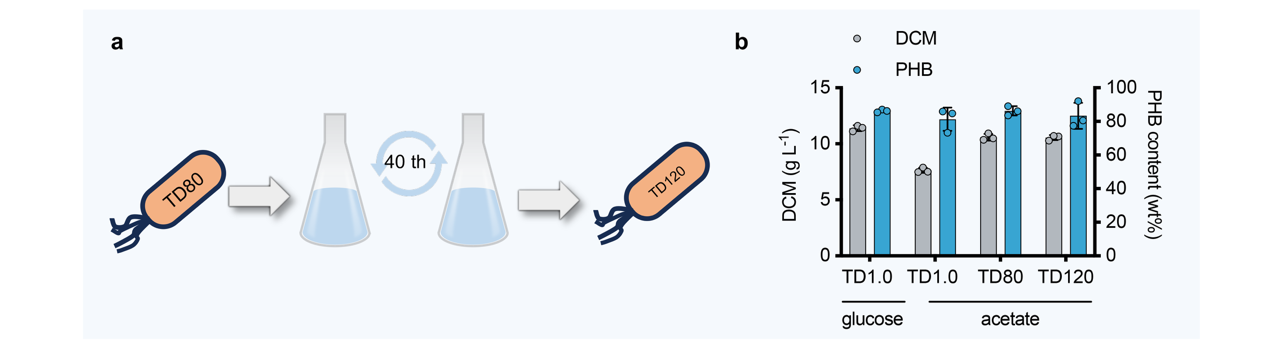
**

**Supplementary Fig. 2 Adaptive laboratory evolution of *Halomonas* TD80**

**a** Schematic design of liquid-state culture-based ALE of TD80 grown in 500-mL shake flask in 50MMA medium supplemented with 100-to-120 g L^-1^ acetate. Seed culture was initially inoculated (2 vol%) into 50 mL 50 MMA medium (supplemented with 100 g L^-1^ acetate) and grown for 24 h (37 °C and 200 rpm). Followed by repeated passaging until the cell density (OD_600_) stop increasing, meanwhile, the acetate supplementation in 50MMA medium was increased 10 g L^-1^ per time for enhanced evolutionary pressure till reaching up to 120 g L^-1^. Finally, the resultant ALE-strain TD120 after 40-generation passaging growth in 50MMA medium containing up to 120 g L^-1^ acetate was obtain for further comparative assessment. **b** Shake flask studies of DCM and PHB content by two evolved strains, namely TD80 (solid-state culture-based ALE from TD1.0) and TD120 (liquid-state culture-based ALE from TD80) compared to start host TD1.0 grown in 50MMA medium. In contrast, *Halomonas* TD1.0 grown in 50MMA (supplemented with 35 g L^-1^ acetate) and 50MMG media, respectively, were used as control groups. Error bars represent standard deviations, n = 3.


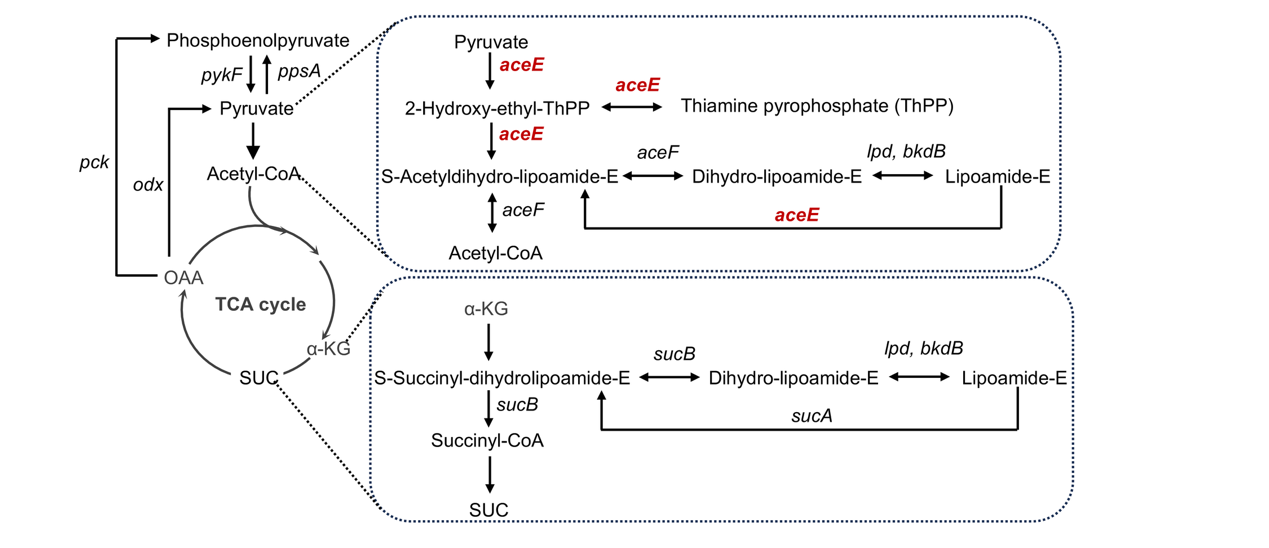


**Supplementary Fig. 3 Metabolic pathways related to ALE-mutatation gene *aceE*.**

Metbolic pathways encoded by genes (italic letter) with close interaction with ALE-mutation gene *aceE* regenerated from STRING (see Fig. 1f). Genes: *aceE*, pyruvate dehydrogenase (acetyl transfer); *aceF*, pyruvate dehydrogenase e2 component; *lpd3*, dihydrolipoamide dehydrogenase; *lpdG*, dihydrolipoamide dehydrogenase; *lpdV*, dihydrolipoamide dehydrogenase; *bkdB*, branched-chain alpha-keto acid decarboxylase; *ppsA*, phosphoenolpyruvate synthase; *pykF*, pyruvate kinase F; *sucB*, dihydrolipoyllysine-residue succinyltransferase.


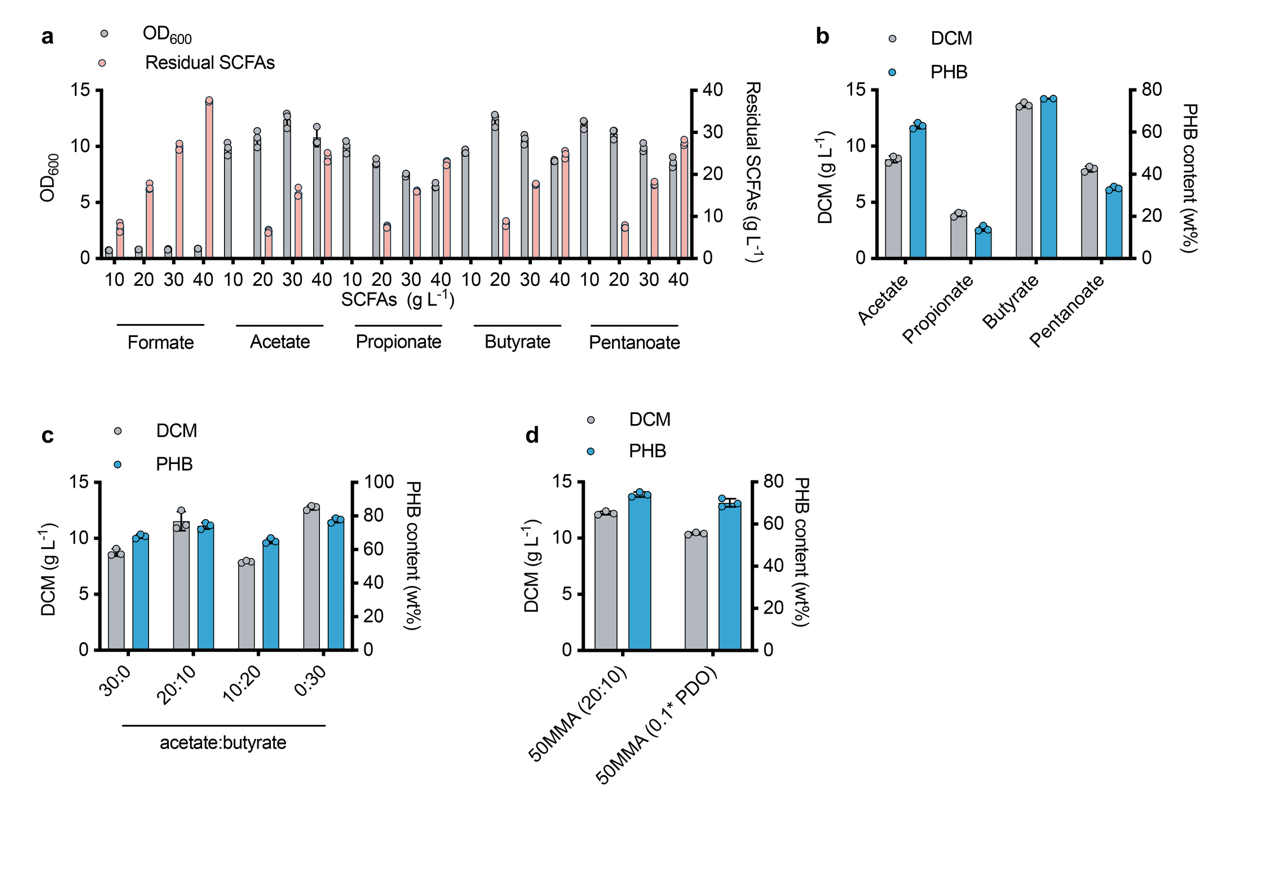


# Supplementary Fig. 4 Assessment of short-chain fatty acids (SFCAs) utilization by TD80.

**a** 96-Deep-well plate study of cell growth (OD_600_) by TD80 grown in 50MMA medium supplemented with different SFCAs (C_1_-C_5_, 10-40 g L^-1^) for 24 h. **b** Shake flask study of DCM and PHB content by TD80 grown in 50MM medium supplemented with different SFCAs (C_2_-C_5_, 30 g L^-1^). **c** Shake flask study of DCM and PHB content by TD80 grown in 50MM medium supplemented with different mixed ratios of acetate and butyrate. **d** Shake flask study of DCM and PHB content by TD80 grown in 50MM medium supplemented with mixed carbon source (20 g L^-1^ acetate & 10 g L^-1^ butyrate, and PDO-derived waste solution (10 vol% addition), which contains different SCFAs including formic acid (8.6 g L^-1^), acetic acid (249 g L^-1^), butyric acid (118.8 g L^-1^), lactic acid (5.68 g L^-1^), ethanol (2.4 g L^-1^), glycerinum (15.9 g L^-1^) from Guangdong Hengtan Technology Co., Ltd. (https://www.c1lifebio.com/). PDO, 1,3-propanediol. Error bars represent standard deviations, n = 3.


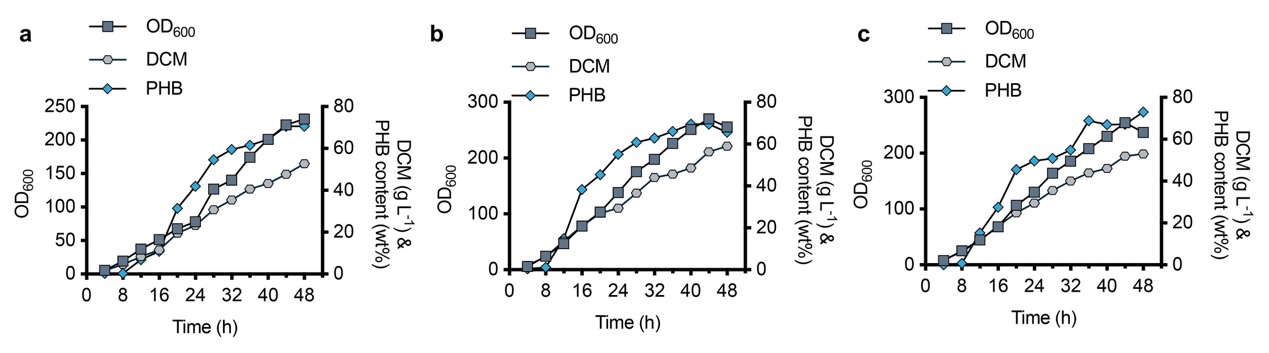


**Supplementary Fig. 5 Triplicate fed-batch studies of PHB production by TD80.**

**a**, **b** and **c** Three repeated fed-batch studies of PHB production by TD80 grown in a 7-L bioreactor feeded with acetate as sole carbon source (see Methods). DCM, OD_600_ and PHB content were recorded in every 4 h throughout the 52-h open fermentation process.


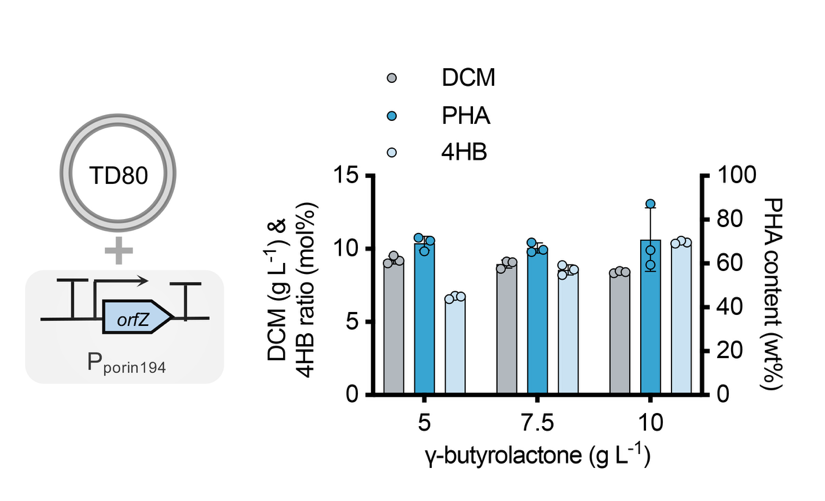


**Supplementary Fig. 6 Engineering TD80 for effective production of P34HB by adding** **γ-butyrolactone.**

DCM and PHA content by recombinant TD80 harboring P_porin194_-*orfZ* expression module grown in a 500-mL shake flask in 50MMA medium supplemented with 5, 7.5 and 10 g L^-1^ γ-butyrolactone (GBL) used as a precursor for 4HB monomer accumulation in P34HB. Error bars represent standard deviations, n = 3.


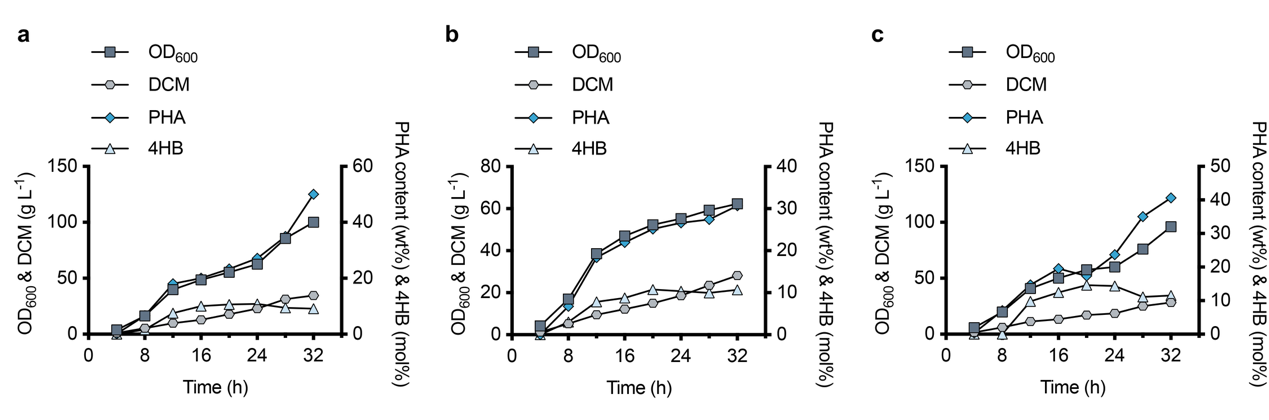


**Supplementary Fig. 7 Triplicate fed-batch studies of P34HB production by TD80 from acetate.**

**a**, **b** and **c** Three repeated fed-batch studies of P34HB production by recombinant TD80 grown in a 7-L bioreactor feeded with acetate as sole carbon source (see Methods). DCM, OD_600_, PHA content and 4HB molar ratio were recorded in every 4 h throughout the 32-h open fermentation process.


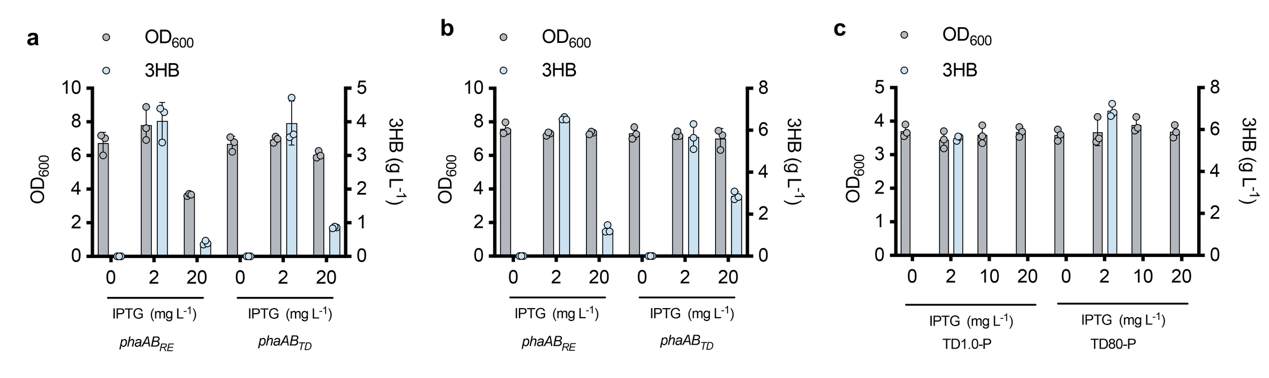


**Supplementary Fig. 8 De novo synthesis of 3HB by recombinant TD80.**

**a** OD_600_ and 3HB accumulation by TD1.0-P harboring *phaAB_RE/TD_-tesB* expression module controlled by P_MmP1_ promoter grown in 50MMG medium (30 g L^-1^ glucose) in the presence of 0, 2 and 20 mg L^-1^ IPTG, respectively. **b** OD_600_ and 3HB accumulation by TD80-P harboring *phaAB_RE/TD_-tesB* expression module controlled by P_MmP1_ promoter grown in 50MMA medium (35 g L^-1^ acetate) in the presence of 0, 2 and 20 mg L^-1^ IPTG, respectively. **c** Comparative analysis of OD_600_ and 3HB accumulation by two *phaC*-defected strains, namely TD1.0-P and TD80-P, harboring *phaAB_RE_-tesB* controlled by P_MmP1_ promoter grown in 50MMA medium (35 g L^-1^ acetate) in the presence of 0, 2 and 20 mg L^-1^ IPTG, respectively. Error bars represent standard deviations, n = 3.


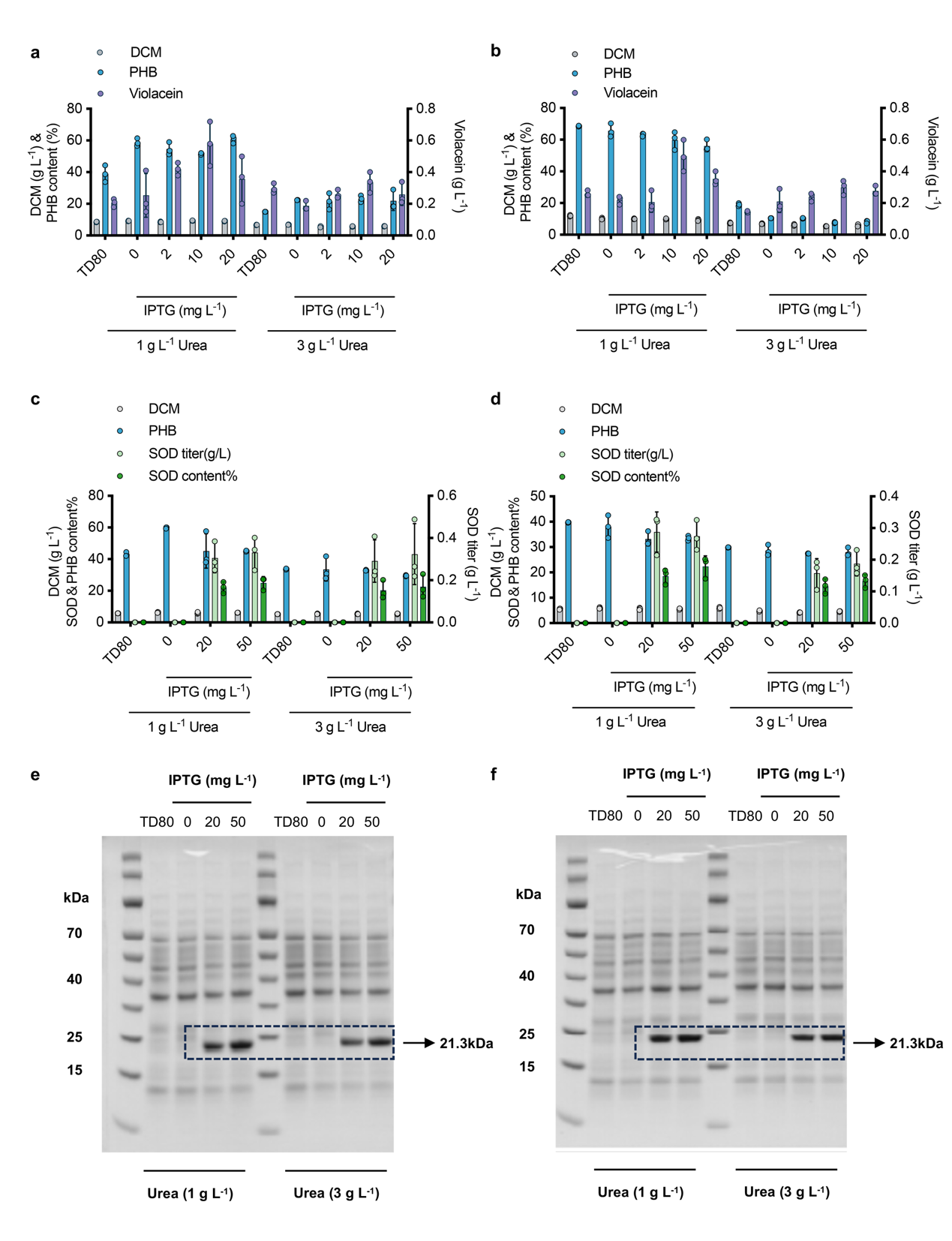


**Supplementary Fig. 9 Biosynthesis of violacein and SOD by recombinant TD80 from acetate and industrial waste SCFAs.**

**a-b** Shake flask study of violacein, DCM and PHB content by recombinant TD80 harboring P_MmP1_-*vioABCDE* expression module grown in 50MMAU (**a**) (35 g L^-1^ acetate, 1 or 3 g L^-1^ urea) and 50MMPU (**b**) (10 vol% PDO-derived waste solution, 1 or 3 g L^-1^ urea) media in the presence of different IPTG concentrations. **c-d** Shake flask study of SOD, DCM and PHB content by recombinant TD80 harboring P_MmP1_-*sod* module induced by IPTG (0, 20 and 50 mg L^-1^) grown in 50MMA (**c**) and 50MMPU (**d**), respectively. **e-f** SDS-PAGE analysis of SOD by engineered TD80 grown in 50MMAU (**e**) and 50MMPU (**f**), respectively, in the presence of 50 mg L^-1^ IPTG. Error bars represent standard deviations, n = 3.


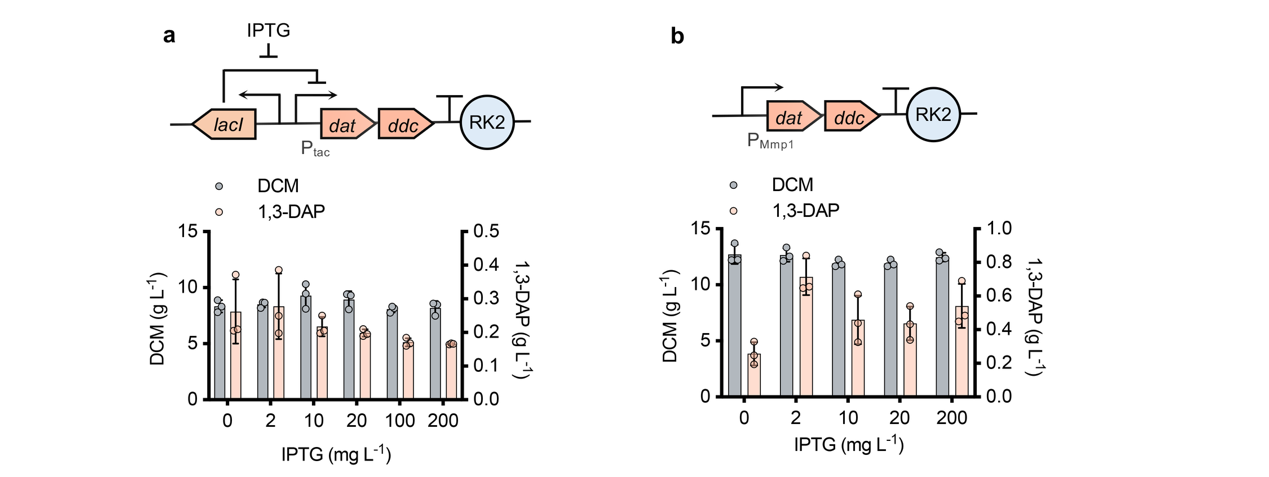


**Supplementary Fig. 10** **De novo synthesis of 1,3-DAP by recombinant TD80 from acetate.**

**a** and **b** Expression tuning of 1,3-DAP synthesis module encoded by *dat-ddc* controlled by P_tac_ (**a**) and P_MmP1_ (**b**) prmoter with small and large tuning scope, respectively. DCM and 1,3-DAP titer by recombinant TD80 grown in 50MMA medium (supplemented with 3 g L^-1^ urea) were showed. Error bars represent standard deviations, n = 3.


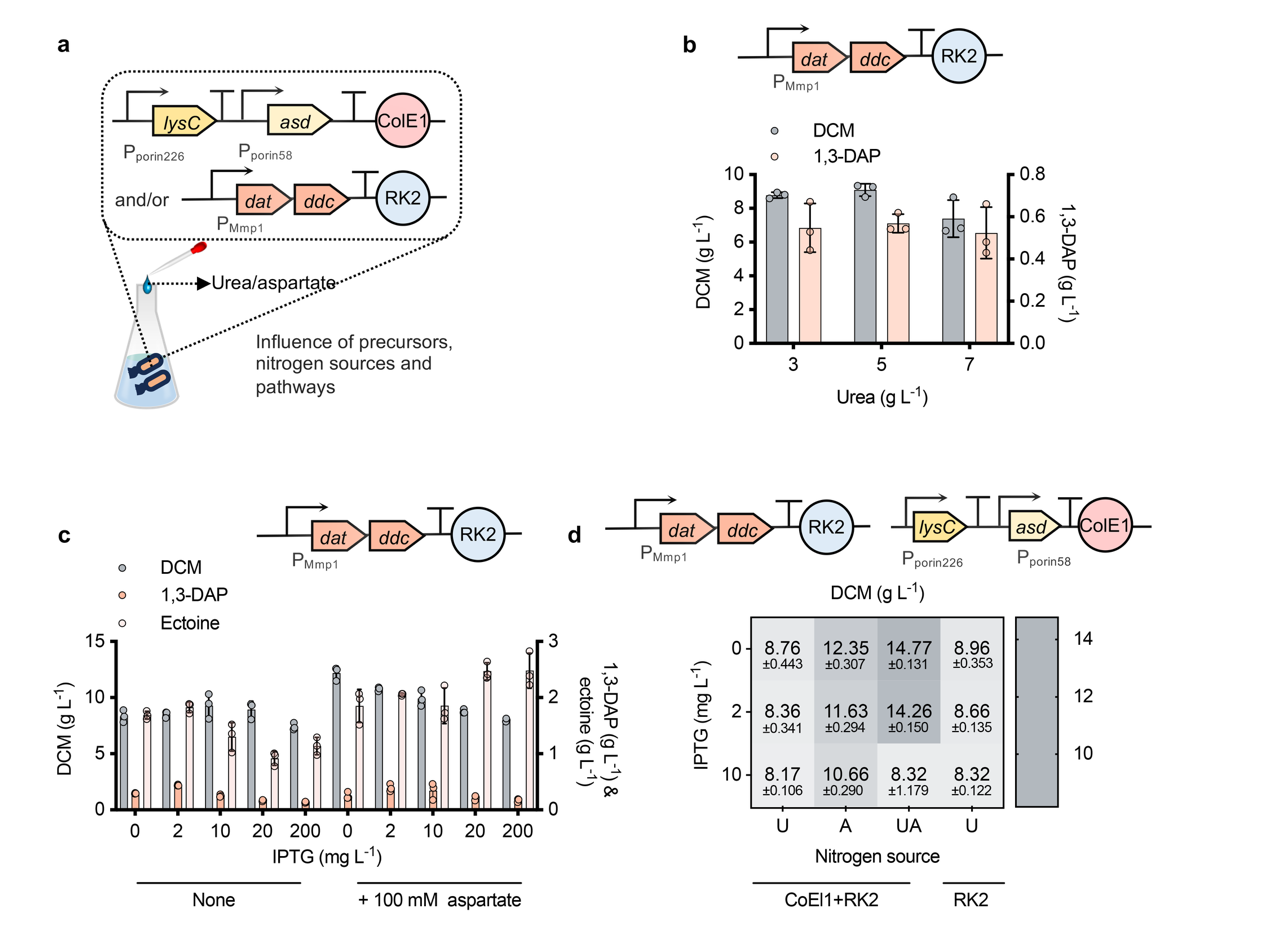


**Supplementary Fig. 11 Studying the effects on 1,3-DAP synthesis by reconmbiant TD80 from acetate.**

**a** Studying the effects on 1,3-DAP synthesis by enhancing the supply of precursor (aspartate), nitrogen source (urea), and upstream pathway flux (overexpressing *lysC* and *asd* genes). **b** DCM and 1,3-DAP accumulation by recombinant TD80 harboring harboring *dat-ddc* expression module controlled by P_MmP1_ promoter grown in 50MMA medium (35 g L^-1^ acetate) supplemented with 3, 5 and 10 g L^-1^ urea induced by 2 mg L^-1^ IPTG. **c** DCM, 1,3-DAP, and ectoine titer by recombinant TD80 harboring *dat-ddc* module from part **a** grown in 50MMA medium (3 g L^-1^ urea) with (+) and without 100 mM aspartate addition. **d** Shake flask studies of DCM and 1,3-DAP (see Fig. 3d) by recombinant TD80 grown on acetate. U, A, and UA represent 50MMA medium supplemented with 3 g L^-1^ urea, 100 mM aspartate, and their combination, respectively. RK2 and CoEl represent expression vector pSEVA321 and pSEVA341, respectively. Error bars represent standard deviations, n = 3.


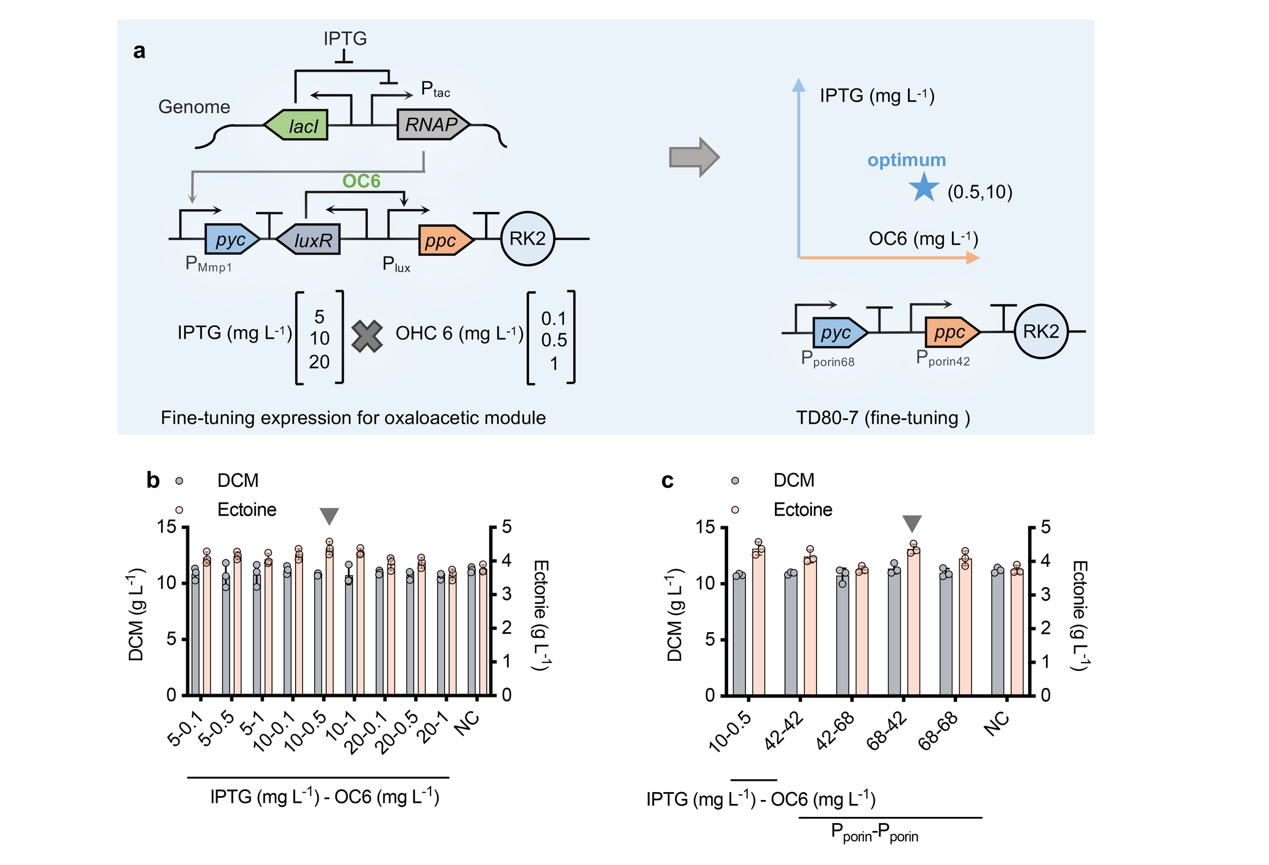


**Supplementary Fig. 12 Combinatory expression tuning of *pyc* and *ppc* genes by two orthogonal inducible systems.**

**a** schematic tuning design of *pyc* and *ppc* genes driven by two orthogonal inducible systems, namely P_MmP1_ induced by IPTG and P_lux_ by OC6, respectively. Constitutive promoter replacement was next determined directed by the induction levels of IPTG and OC6. **b** DCM and ectoine accumulation by recombinant TD80 harboring P_140_-*ectABC* (integrated on G4 loci), and P_MmP1_-*pyc* (pSEVA321) and P_lux_-*ppc* (pSEVA321) modules in 50MMA (3 g L^-1^ Urea) medium in a 500-mL shake flask. **c** DCM and ectoine accumulation by recombinant TD80 based on promoter replacement of *pyc* and *ppc* genes directed by the induction levels of IPTG and OC6 from part **b** compared to the optimal group from part **b** induced by 10 mg L^-1^ IPTG and 0.5 mg L^-1^ OC6. Error bars represent standard deviations, n = 3.

**
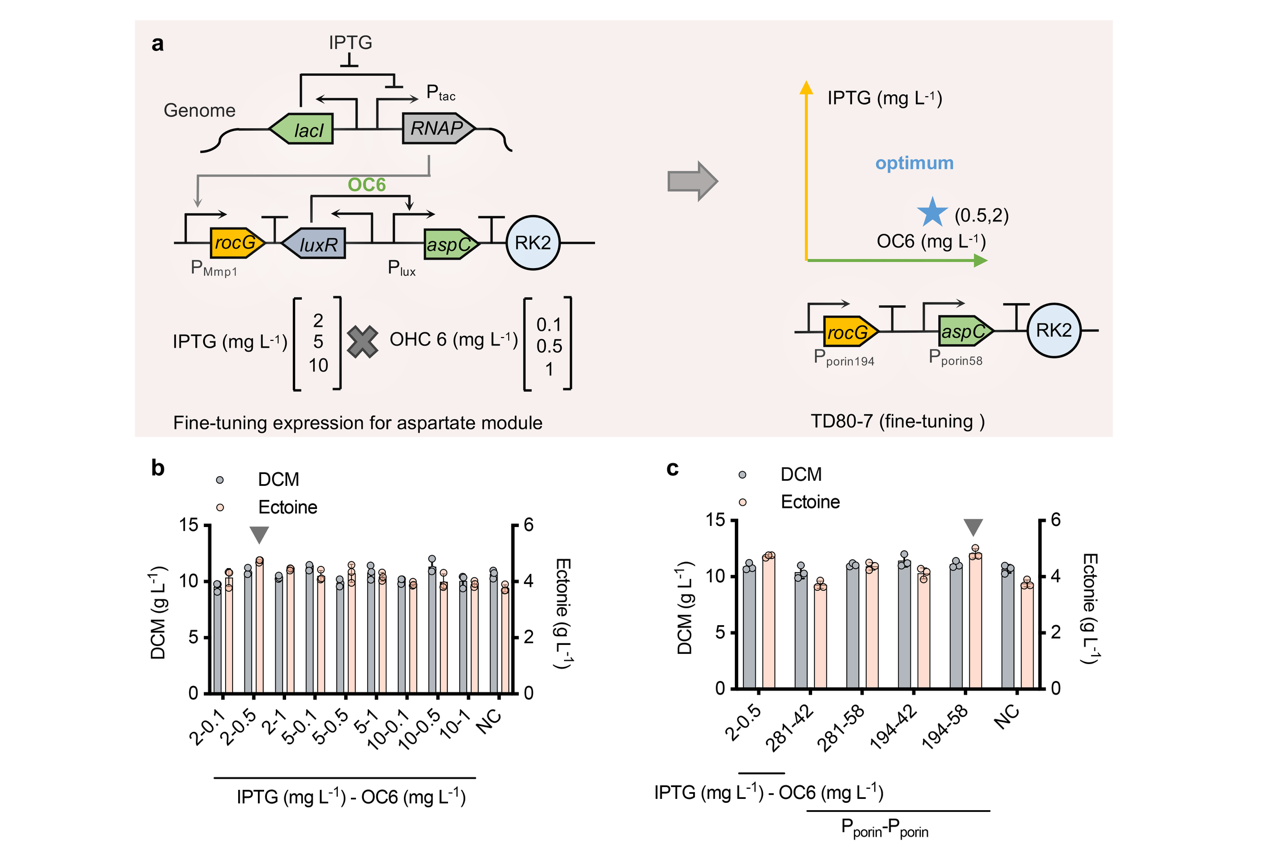
**

**Supplementary Fig. 13 Combinatory expression tuning of *rocG* and *aspC* genes by two orthogonal inducible systems.**

**a** schematic tuning design of *rocG* and *aspC* genes driven by two orthogonal inducible systems, namely P_MmP1_ induced by IPTG and P_lux_ by OC6, respectively. Constitutive promoter replacement was next determined directed by the induction levels of IPTG and OC6. **b** DCM and ectoine accumulation by recombinant TD80 harboring P_140_-*ectABC* (integrated on G4 loci), P_MmP1_-*rocG* (pSEVA321) and P_lux_-*aspC* (pSEVA321) modules in 50MMA (3 g L^-1^ Urea) medium in a 500-mL shake flask. **c** DCM and ectoine accumulation by recombinant TD80 based on promoter replacement of *rocG* and *aspC* genes directed by the induction levels of IPTG and OC6 from part **b** compared to the optimal group from part **b** induced by 2 mg L^-1^ IPTG and 0.5 mg L^-1^ OC6. Error bars represent standard deviations, n = 3.

**
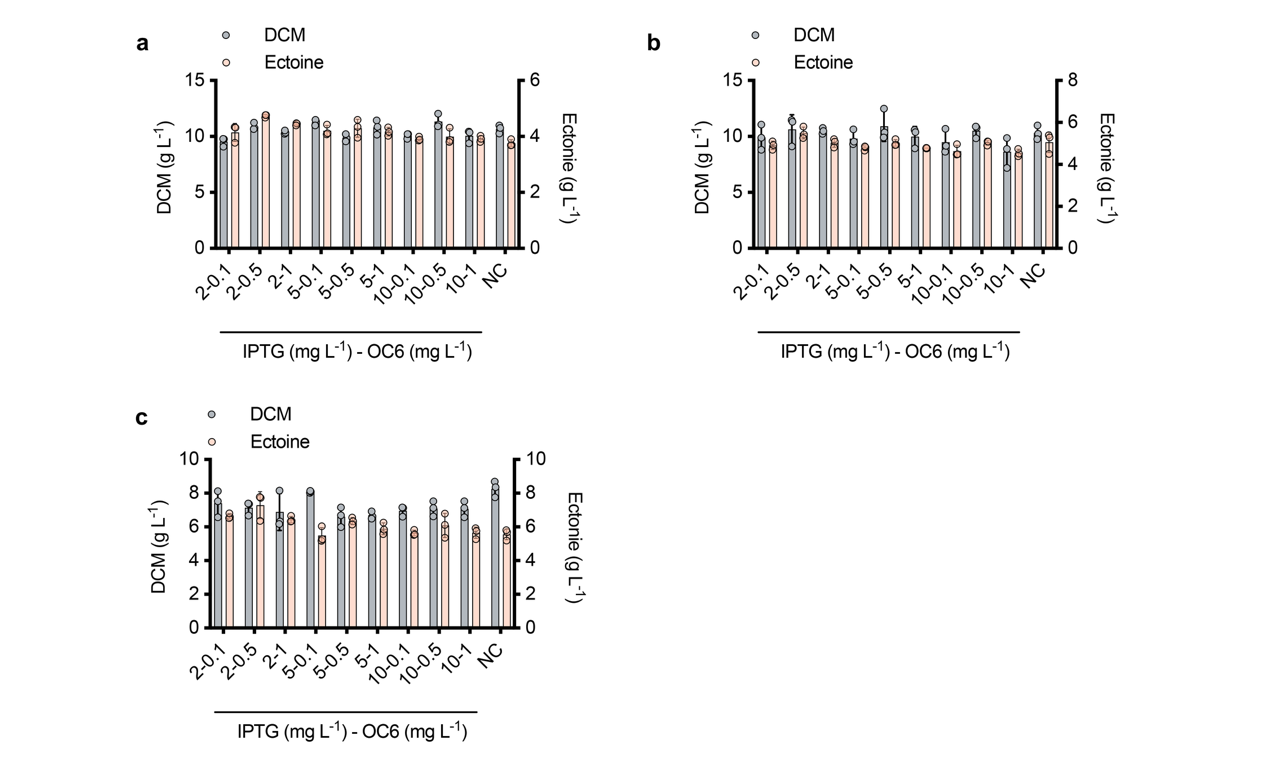
**

**Supplementary Fig. 14 Effects on ectoine synthesis by recombiant TD80 grown on different concentration of urea.**

DCM and ectoine titer obtained by recombinant TD80 from Supplementary Fig. 11b grown in 50MMA medium supplemented with 3 (**a**), 5 (**b**) and 7.5 (**c**) g L^-1^ urea, respectively, in the presence of 2 mg L^-1^ IPTG and 0.5 mg L^-1^ OC6 (shake flask). Error bars represent standard deviations, n = 3.

**
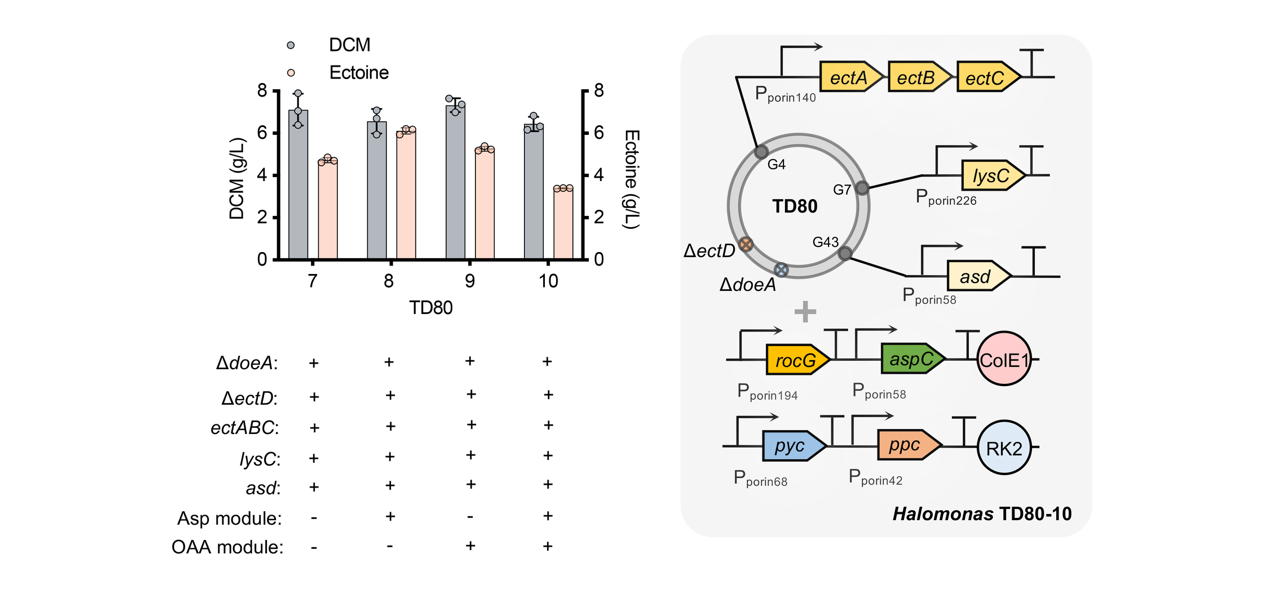
**

**Supplementary Fig. 15 Effects on ectoine synthesis by recombinant TD80-7 by enhancing aspartate and OAA supplying fluxes.**

DCM and ectoine accumulation by recombinant TD80-7 (group 7) harboring *rocG-aspC* (constructed on pSEVA321 (RK2) and pSEVA341 (ColE1)) and *pyc-ppc* modules, as well as their combination (TD80-10), respectively, grown in 50MMAU (5 g L^-1^ urea) medium (shake falsk). Error bars represent standard deviations, n = 3


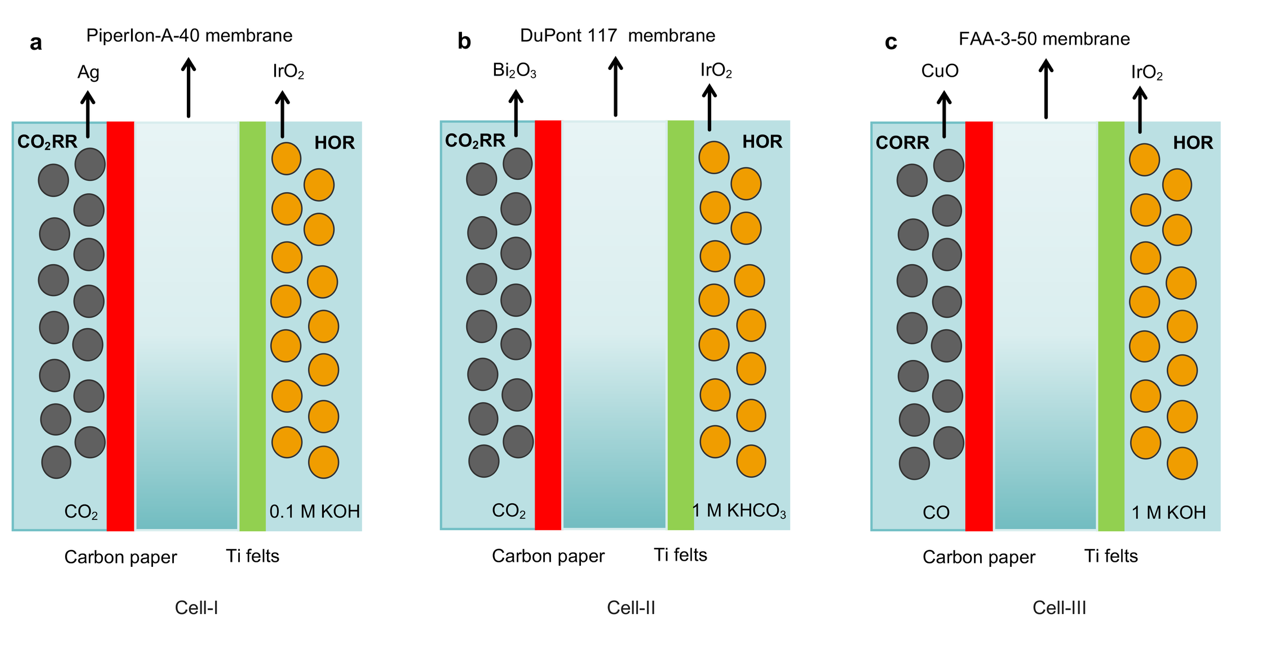


**Supplementary Fig. 16 Schematic illustration of the electrolyzer**

The electrochemical reaction process from CO_2_ to C_2+_ including three-step cascade reduction: from CO_2_ to CO in Cell-I (a), unreacted CO_2_ from Cell-I to formate in Cell-II (b), followed by CO to C_2+_ for acetate synthesis in Cell-III (c).


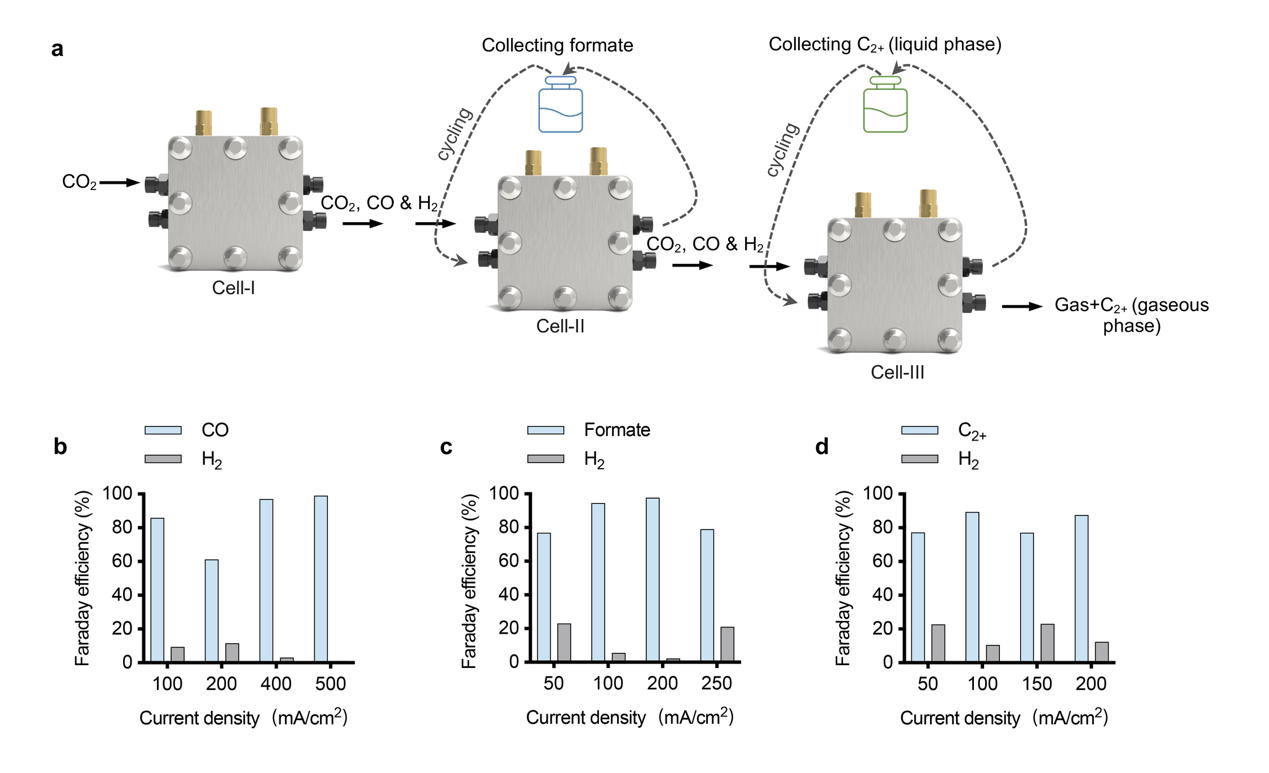


**Supplementary Fig. 17** **Developing electrochemical system for sufficient CO_2_-to-C_2+_ conversion.**

**a** Schematic diagram of electrochemical reaction process from CO_2_ to C_2+_ including three-step cascaded reduction: from CO_2_ to CO in Cell-I, unreacted CO_2_ from Cell-I to formate in Cell-II, followed by CO to C_2+_ for acetate synthesis in Cell-III. The electrochemical reaction would be suspended for electrolyte recycling once the concentration ratio of acetate *vs* formate reaches 9:1 measured by real-time NMR and a concentration detectorand, separately. **b-d** Faraday efficiency of reductions at different current densities from Cell-I (**b**), Cell-II (**c**) and Cell-III (**d**), respectively.

**
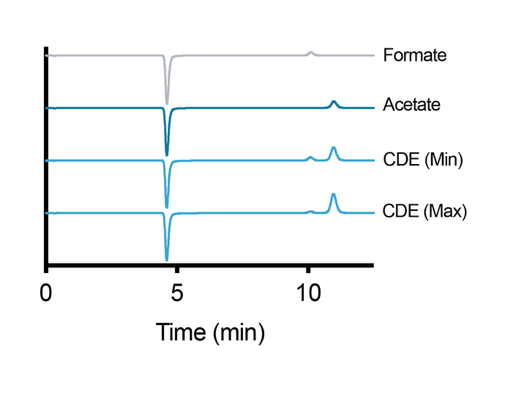
**

**Supplementary Fig. 18 HPLC analysis of formate, acetate and CDE**

The retention time of formate and acetate are approximately at 10.2 and 11.1 min, respectively. Source data are provided in Source Data file.

**
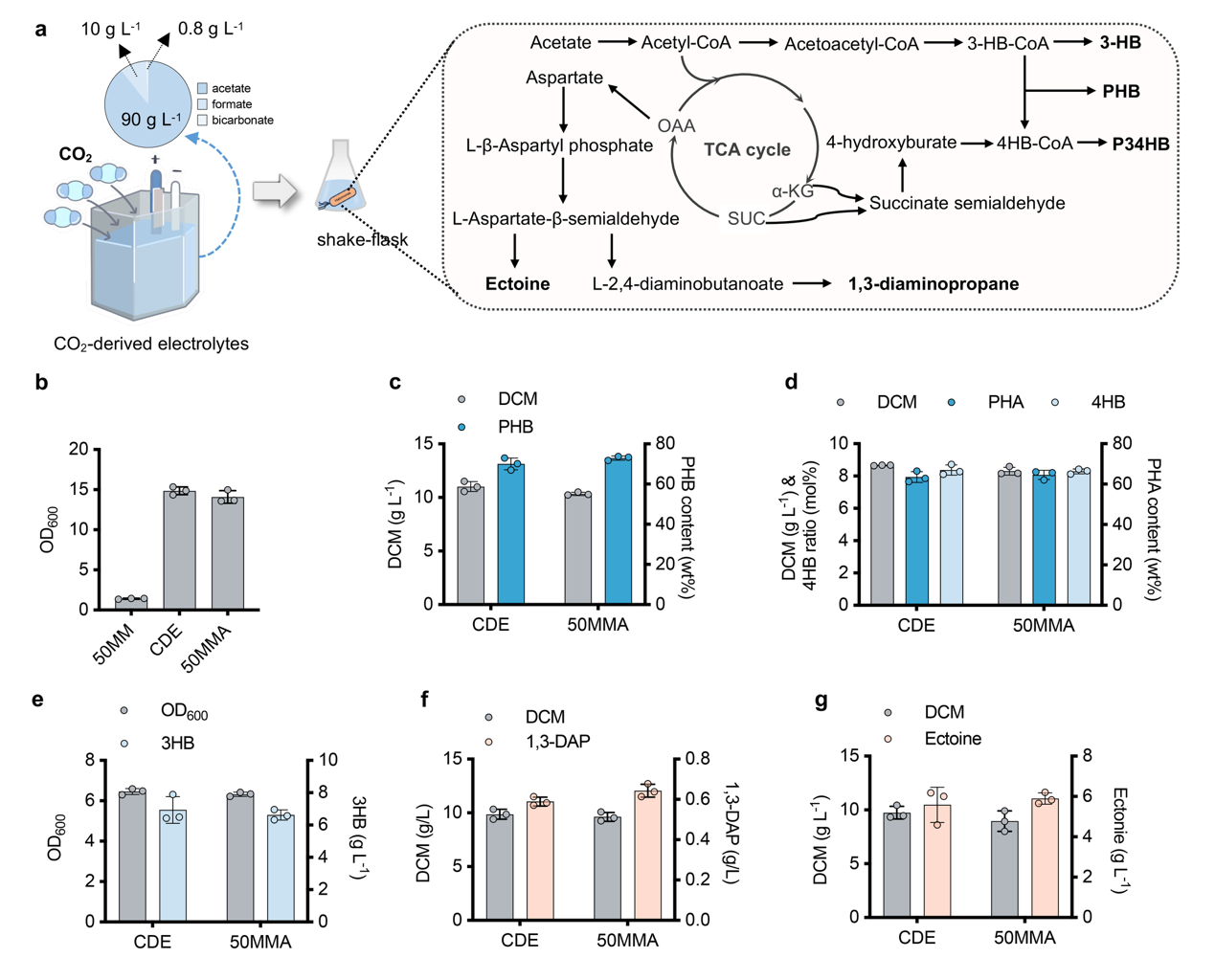
** **Supplementary Fig. 19 Biosynthesis assessment by recombinant TD80 strains grown on CDE**

**a** Biosynthesis roadmap of PHAs, 3HB, ectoine and 1,3-DAP by engineered TD80 strains using CDE as a sole carbon source conducted in 500-mL shake flask. **b** Shake flask study of cell growth (OD_600_) by TD80 grown in 50MM medium supplemented with CDE (CDE) and acetate (50MMA) compared to that without any carbon source supplementation (50MM). **c-d** Comparative analysis of DCM accumulation and PHA content, including PHB (**c**) and P34HB (**d**), by recombinant TD80 grown on acetate (50MMA) and CDE, respectively, as sole carbon source. **e-g** Shake flask studies for 3HB (**e**), 1,3-DAP (**f**) and ectoine (**g**) synthesis by engineered TD80 from acetate (50MMA) and CDE, respectively. Error bars represent standard deviations, n = 3.

**
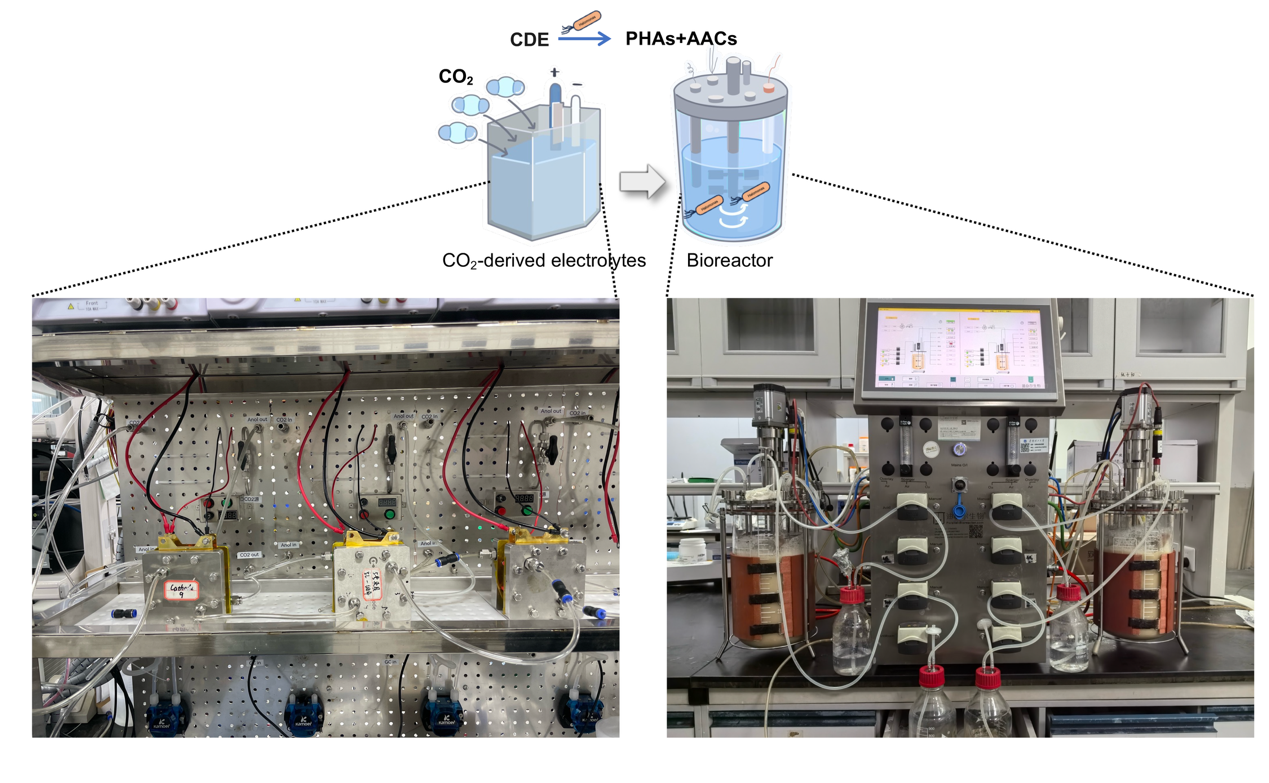
 Supplementary Fig. 20 Two major modular facilities of** ***Halomonas*-based electrochemical–biological hybrid system for CO_2_ upcycling*.***

Left: Three step-cascaded electrochemical system for sufficient CO_2_-to-C2+ conversion (Supplementary Figs 16-17); Right: Lab-scale bioconversion system powered by recombinant *Halomonas* for different fermentation product from CDE (produced by the facility from left panel).


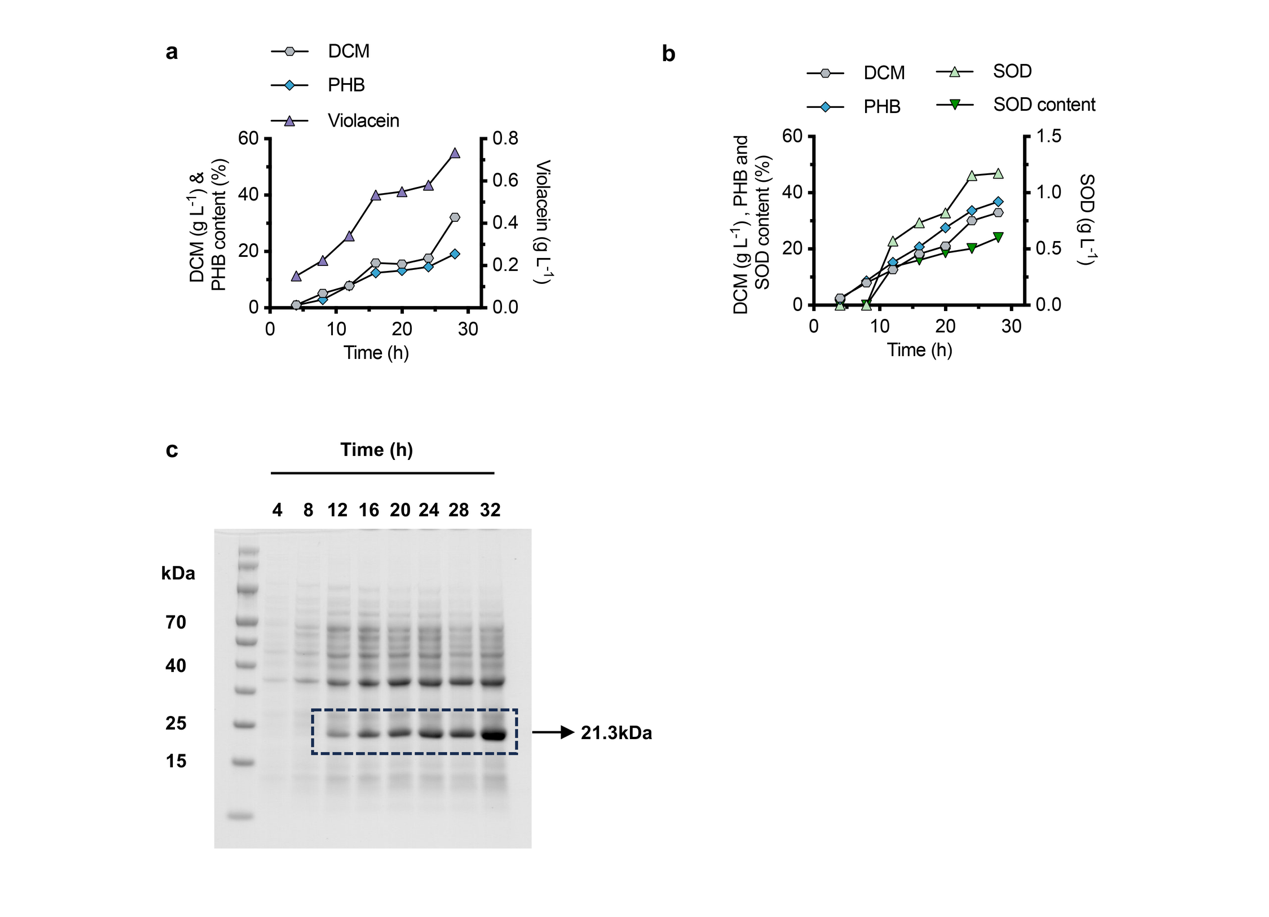


**Supplementary Fig. 21** **Fed-batch production of violacein and SOD by recombinant TD80.**

**a-b** Fed-batch study for violacein (**a**, 10 mg L^-1^ IPTG supplemented at 6 h) and SOD (**b**, 50 mg L^-1^ IPTG supplemented at 8 h) production by recombinant TD80 conducted in a 7-L bioreactor using CDE as sole carbon source (n=1). **c** Time-course SDS-PAGE analysis of SOD during the 32-h fed-batch study. Meanwhile, the co-production of PHB (wt%), together with violacein and SOD, respectively, and DCM were also studied in every 4 h throughout the fermentation process.

***
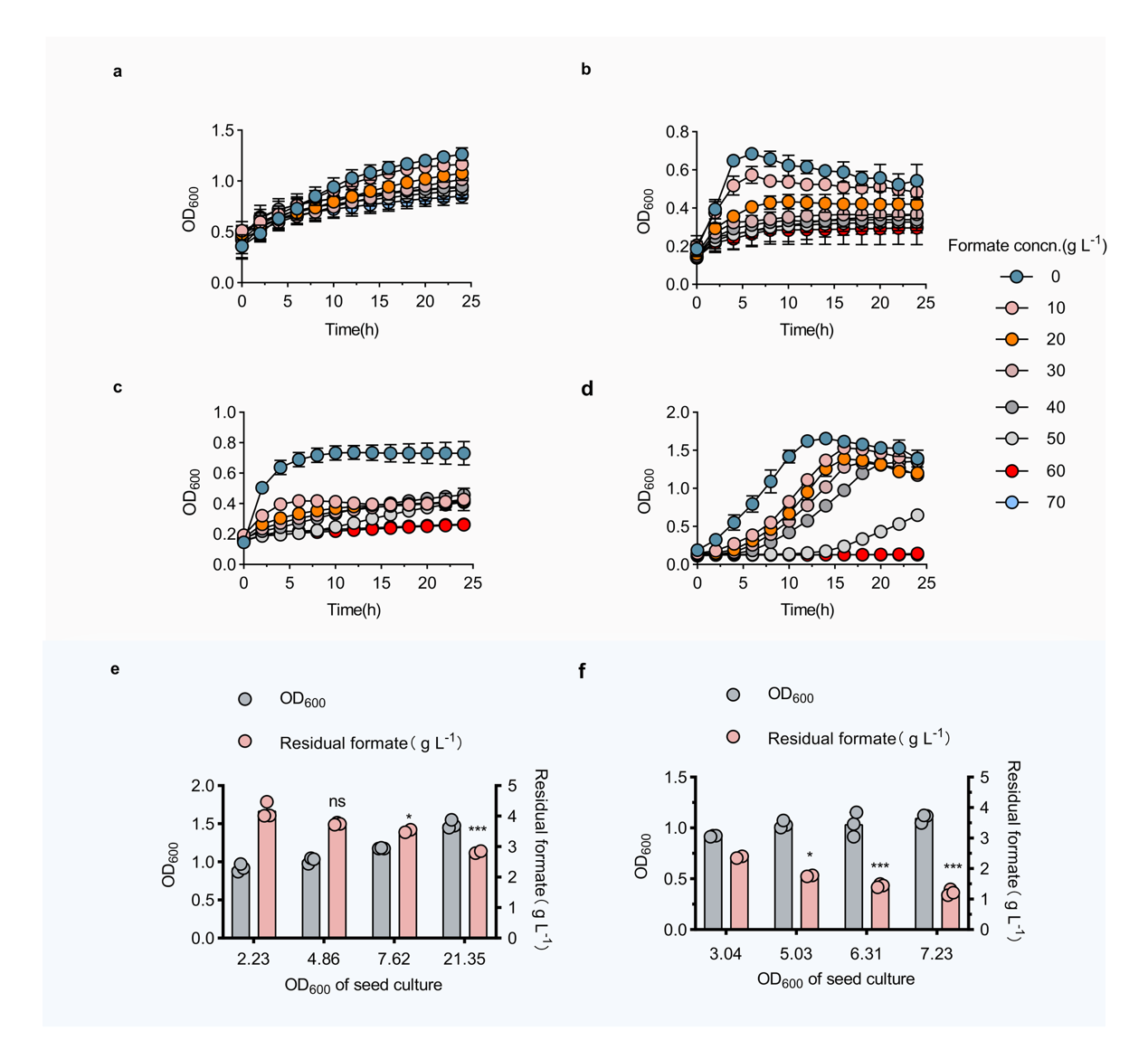
***

**Supplementary Fig. 22 Assement of formate tolerance of different chassis.**

Growth profiling (96-well plate) of different chassis, including TD80 (**a**), *Escherichia coli* MG1655 (**b**), *Vibrio* *natriegens* (**c**) and *Pseudomonas* *putida* KT2442 (**d**) cultured in mineral medium supplemented with each preference carbon source, together with 0, 10, 20, 30, 40, 50, 60, 70 g L^-1^ formate, respectively. TD80, 50MM medium containing 15 g L^-1^ acetate; MG1655 & KT2442, 10MM medium containing 11 g L^-1^ glucose; *Vibrio*, 20MM medium containing 11 g L^-1^ glucose. Error bars represent standard deviations, n = 3.


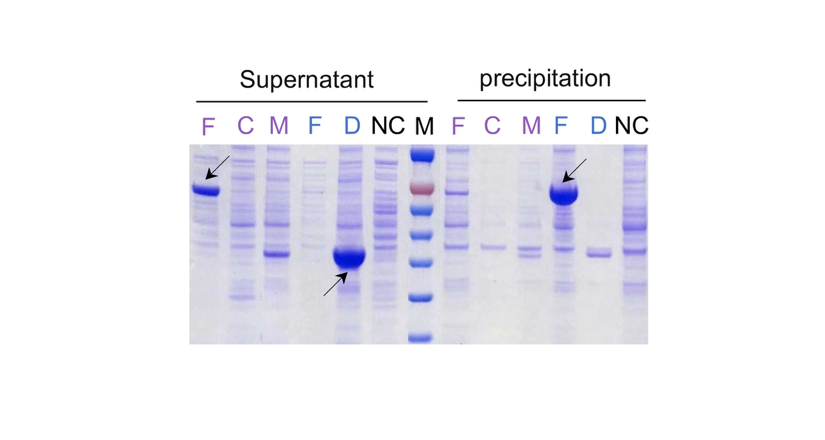


**Supplementary Fig. 23** **SDS-PAGE analysis of C1 module-related enzymes from *Vibrio natriegens* and *M. extorquens* AM1 in *E******. coli* BL21 (DE3).**

Evaluation of the key enzyme expression (level and solubility) involved in C1 module for formate utilization in recombinant *E. coli* BL21 (DE3). The enzymes were cloned from two hosts, *Vibrio natriegens* (letter blue) and *M. extorquens* AM1 (letter in purple), and expressed by 100 mg L^-1^ IPTG in recombinanr DE3 grown at 37 °C. Lanes: NC, wild-type DE3 as negative control; F in purple, Ftfl; C and M in purple, Fch and MtdA; F in blue, Ftl; D in blue, FolD.


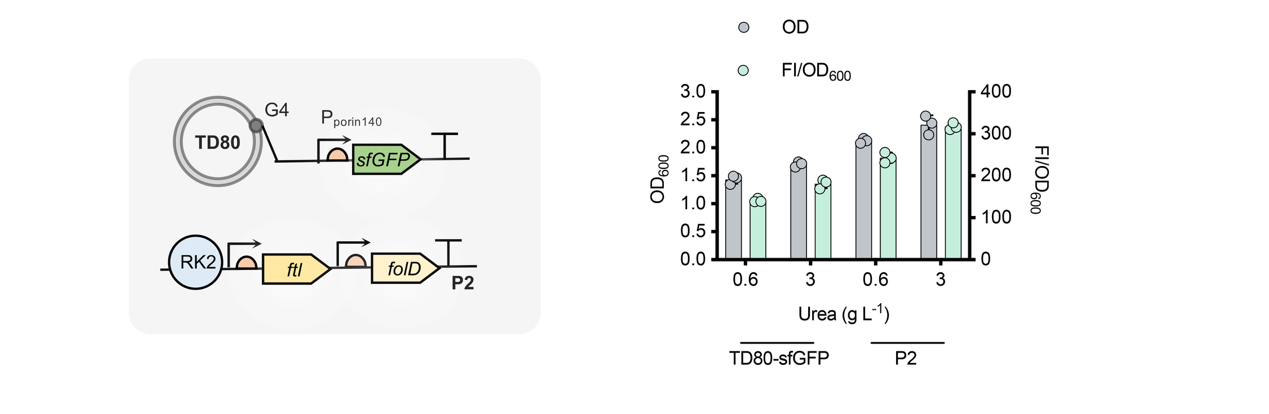
**Supplementary Fig. 24 Cell growth validation of recombinant TD80 harboring sfGFP reporter using formate only.**

Shake flask study of OD_600_ and FI by recombinantTD80-sfGFP harboring C1 module (*Ftl-FloD)* driven by P_Mmp1_ promoter grown on 50MMF (5 g L^-1^ formate,0.6 or 3 g L^-1^ urea) medium in the presence of 2 mg L^-1^ IPTG. FI was normalized by dividing OD_600_. Error bars represent standard deviations, n = 3.


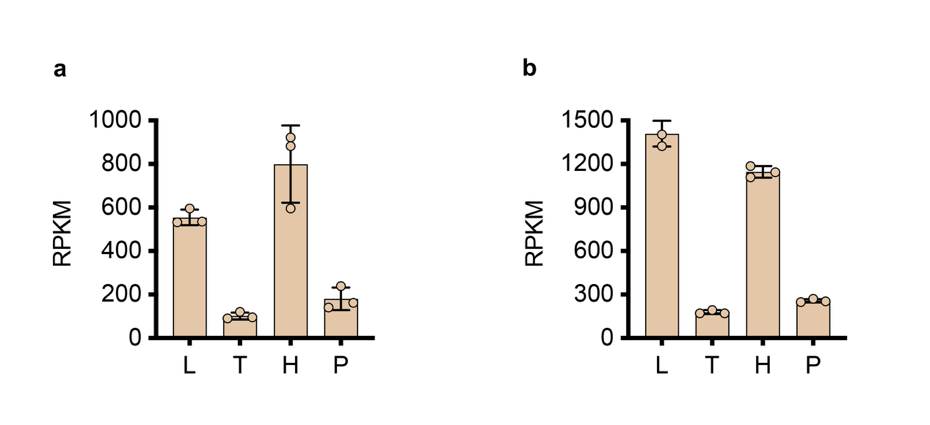


**Supplementary Fig. 25 Transcription of four endougenous enzymes (Lpd, GcvT, GcvH and GcvP) involved in GCS system, the main pathway of C2 module for formate utilization, of *Halomonas* TD grown in 60LB (a), and 50MMAU (b, 3.6 g L^-1^ urea). Source data of the mRNA level of target genes was obtained from the transcriptome in previous study^[28]^.**

**References**

1. Gong Q*, et al.* Structural defects on converted bismuth oxide nanotubes enable highly active electrocatalysis of carbon dioxide reduction. *Nat Commun* **10**, 2807 (2019).

2. Garg S, Xie Z, Chen JG. Tandem reactors and reactions for CO_2_ conversion. *Nature Chemical Engineering* **1**, 139-148 (2024).

3. Moller T, Filippi M, Bruckner S, Ju W, Strasser P. A CO_2_ electrolyzer tandem cell system for CO(2)-CO co-feed valorization in a Ni-N-C/Cu-catalyzed reaction cascade. *Nat Commun* **14**, 5680 (2023).

4. Zhang Y*, et al.* 2.6% cm^2^ Single-Pass CO_2_-to-CO conversion using Ni single atoms supported on ultra-thin carbon nanosheets in a flow electrolyzer. *ACS Catalysis* **11**, 12701-12711 (2021).

5. Hann EC*, et al.* A hybrid inorganic-biological artificial photosynthesis system for energy-efficient food production. *Nat Food* **3**, 461-471 (2022).

6. Kar S, Goeppert A, Galvan V, Chowdhury R, Olah J, Prakash GKS. A carbon-neutral CO_2_ capture, conversion, and utilization cycle with low-temperature regeneration of sodium hydroxide. *J Am Chem Soc* **140**, 16873-16876 (2018).

7. Shu Q, Legrand L, Kuntke P, Tedesco M, Hamelers HVM. Electrochemical regeneration of spent alkaline absorbent from direct air capture. *Environ Sci Technol* **54**, 8990-8998 (2020).

8. Sabatino F, Grimm A, Gallucci F, van Sint Annaland M, Kramer GJ, Gazzani M. A comparative energy and costs assessment and optimization for direct air capture technologies. *Joule* **5**, 2047-2076 (2021).

9. Keith DW, Holmes G, St. Angelo D, Heidel K. A Process for capturing CO_2_ from the atmosphere. *Joule* **2**, 1573-1594 (2018).

10. Simon R, Priefer U, Puhler A. A broad host mobilization system for in vivo genetic engineering: Transposon mutagenesis in Gram-negative bacteria. *Bio/Technolgy* **1**, 37-45 (1983).

11. Zhao H*, et al.* Novel T7-like expression systems used for *Halomonas*. *Metab Eng* **39**, 128-140 (2017).

12. Liu K-X*, et al.* Metabolic engineering of *Halomonas* for effective production of tryptophan-derived compounds. *Chemical Engineering Journal* **512**, (2025).

13. Lin YN*, et al.* Developing Quorum Sensing-Based Collaborative Dynamic Control System in *Halomonas* TD01. *Adv Sci (Weinh)* **12**, e2408083 (2025).

14. Shen R*, et al.* Promoter engineering for enhanced P(3HB- co-4HB) production by *Halomonas* bluephagenesis. *ACS Synth Biol* **7**, 1897-1906 (2018).

15. Zheng T*, et al.* Upcycling CO_2_ into energy-rich long-chain compounds via electrochemical and metabolic engineering. *Nature Catalysis* **5**, 388-396 (2022).

16. Tang H*, et al.* Metabolic engineering of yeast for the production of carbohydrate-derived foods and chemicals from C1–3 molecules. *Nature Catalysis* **7**, 21-34 (2023).

17. Lim J, Choi SY, Lee JW, Lee SY, Lee H. Biohybrid CO_2_ electrolysis for the direct synthesis of polyesters from CO_2_. *Proc Natl Acad Sci U S A* **120**, e2221438120 (2023).

18. Dinges I, Depentori I, Gans L, Holtmann D, Waldvogel SR, Stockl M. Coupling of CO(2) Electrolysis with parallel and semi-automated biopolymer synthesis - Ex-Cell and without downstream processing. *ChemSusChem*, e202301721 (2024).

19. Stockl M, Harms S, Dinges I, Dimitrova S, Holtmann D. From CO_2_ to bioplastic - coupling the electrochemical CO_2_ reduction with a microbial product generation by drop-in electrolysis. *ChemSusChem* **13**, 4086-4093 (2020).

20. Zhang P*, et al.* Chem-bio interface design for rapid conversion of CO_2_ to bioplastics in an integrated system. *Chem* **8**, 3363-3381 (2022).

21. Su Y*, et al.* Close-packed nanowire-bacteria hybrids for efficient solar-driven CO_2_ Fixation. *Joule* **4**, 800-811 (2020).

22. Zhang J, Jin B, Fu J, Wang Z, Chen T. Adaptive laboratory evolution of *Halomonas bluephagenesis* enhances acetate tolerance and utilization to produce Poly(3-hydroxybutyrate). *Molecules* **27**, (2022).

23. Liu C, Colon BC, Ziesack M, Silver PA, Nocera DG. Water splitting–biosynthetic system with CO_2_ reduction efficiencies exceeding photosynthesis. *Science (New York, NY)* **352**, 1210 (2016).

24. Cui H*, et al.* Converting CO_2_ to single-cell protein via an integrated electrocatalytic-biosynthetic system. *Applied Catalysis B: Environment and Energy*, 123946 (2024).

25. Roh H*, et al.* Improved CO_2_-derived polyhydroxybutyrate (PHB) production by engineering fast-growing cyanobacterium *Synechococcus elongatus* UTEX 2973 for potential utilization of flue gas. *Bioresour Technol* **327**, 124789 (2021).

26. Ma H*, et al.* Rational flux-tuning of *Halomonas bluephagenesis* for co-production of bioplastic PHB and ectoine. *Nat Commun* **11**, 3313 (2020).

27. Hu Q*, et al.* Ectoine hyperproduction by engineered *Halomonas bluephagenesis*. *Metab Eng* **82**, 238-249 (2024).

28. Zhang L, *, et al.* A long-term growth stable *Halomonas sp.* deleted with multiple transposases guided by its metabolic network model Halo-ecGEM. *Metab Eng* **84**, 95-108 (2024).

**Gene sequence:**

*tesB* (*Escherichia coli* MG1655): GenBank: CP144891.1 (protein id: XCY20697.1)

atgagtcaggcgctaaaaaatttactgacattgttaaatctggaaaaaattgaggaaggactctttcgcggccagagtgaagatttaggtttacgccaggtgtttggcggccaggtcgtgggtcaggccttgtatgctgcaaaagagaccgtccctgaagagcggctggtacattcgtttcacagctactttcttcgccctggcgatagtaagaagccgattatttatgatgtcgaaacgctgcgtgacggtaacagcttcagcgcccgccgggttgctgctattcaaaacggcaaaccgattttttatatgactgcctctttccaggcaccagaagcgggtttcgaacatcaaaaaacaatgccgtccgcgccagcgcctgatggcctcccttcggaaacgcaaatcgcccaatcgctggcgcacctgctgccgccagtgctgaaagataaattcatctgcgatcgtccgctggaagtccgtccggtggagtttcataacccactgaaaggtcacgtcgcagaaccacatcgtcaggtgtggatccgcgcaaatggtagcgtgccggatgacctgcgcgttcatcagtatctgctcggttacgcttctgatcttaacttcctgccggtagctctacagccgcacggcatcggttttctcgaaccggggattcagattgccaccattgaccattccatgtggttccatcgcccgtttaatttgaatgaatggctgctgtatagcgtggagagcacctcggcgtccagcgcacgtggctttgtgcgcggtgagttttatacccaagacggcgtactggttgcctcgaccgttcaggaaggggtgatgcgtaatcacaattaa

*phaA* (*Ralstonia eutropha* H16): GenBank: AM260479.1 (protein id: CAJ92573.1)

atgactgacgttgtcatcgtatccgccgcccgcaccgcggtcggcaagtttggcggctcgctggccaagatcccggcaccggaactgggtgccgtggtcatcaaggccgcgctggagcgcgccggcgtcaagccggagcaggtgagcgaagtcatcatgggccaggtgctgaccgccggttcgggccagaaccccgcacgccaggccgcgatcaaggccggcctgccggcgatggtgccggccatgaccatcaacaaggtgtgcggctcgggcctgaaggccgtgatgctggccgccaacgcgatcatggcgggcgacgccgagatcgtggtggccggcggccaggaaaacatgagcgccgccccgcacgtgctgccgggctcgcgcgatggtttccgcatgggcgatgccaagctggtcgacaccatgatcgtcgacggcctgtgggacgtgtacaaccagtaccacatgggcatcaccgccgagaacgtggccaaggaatacggcatcacacgcgaggcgcaggatgagttcgccgtcggctcgcagaacaaggccgaagccgcgcagaaggccggcaagtttgacgaagagatcgtcccggtgctgatcccgcagcgcaagggcgacccggtggccttcaagaccgacgagttcgtgcgccagggcgccacgctggacagcatgtccggcctcaagcccgccttcgacaaggccggcacggtgaccgcggccaacgcctcgggcctgaacgacggcgccgccgcggtggtggtgatgtcggcggccaaggccaaggaactgggcctgaccccgctggccacgatcaagagctatgccaacgccggtgtcgatcccaaggtgatgggcatgggcccggtgccggcctccaagcgcgccctgtcgcgcgccgagtggaccccgcaagacctggacctgatggagatcaacgaggcctttgccgcgcaggcgctggcggtgcaccagcagatgggctgggacacctccaaggtcaatgtgaacggcggcgccatcgccatcggccacccgatcggcgcgtcgggctgccgtatcctggtgacgctgctgcacgagatgaagcgccgtgacgcgaagaagggcctggcctcgctgtgcatcggcggcggcatgggcgtggcgctggcagtcgagcgcaaataa

*phaB* (*Ralstonia eutropha* H16): GenBank: J04987.1 (protein id: CAJ92574.1)

atgactcagcgcattgcgtatgtgaccggcggcatgggtggtatcggaaccgccatttgccagcggctggccaaggatggctttcgtgtggtggccggttgcggccccaactcgccgcgccgcgaaaagtggctggagcagcagaaggccctgggcttcgatttcattgcctcggaaggcaatgtggctgactgggactcgaccaagaccgcattcgacaaggtcaagtccgaggtcggcgaggttgatgtgctgatcaacaacgccggtatcacccgcgacgtggtgttccgcaagatgacccgcgccgactgggatgcggtgatcgacaccaacctgacctcgctgttcaacgtcaccaagcaggtgatcgacggcatggccgaccgtggctggggccgcatcgtcaacatctcgtcggtgaacgggcagaagggccagttcggccagaccaactactccaccgccaaggccggcctgcatggcttcaccatggcactggcgcaggaagtggcgaccaagggcgtgaccgtcaacacggtctctccgggctatatcgccaccgacatggtcaaggcgatccgccaggacgtgctcgacaagatcgtcgcgacgatcccggtcaagcgcctgggcctgccggaagagatcgcctcgatctgcgcctggttgtcgtcggaggagtccggtttctcgaccggcgccgacttctcgctcaacggcggcctgcatatgggctga

*4hbD* (*Clostridium kluyveri* DSM555): GenBank: CP000673.1 (protein id: EDK35022.1)

atgaagttattaaaattggcacctgatgtttataaatttgatactgcagaggagtttatgaaatactttaaggttggaaaaggtgactttatacttactaatgaatttttatataaacctttccttgagaaattcaatgatggtgcagatgctgtatttcaggagaaatatggactcggtgaaccttctgatgaaatgataaacaatataattaaggatattggagataaacaatataatagaattattgctgtagggggaggatctgtaatagatatagccaaaatcctcagtcttaagtatactgatgattcattggatttgtttgagggaaaagtacctcttgtaaaaaacaaagaattaattatagttccaactacatgtggaacaggttcagaagttacaaatgtatcagttgcagaattaaagagaagacatactaaaaaaggaattgcttcagacgaattatatgcaacttatgcagtacttgtaccagaatttataaaaggacttccatataagttttttgtaaccagctccgtagatgccttaatacatgcaacagaagcttatgtatctccaaatgcaaatccttatactgatatgtttagtgtaaaagctatggagttaattttaaatggatacatgcaaatggtagagaaaggaaatgattacagagttgaaataattgaggattttgttataggcagcaattatgcaggtatagcttttggaaatgcaggagtgggagcggttcacgcactctcatatccaataggcggaaattatcatgtgcctcatggagaagcaaattatctgttttttacagaaatatttaaaacttattatgagaaaaatccaaatggcaagattaaagatgtaaataaactattagcaggcatactaaaatgtgatgaaagtgaagcttatgacagtttatcacaacttttagataaattattgtcaagaaaaccattaagagaatatggaatgaaagaggaagaaattgaaacttttgctgattcagtaatagaaggacagcagagactgttggtaaacaattatgaacctttttcaagagaagacatagtaaacacatataaaaagttatattaa

*sucD* (*Clostridium kluyveri* DSM555): GenBank: CP000673.1 (protein id: BAH07714.1)

atgagtaatgaagtatctataaaagaattaattgaaaaggcaaaggtggcacaaaaaaaattggaagcctatagtcaagaacaagttgatgtactagtaaaagcactaggaaaagtggtttatgataatgcagaaatgtttgcaaaagaagcagttgaagaaacagaaatgggtgtttatgaagataaagtagctaaatgtcatttgaaatcaggagctatttggaatcatataaaagacaagaaaactgtaggcataataaaagaagaacctgaaagggcacttgtttatgttgctaagccaaagggagttgtggcagctactacgcctataactaatccagtggtaactcctatgtgtaatgcaatggctgctataaagggcagaaatacaataatagtagcaccacatcctaaagcaaagaaagtttcagctcatactgtagaacttatgaatgctgagcttaaaaaattgggagcaccagaaaatatcatacagatagtagaagcaccatcaagagaagctgctaaggaacttatggaaagtgctgatgtagttattgctacaggcggtgctggaagagttaaagctgcttactccagtggaagaccagcttatggcgttggacctggaaattcacaggtaatagttgataagggatacgattataacaaagctgcacaggatataataacaggaagaaaatatgacaatggaattatatgttcttcagagcaatcagttatagctcctgctgaagattatgataaggtaatagcagcttttgtagaaaatggggcattctatgtagaagatgaggaaacagtagaaaagtttagatcaactttatttaaagatggaaaaataaacagcaagattataggtaaatccgtccaaattattgcggatcttgcaggagtaaaagtaccagaaggtactaaggttatagtacttaagggtaaaggtgcaggagaaaaagatgtactttgtaaagaaaaaatgtgtccagttttagtagcattgaaatatgatacttttgaagaagcagttgaaatagctatggctaattatatgtatgaaggagctggtcatacagcaggcatacattctgacaatgacgagaacataagatatgcaggaactgtattacctataagcagattagttgtaaatcagcctgcaactactgctggaggaagtttcaataatggatttaaccctactactacactaggctgcggatcatggggcagaaacagtatttcagaaaatcttacttacgagcatcttataaatgtttcaagaatagggtatttcaataaagaagcaaaagttcctagctatgaggaaatatggggataa

*ogdA* (*Synechococcus sp.* PCC 7002): GenBank: CP000951.1 (protein id: ACB00744.1)

atgaatactgcagaattattgatccgatgtctagaaaatgaaggggtggagtatatttttgggctgccgggggaagaaaatctccatatcctcgaagcccttaaggagtctcccatccgctttatcaccgtccgccatgaacagggtgccgcttttatggccgatgtgtatggtcgtttaaccgggaaagcaggggtttgtctgtctaccctggggcctggggctaccaatctaatgactggggttgccgatgcgaacctcgatggggcgcccctgattgcgattacagggcaggtgggtaccgaccgcatgcacattgaatcccaccaatatcttgatctggtggcgatgtttgcccccgtcaccaagtggaataaacaaattgtccgaccgaacacgaccccggaggtggtacgtcgtgcctttaaaattgcccagcaggaaaaaccaggggcagtacacatcgatctccctgaaaatattgcggcgatgcccgtagaaggtcagcccctccagcgggatggtcgtgaaaaaatctatgcttcaagccggagtttaaaccgggctgccgaggcgatcgcccatgccaagagtcctttaattctggtgggtaatggcattattcgcgccgatgccgccgaagccctcaccgattttgccacccagttgaatattcccgtagtcaacacctttatgggcaaaggggcaattccctacacccatcccctgtccctgtggacggtaggactccaacagcgggattttgtcacctgtgcctttgaacagagcgatttggtgattgcagtgggctacgatctgatcgaatattcccccaaacgctggaacccagagggaacgaccccaattatccacattggtgaagtggccgccgaaattgatagtagttatattcccctcacagaagttgtcggcgacattggcgatgccttaaatgaaattcgtaaacgcacagaccgtgagggcaaaaccgcgccaaaatttctcaatgtccgggctgagattcgggaggactatgaacgccacggcaccgacgctagttttccggtcaaaccccaaaaaatcatctacgatctccgccaagtgatggccccagaggacatcgtcatttctgatgtgggggcccacaaaatgtggatggcccgccattaccattgcgatcgccccaatacttgcctgatttccaatggatttgcggcgatgggcattgcgattcccggtgctgtagcagccaaattagtctacccagaaaaaaatgtcgtggctgtcacaggggacgggggatttatgatgaactgccaggagctcgaaacggccctgcgcattggggcgaactttgtcaccctaattttcaatgatggtggctatggtttgatcggttggaaacagattaaccagttcggtgcaccagcctttgtggagtttggcaatcccgattttgtgcagtttgccgaaagtatgggcctcaagggttatcggattaccgccgccgccgaccttgtgccgaccttaaaagaagccctagcccaggatgtaccagcggtgatcgattgccccgtggactacagtgagaatgtgaaattctcccaaaaatcaggggatttaatctgccgtatgtaa

*orfZ* (*Clostridium kluyveri* DSM555): GenBank: CP000673.1 (/protein id: EDK35026.1)

atggagtgggaagagatatataaagagaaactggtaactgcagaaaaagctgtttcaaaaatagaaaaccatagcagggtagtttttgcacatgcagtaggagaacccgtagatttagtaaatgcactagttaaaaataaggataattatataggactagaaatagttcacatggtagctatgggcaaaggtgaatatacaaaagagggtatgcaaagacattttagacataatgctttatttgtaggcggatgtactagagatgcagtaaattcaggaagagcagattatacaccttgttttttctatgaagtgccaagtttgtttaaagaaaaacgtttgcctgtagatgtagcacttattcaggtaagtgagccagataaatatggctactgcagttttggagtttccaatgactataccaagccagcagcagaaagtgctaagcttgtaattgcagaagtgaataaaaacatgccaagaactcttggagattcttttatacatgtatcagatattgattatatagtggaagcttcacacccattgttagaattgcagcctcctaaattgggagatgtagaaaaagccataggagaaaactgtgcatctttaattgaagatggagctactcttcagcttggaataggtgctataccagatgcggtacttttattcttaaagaacaaaaagaatttaggaatacattctgagatgatatcagatggtgtgatggaactggtgaaggcaggggttatcaataacaagaaaaagaccctccatccaggcaaaatagttgtaacatttttaatgggaacaaaaaaattatatgattttgtaaacaataatccaatggtagaaacttattctgtagattatgtaaataatccactggtaattatgaaaaatgacaatatggtttcaataaattcttgtgttcaagtagacttaatgggacaagtatgttctgaaagtataggattgaaacagataagtggagtgggaggccaggtagattttattagaggagctaatctatcaaagggtggaaaggctattatagctataccttccacagctggaaaaggaaaagtttcaagaataactccacttctagatactggtgctgcagttacaacttctagaaatgaagtagattatgtagttactgaatatggtgttgctcatcttaagggcaaaactttaagaaatagggcaagagctctaataaatatcgctcatccaaaattcagagaatcattaatgaatgaatttaaaaagagattttag

*aldD* (*Pseudomonas putida* KT2440): GenBank: CP097526.1 (protein id: UUX74514.1)

atgcgttatgcacatcccggtaccgaaggcgcgaaggtttccttcaagagccgctacggcaactacatcggtggtgagttcgttactccggtaaaggggcagtacttcgaaaatacctccccggtgaatggcaagctgatcgctgagttcccccgctctactgccgaagacatcgacaaagccctggatgccgcccatgctgcggccgacgcctggggccgcacttcagtgcaggatcgttccaacgtgctgctgaagatcgccgaccgcatcgagcagaacctcgaactgctggccattaccgaaacctgggacaacggcaagccgatccgcgaaaccctcaacgccgacattccgctggcggtcgaccacttccgctatttcgctggctgcatccgcgcccaggaaggcggcgctgccgaaatcaatgaaggcaccgtggcctaccacatccacgagccactgggcgtggtcgggcagatcatcccctggaacttcccgatcctgatggctgcctggaagcttgccccggcactggcagctggtaactgcgtggtgctcaagcctgccgaacagacgccgctgggtatcaccgtactgctcgaagtcattggcgacctgttgccacctggcgtgctcaacgtagtgcaaggctatggccgcgaagccggtgaagccctggccaccagcaaacgcatcgccaagatcgccttcaccggctctaccccggtgggctcgcacatcatgaaatgcgccgccgagaacatcatcccgtccaccgtcgaactgggtggcaagtcgccgaacgtgtacttcgaagacatcatgcaggccgaaccaagcttcatcgagaaggcggcagaaggcatggtgctggcgttcttcaaccagggtgaagtgtgcacctgcccgtcgcgtgccctggtgcaagagtcgatctatccgcagttcatggaagtggtgatgaagaaggtgctgcagatcaagcgcggcgacccgttggacaccgacaccatggtcggcgcccaggcctcgcagcagcagttcgaaaagatcctctcgtacctgcagattgcccaggaagaaggcgccgagctgctcaccggcggcaaggtcgagaagctggaaggttcgctggctaccggttactacatccagccgaccctgctcaagggcaacaacaagatgcgcgtgttccaggaagaaatcttcggcccggtggtcagcgtcaccaccttcaaggacgaagccgaagcgctggcaattgccaacgacaccgagttcggcctgggcgccggcgtgtggacccgcgacatcaaccgtgcctaccgcatgggccggggcatcaaggctggccgtgtgtggaccaactgctaccacctgtacccggcgcatgccgcgttcggcgggtacaagaagtcgggggttgggcgtgagacgcacaagatgatgctggatcactatcagcagaccaagaacctgctggtgagctacgacatcaatccgctgggcttcttcta

*dhaT* (*Pseudomonas putida* KT2440): GenBank: CP097526.1 (protein id: UUX71387.1)

atgacaacaattgagcaagaggtacgtatggaattcaagtttctactccccagcaaaatcgtgatggaaccgggcctgcgcgagcgcaccggtgaacacctgcggcaactcggcctggcccgcgtgttgatcgtgaccgatgccggggtcaaggcagcaggcctgctggacagcgtctacgccagcctcgacaaggccggtatcgcctacgaggaagtggcggacatcaaggccaacccgcgcagtgacgacatcaaccacaccgcccagcgctatcggggcacgggtatcgacggcctgctggccgtgggtggcggcagcgcgatggatgcggccaaggccatcagcctgctgctcacccacgacggccgtatcgaggactacgaagggtcgttcacgcttacccatgccatcccgcccatcgtcgccatcccgaccacggccggcaccggcagcgaagtgacctgcttctcggtgatcaccgacaccgcccgccacttcaaaatgaacgtgctggactatcgcatcggcccggtgctggcattgctcgactcccatatcaccgacacgctgccgccgtcgattgcagccgcaaccggcatggatgccctgacccatgccatcgaagcctacacctgccgcgtggcaaacccgatcagcgacggcctggcgctgcatgccatccgcctgatcagccagcacctcaaggcggccgtgcaggagcccgacaaccaggcggcccgggagcagatgctggtggccagcctgattgccggcatggccttcggcaacgccgacgtcggcagcgtgcactgcatttccgaagccatcggcggcatgtacgacacgccccacggtgtgggcaacgcgatcttcctgcccttcgtgttcggtcacaaccgtgatgccgatatcgtccgccatgcccaggtcgcctacgcgttggggatcgaccccacgctgtctccggtcgatgccgccgaggccgccgtcggccatctgttccagatgagcaaggacctgggcatcccccgcttcgccgaggtcaagggtgtgcgcgaggaagacttcccgaccattgccgagaagtccaagcagaacttctcggacgccagcaatgccaaggcgatgtccgtagaggcctaccacgacatcatcaccaccgcttaccacttcgtcgcgtaa

*dat* (*Acinetobacter baumannii*): GenBank: CP088895.1 (protein id: UTH23704.1)

atgagcgttacttctgtcaaccctgccactaatgctaccaacgaatattatttgactcgccaaagtcaaatggaatcgaatgttcgtagctatccacgtaaattaccgttagcgatagcgaaagcacaaggttgctgggttactgatgttgaaggtacacagtaccttgattgtttagctggggcaggtacattggctttaggtcataatcatcctgcggtgattcaaagtattcaagacacattggcaagtggtttgccattgcatactttagacttaaccacacctttaaaagatgcgtttactgaagcgttgttagcatatttaccaggtggtaaagaagaatattgcttacagttctgtggtccttctggtgcagatgcgactgaggcagcaattaaacttgctaaaacttacactggccgtagctcagtgatcagtttctctggtggttaccatggtatgactcatggttcacttgccatgactggtaacttaagtgcgaaaaatgcagtgaatggcttaatgccaggcgtacaattcatgccatatccgcatgaatatcgctgcccacttggtttaggtggtgaagctggtgttgacgctttaacttactatttcgaaaactttattgaagatgttgaaagcggtgtaactaagccagctgctgttattcttgaagcaattcaaggtgaaggcggtgttgttacagctccagtaaaatggttgcaaaaaatccgtgaagtgactgaaaagcacaacatcgttttaatcttagacgaagttcaagcaggctttgctcgttcaggcaaaatgtttgcatttgaacatgccggtattgaacctgatgtcgttgtaatgtctaaagcagtaggtggtggtttaccacttgcagtattaggtattaagcgtaaatttgatgcttggcagcctgctggccacactggtactttccgtggtaaccaacttgctatgggtacaggtcttgttgtattagaaacgatcaaggaacaaaaccttgctcaaaatgcacaagagcgtggtgaattcttacaagctgaattgaaaaaattagctactgaatttccatgtatcggtaacgtacgtggccgcggtttaatgattggtgttgaaatcgttgacgagcgtaaacctgctgaccgtatcggttctcaccctgctgactctcagttagcggctgcgatccagactgcttgctttaacaacaagttattgcttgaaaaaggtggtcgtaacggtacagtaattcgtttactttgcccactcatcattactcaagaagagtgtgtagaagtaattgctcgctttaagaaagcagttgcagaagcattggttgcagtgcgaggcgcgtaa

*ddc* (*Acinetobacter baumannii*): GenBank: CP088895.1 (protein id: BAP65850.1)

atggtggattttgcagaacatcgtaaagcgttactctgcaatgatgcacaatccattgctgactatgagtcagcaatgggcgaagcggtaaaagccgtttcagcatggttgcaaaatgaaaaaatgtacacaggcggtagcattaaagagttgcgttcagcaatttctttccaaccttcaaaagaaggtatgggtgtacaacaatctcttcaacgtatgattgagcttttcttgaataaaagcttgaaagtacaccatccacactcattagcacatttacactgcccaaccatggtgatgagccagatcgcggaagtgttaatcaatgcaactaaccagtctatggactcatgggatcaaagcccggcaggttcattaatggaagttcagcttattgattggttgcgtcaaaaagtaggttatggttctggtcaggctggtgttttcacttctggcggtactcaatctaacttgatgggtgtgttacttgctcgtgactggtgcattgcgaaaaactggaaagacgaaaatggtaacccatggtctgtacagcgtgatggtattccagctgaagcaatgaaaaacgtcaaagtcatttgttctgaaaatgcgcatttctctgtgcaaaagaacatggcaatgatgggcatgggcttccagtcagttgtgactgttcctgtgaatgaaaatgcacagatggatgttgatgctcttgaaaaaacaatggcgcatcttcaagctgaaggtaacgttgttgcttgtgtcgttgcgacagcaggtacaaccgatgctggtgcaattgatccattaaaaaaaatccgtgaaattaccaataaatatggttcatggatgcatattgatgctgcgtggggcggtgcactgatcttgtcaaatgactatcgtgcaatgcttgatggtattgagctatctgattcgatcactctcgacttccataagcattatttccaaagcatcagctgtggcgcgttcttgttaaaagatgaagcgaactatcgtttcatgcattatgaagctgagtacttgaactctgcttatgatgaagagcatggcgtacctaaccttgtgtctaaatcattacaaacgactcgtcgttttgatgctttaaaattgtggatgaccattgaatcactcggcgaagagctatatggttcaatgattgatcatggtgtaaaactgactcgtgaagttgcagattacatcaaggcaactgatggtttagagcttttagttgaaccacaatttgcttcggtattgttccgtgttgttccggaaggttacccagttgagtttatcgatagcttgaaccaaaacgtagcagatgaattgtttgcacgtggtgaagcaaatattggtgtaacaaaagttggtaatgtacagtcattgaagatgacaacattaagccctgtagtaactgtcgacaacgttaagaaccttttagctcaagtattggctgaagctgaacgtattaaagatgcgattgcttctggtaactacgtaccaccaatcgactaa

*ppc* (*Corynebacterium glutamicum* 13032): GenBank: BA000036.3 (protein id: BAB98978.1)

atgactgattttttacgcgatgacatcaggttcctcggtcaaatcctcggtgaggtaattgcggaacaagaaggccaggaggtttatgaactggtcgaacaagcgcgcctgacttcttttgatatcgccaagggcaacgccgaaatggatagcctggttcaggttttcgacggcattactccagccaaggcaacaccgattgctcgcgcattttcccacttcgctctgctggctaacctggcggaagacctctacgatgaagagcttcgtgaacaggctctcgatgcaggcgacacccctccggacagcactcttgatgccacctggctgaaactcaatgagggcaatgttggcgcagaagctgtggccgatgtgctgcgcaatgctgaggtggcgccggttctgactgcgcacccaactgagactcgccgccgcactgtttttgatgcgcaaaagtggatcaccacccacatgcgtgaacgccacgctttgcagtctgcggagcctaccgctcgtacgcaaagcaagttggatgagatcgagaagaacatccgccgtcgcatcaccattttgtggcagaccgcgttgattcgtgtggcccgcccacgtatcgaggacgagatcgaagtagggctgcgctactacaagctgagccttttggaagagattccacgtatcaaccgtgatgtggctgttgagcttcgtgagcgtttcggcgagggtgttcctttgaagcccgtggtcaagccaggttcctggattggtggagaccacgacggtaacccttatgtcaccgcggaaacagttgagtattccactcaccgcgctgcggaaaccgtgctcaagtactatgcacgccagctgcattccctcgagcatgagctcagcctgtcggaccgcatgaataaggtcaccccgcagctgcttgcgctggcagatgcagggcacaacgacgtgccaagccgcgtggatgagccttatcgacgcgccgtccatggcgttcgcggacgtatcctcgcgacgacggccgagctgatcggcgaggacgccgttgagggcgtgtggttcaaggtctttactccatacgcatctccggaagaattcttaaacgatgcgttgaccattgatcattctctgcgtgaatccaaggacgttctcattgccgatgatcgtttgtctgtgctgatttctgccatcgagagctttggattcaacctttacgcactggatctgcgccaaaactccgaaagctacgaggacgtcctcaccgagcttttcgaacgcgcccaagtcaccgcaaactaccgcgagctgtctgaagcagagaagcttgaggtgctgctgaaggaactgcgcagccctcgtccgctgatcccgcacggttcagatgaatacagcgaggtcaccgaccgcgagctcggcatcttccgcaccgcgtcggaggctgttaagaaattcgggccacggatggtgcctcactgcatcatctccatggcatcatcggtcaccgatgtgctcgagccgatggtgttgctcaaggaattcggactcatcgcagccaacggcgacaacccacgcggcaccgtcgatgtcatcccactgttcgaaaccatcgaagatctccaggccggcgccggaatcctcgacgaactgtggaaaattgatctctaccgcaactacctcctgcagcgcgacaacgtccaggaagtcatgctcggttactccgattccaacaaggatggcggatatttctccgcaaactgggcgctttacgacgcggaactgcagctcgtcgaactatgccgatcagccggggtcaagcttcgcctgttccacggccgtggtggcaccgtcggccgcggtggcggaccttcctacgacgcgattcttgcccagcccaggggggctgtccaaggttccgtgcgcatcaccgagcagggcgagatcatctccgctaagtacggcaaccccgaaaccgcgcgccgaaacctcgaagccctggtctcagccacgcttgaggcatcgcttctcgacgtctccgaactcaccgatcaccaacgcgcgtacgacatcatgagtgagatctctgagctcagcttgaagaagtacgcctccttggtgcacgaggatcaaggcttcatcgattacttcacccagtccacgccgctgcaggagattggatccctcaacatcggatccaggccttcctcacgcaagcagacctcctcggtggaagatttgcgagccatcccatgggtgctcagctggtcacagtctcgtgtcatgctgccaggctggtttggtgtcggaaccgcattagagcagtggattggcgaaggggagcaggccacccaacgcattgccgagctgcaaacactcaatgagtcctggccatttttcacctcagtgttggataacatggctcaggtgatgtccaaggcagagctgcgtttggcaaagctctacgcagacctgatcccagatacggaagtagccgagcgagtctattccgtcatccgcgaggagtacttcctgaccaagaagatgttctgcgtaatcaccggctctgatgatctgcttgatgacaacccacttctcgcacgctctgtccagcgccgatacccctacctgcttccactcaacgtgatccaggtagagatgatgcgacgctaccgaaaaggcgaccaaagcgagcaagtgtcccgcaacattcagctgaccatgaacggtctttccactgcgctgcgcaactccggctag

*pyc* (*Corynebacterium glutamicum* 13032): GenBank: BA000036.3 (protein id: BAB98082.1)

agtcgactcacacatcttcaacgcttccagcattcaaaaagatcttggtagcaaaccgcggcgaaatcgcggtccgtgctttccgtgcagcactcgaaaccggtgcagccacggtagctatttacccccgtgaagatcggggatcattccaccgctcttttgcttctgaagctgtccgcattggtaccgaaggctcaccagtcaaggcgtacctggacatcgatgaaattatcggtgcagctaaaaaagttaaagcagatgccatttacccgggatacggcttcctgtctgaaaatgcccagcttgcccgcgagtgtgcggaaaacggcattacttttattggcccaaccccagaggttcttgatctcaccggtgataagtctcgcgcggtaaccgccgcgaagaaggctggtctgccagttttggcggaatccaccccgagcaaaaacatcgatgagatcgttaaaagcgctgaaggccagacttaccccatctttgtgaaggcagttgccggtggtggcggacgcggtatgcgttttgttgcttcacctgatgagcttcgcaaattagcaacagaagcatctcgtgaagctgaagcggctttcggcgatggcgcggtatatgtcgaacgtgctgtgattaaccctcagcatattgaagtgcagatccttggcgatcacactggagaagttgtacacctttatgaacgtgactgctcactgcagcgtcgtcaccaaaaagttgtcgaaattgcgccagcacagcatttggatccagaactgcgtgatcgcatttgtgcggatgcagtaaagttctgccgctccattggttaccagggcgcgggaaccgtggaattcttggtcgatgaaaagggcaaccacgtcttcatcgaaatgaacccacgtatccaggttgagcacaccgtgactgaagaagtcaccgaggtggacctggtgaaggcgcagatgcgcttggctgctggtgcaaccttgaaggaattgggtctgacccaagataagatcaagacccacggtgcagcactgcagtgccgcatcaccacggaagatccaaacaacggcttccgcccagataccggaactatcaccgcgtaccgctcaccaggcggagctggcgttcgtcttgacggtgcagctcagctcggtggcgaaatcaccgcacactttgactccatgctggtgaaaatgacctgccgtggttccgactttgaaactgctgttgctcgtgcacagcgcgcgttggctgagttcaccgtgtctggtgttgcaaccaacattggtttcttgcgtgcgttgctgcgggaagaggacttcacttccaagcgcatcgccaccggattcattgccgatcacccgcacctccttcaggctccacctgctgatgatgagcagggacgcatcctggattacttggcagatgtcaccgtgaacaagcctcatggtgtgcgtccaaaggatgttgcagctcctatcgataagctgcctaacatcaaggatctgccactgccacgcggttcccgtgaccgcctgaagcagcttggcccagccgcgtttgctcgtgatctccgtgagcaggacgcactggcagttactgataccaccttccgcgatgcacaccagtctttgcttgcgacccgagtccgctcattcgcactgaagcctgcggcagaggccgtcgcaaagctgactcctgagcttttgtccgtggaggcctggggcggcgcgacctacgatgtggcgatgcgtttcctctttgaggatccgtgggacaggctcgacgagctgcgcgaggcgatgccgaatgtaaacattcagatgctgcttcgcggccgcaacaccgtgggatacaccccgtacccagactccgtctgccgcgcgtttgttaaggaagctgccagctccggcgtggacatcttccgcatcttcgacgcgcttaacgacgtctcccagatgcgtccagcaatcgacgcagtcctggagaccaacaccgcggtagccgaggtggctatggcttattctggtgatctctctgatccaaatgaaaagctctacaccctggattactacctaaagatggcagaggagatcgtcaagtctggcgctcacatcttggccattaaggatatggctggtctgcttcgcccagctgcggtaaccaagctggtcaccgcactgcgccgtgaattcgatctgccagtgcacgtgcacacccacgacactgcgggtggccagctggcaacctactttgctgcagctcaagctggtgcagatgctgttgacggtgcttccgcaccactgtctggcaccacctcccagccatccctgtctgccattgttgctgcattcgcgcacacccgtcgcgataccggtttgagcctcgaggctgtttctgacctcgagccgtactgggaagcagtgcgcggactgtacctgccatttgagtctggaaccccaggcccaaccggtcgcgtctaccgccacgaaatcccaggcggacagttgtccaacctgcgtgcacaggccaccgcactgggccttgcggatcgtttcgaactcatcgaagacaactacgcagccgttaatgagatgctgggacgcccaaccaaggtcaccccatcctccaaggttgttggcgacctcgcactccacctcgttggtgcgggtgtggatccagcagactttgctgccgatccacaaaagtacgacatcccagactctgtcatcgcgttcctgcgcggcgagcttggtaaccctccaggtggctggccagagccactgcgcacccgcgcactggaaggccgctccgaaggcaaggcacctctgacggaagttcctgaggaagagcaggcgcacctcgacgctgatgattccaaggaacgtcgcaatagcctcaaccgcctgctgttcccgaagccaaccgaagagttcctcgagcaccgtcgccgcttcggcaacacctctgcgctggatgatcgtgaattcttctacggcctggtcgaaggccgcgagactttgatccgcctgccagatgtgcgcaccccactgcttgttcgcctggatgcgatctctgagccagacgataagggtatgcgcaatgttgtggccaacgtcaacggccagatccgcccaatgcgtgtgcgtgaccgctccgttgagtctgtcaccgcaaccgcagaaaaggcagattcctccaacaagggccatgttgctgcaccattcgctggtgttgtcaccgtgactgttgctgaaggtgatgaggtcaaggctggagatgcagtcgcaatcatcgaggctatgaagatggaagcaacaatcactgcttctgttgacggcaaaatcgatcgcgttgtggttcctgctgcaacgaaggtggaaggtggcgacttgatcgtcgtcgtttcctaa

*rocG* (*Bacillus subtilis* 168): GenBank: CP051860.2 (protein id: QJF42530.1)

atgtcagcaaagcaagtctcgaaagatgaagaaaaagaagctcttaacttatttctgtctacccaaacaatcattaaggaagcccttcggaagctgggttatccgggagatatgtatgaactcatgaaagagccgcagagaatgctcactgtccgcattccggtcaaaatggacaatgggagcgtcaaagtgttcacaggctaccggtcacagcacaatgatgctgtcggtccgacaaaggggggcgttcgcttccatccagaagttaatgaagaggaagtaaaggcattatccatttggatgacgctcaaatgcgggattgccaatcttccttacggcggcgggaagggcggtattatttgtgatccgcggacaatgtcatttggagaactggaaaggctgagcagggggtatgtccgtgccatcagccagatcgtcggtccgacaaaggatattccagctcccgatgtgtacaccaattcgcagattatggcgtggatgatggatgagtacagccggctgcgggaattcgattctccgggctttattacaggtaaaccgcttgttttgggaggatcgcaaggacgggaaacagcgacggcacagggcgtcacgatttgtattgaagaggcggtgaagaaaaaagggatcaagctgcaaaacgcgcgcatcatcatacagggctttggaaacgcgggtagcttcctggccaaattcatgcacgatgcgggcgcgaaggtgatcgggatttctgatgccaatggcgggctctacaacccagacggccttgatatcccttatttgctcgataaacgggacagctttggtatggtcaccaatttatttactgacgtcatcacaaatgaggagctgcttgaaaaggattgcgatattttagtgcctgccgcgatctccaatcaaatcacagccaaaaacgcacataacattcaggcgtcaatcgtcgttgaagcggcgaacggcccgacaaccattgatgccactaagatcctgaatgaaagaggcgtgctgcttgtgccggatatcctagcgagtgccggcggcgtcacggtttcttattttgaatgggtgcaaaacaaccaaggatattattggtcggaagaagaggttgcagaaaaactgagaagcgtcatggtcagctcgttcgaaacaatttatcaaacagcggcaacacataaagtggatatgcgtttggcggcttacatgacgggcatcagaaaatcggcagaagcatcgcgtttccgcggatgggtctaa

*aspC* (*Corynebacterium glutamicum* 13032): GenBank: BA000036.3 (protein id: BAB97633.1)

atgagttcagtttcgctgcaggattttgatgcagagcgaattggtttgttccacgaggacattaagcgcaagtttgatgagctcaagtcaaaaaatctgaagctggatcttactcgcggtaagccttcgtcggagcagttggatttcgctgatgagttgttggcgttgcctggtaagggtgatttcaaggctgcggatggtactgatgtccgtaactatggcgggctggatggcatcgttgatattcgccagatttgggcggatttgctgggtgttcctgtggagcaggtcttggcgggggatgcttcgagcttgaacatcatgtttgatgtgatcagctggtcgtacattttcggtaacaatgattcggttcagccttggtcgaaggaagaaaccgttaagtggatttgccctgttccgggctatgatcgccatttctccatcacggagcgtttcggctttgagatgatttctgtgccaatgaatgaagacggccctgatatggatgctgttgaggaattggtgaagaatccgcaggttaagggcatgtgggttgttccggtgttttctaacccgactggtttcacggtgacagaagacgtcgcaaagcgtctaagcgcaatggaaaccgcagctccggacttccgcgttgtgtgggataatgcctacgccgttcatacgctgaccgatgaattccctgaggttatcgatatcgtcgggcttggtgaggccgctggcaacccgaaccgtttctgggcgttcacttctacttcgaagatcactctcgcgggtgcgggcgtgtcgttcttcctcacctctgcggagaaccgcaagtggtacaccggccatgcgggtatccgtggcattggccctaacaaggtcaatcagttggctcatgcgcgttactttggcgatgctgagggagtgcgcgcggtgatgcgtaagcatgctgcgtcgttggctccgaagttcaacaaggttctggagattctggattctcgccttgctgagtacggtgtcgcgcagtggactgtccctgcgggcggttacttcatttcccttgatgtggttcctggtacggcgtctcgcgtggctgagttggctaaggaagccggcatcgcgttgacgggtgcgggttcttcttacccgctgcgtcaggatccggagaacaaaaatctccgtttggcaccgtcgctgcctccagttgaggaacttgaggttgccatggatggcgtggctacctgtgtgctgttggcagcagcggagcattacgctaactaa

*ectA* (*Halomonas* TD): GenBank: OV350343.1 (protein id: CAH1044641.1)

atgagtacgccaataacaccttttaccccttctgcagaccttgctcgcccgacagttgctgatgctgtcgttgggcatacgtctacaccgttgtttattcgcaagccgaatgcagatgatggttggggcgtttatgagttaatcaaagcttgtccgccgcttgatgttaattctgcttatgcctacctgttgctagcaacacagtttcgtgacacgtgtgcagttgctaccaatgaagagggcgaaattgtcggttttgtatctggctatgtgaaagataatgcaccggatacgtattttctctggcaagttgctgtcggcgaaaaagctcgtggtaccggtttggcgcgccgcttggttgaagccattatgtcgcggccagaactcgataatgtgcatcatctggaaacgacgatcactccagacaatcaagcttcttggggattgtttcgccgtctagcagcacgttggcaagcccctctaaacagccgtgaatatttctctaccgaacagctcggtggagagcatgatccagaaaatttagtccgtataggtccattccaaacggacaacatgtaa

*ectB* (*Halomonas* TD)*:* GenBank: OV350343.1 (protein id: CAH1044642.1)

atgcagacccagacgctagaacgcatggaatctaacgtacgcacttattcgcgttcattccccgttgtgtttactaaagctcaaaacgctcgtttaacggatgagaatggtcgcgagtacattgatttcttagccggtgctggcactctcaattacggtcacaacaatccccacctcaagcaggcaatgatcgactatctggcgacagacggcattgttcatggcctggatatgtggaccgcagcaaagcgcgattacctggaaaccctagaagaagttattttcaaaccacgtggtttagactacaaggtacatctgcctggcccgacaggtactaacgcagtagaagcggccatccgtttggcccgtgttgctaaaggtcgccataacattgtgaccttcaccaacggcttccacggcgtcactatgggagcattggccaccacgggtaatcgtaagttccgcgaagcaactggcggtatccctacacagggggcaagctttttaccctttgatggttacatgggtgaacacgcggatacgctggattactttgaaaaattacttaacgacaaatctggcggcctcgatattccagcaggcgttattgttgaaaccgtgcaaggcgagggcggtattaacgtagcgggcattgagtggctcaaacgtttagaaagtatctgtcacgcccatgatattttgttgatcatcgacgacatacaagcgggctgtggccgtacgggtaagttctttagcttcgagcatgcgggcataacacctgacatcgtgaccaattcaaaatctctctctggttttgggatgccatttgcccacgtattgatgcgcccagagcttgataagtggaaacctggccagtacaacggcacgttccgtggcttcagcttggcgatggtaaccgccacagctgcacttaaaaaatattggtcaaatgatgtttttgagcgtgatgttcagcgtaaagcgcgtattgttgaagaacgtttccaaaagctagcagccttactcagcgagaacggcatgcctgccaccgaacgtggacgtggcctcatgcgtggtattgatgttgtctctggtgatattgctgacaagatcactagtaaagcgtttgaacacggccttattatcgagaccagtggccaagacggcgaagtagtgaagtgcctatgcccgctgaccatcagtgatgaggacttactggaagcgctggatattctagaagcctcggtcaatgctgttatccaagcgtga

*ectC* (*Halomonas* TD)*:* GenBank: OV350343.1 (protein id: CAH1044643.1)

atgatcgttcgtaatcttgaagaagcacgtaaaacagatcgtcttgttactgccgaaaatggcaactgggacagcacgcgtctagttctagccaacgacaatgcaggtttctcatttcatattacccgcattttcccaggcactgagacgcacattcactacaaaaatcattatgaagccgtgttttgctatgaaggtgaaggcgaggtagaaaccctggccgatggcaaaatctggccgatcaaagcaggcgatatttacctgttagatcagcatgacgagcatcttttacgtggcaaagaaaaaggtatgaccgttgcgtgtgtcttcacacctgctattactggcaatgaagtacaccaggaagacggctcatacgcagcacccgcgggtgagtaa

*asd* (*Halomonas* TD): GenBank: OV350343.1 (protein id: CAH1044880.1)

atgttgaaagtcggtttcgtgggatggcgtggcatggttggctcggtgctgatgcagcgcatgcaggaagatggtgattttaacggtattgaaccggtattttttaccacctcccaagttggtcagcctggccccgatatcggcgtggacgtgcctccgctgaaagatgcgtttgatattgaagcgttgaaagcactggacgtggtggttacctgtcaaggtggcgattacaccaagccggtttataaagacctgcgtgaggcgggctggaaaggctactggatcgatgcggccagtaccctgcgcatggaagatgaagcgaccattgttctggatccagttaaccgcaaggtgattgatgcgcagctagcgaagggtgctaaaacctttgttggcggtaactgtacggtcagcttgatgctgatggggctgggtggcttgttcgaagctgacatggttgagtggatgacctccatgacgtaccaagcagcttccggttcaggcgccaagcacatgcgcgaactgttgaaccagatgggtcaattgcgcgatagcgttggcgaagagcttaaagatacctctagcgcgattctggatatcgaccgtaaagtgactgccgctatgcgcagtggtgacttccccacggataatttcggtgcgccgctagcgggcagcctgctgccatggatcgataccaagcttgataatggccaaagccgcgaagagtggaaaggtagtgttgagaccaacaagatcttaggtctcgaaaacaacccgatccccatcgatggtctttgcgtgcgcatcggtgctatgcgttctcatagccaagcgttcaccatcaagcttaagcaagacgtgccgcttgacgagatcgaagatcgcatcgctaagcataatgagtgggttcaactgatcccgaacgacaaagacgctactgttgctgggctaacgcctgccgctgcgacaggtacgctgcaagttccggtcggtcgcttacgcaagcttaatatgggcggcgagtatctgtctgcattcagtgttggtgaccagttgttgtggggtgcggccgaaccgctcaagcgtatgctgaaaattctacgcgagcagtaa

*lysC* (*Corynebacterium glutamicum* 13032): GenBank: BA000036.3 (protein id: BAB97644.1)

atggccctggtcgtacagaaatatggcggttcctcgcttgagagtgcggaacgcattagaaacgtcgctgaacggatcgttgccaccaagaaggctggaaatgatgtcgtggttgtctgctccgcaatgggagacaccacggatgaacttctagaacttgcagcggcagtgaatcccgttccgccagctcgtgaaatggatatgctcctgactgctggtgagcgtatttctaacgctctcgtcgccatggctattgagtcccttggcgcagaagcccaatctttcacgggctctcaggctggtgtgctcaccaccgagcgccacggaaacgcacgcattgttgatgtcactccaggtcgtgtgcgtgaagcactcgatgagggcaagatctgcattgttgctggtttccagggtgttaataaagaaacccgcgatgtcaccacgttgggtcgtggtggttctgacaccactgcagttgcgttggcagctgctttgaacgctgatgtgtgtgagatttactcggacgttgacggtgtgtataccgctgacccgcgcatcgttcctaatgcacagaagctggaaaagctcagcttcgaagaaatgctggaacttgctgctgttggctccaagattttggtgctgcgcagtgttgaatacgctcgtgcattcaatgtgccacttcgcgtacgctcgtcttatagtaatgatcccggcactttgattgccggctctatggaggatattcctgtggaagaagcagtccttaccggtgtcgcaaccgacaagtccgaagccaaagtaaccgttctgggtatttccgataagccaggcgaggctgcgaaggttttccgtgcgttggctgatgcagaaatcaacattgacatggttctgcagaacgtctcttctgtagaagacggcaccaccgacatcaccttcacctgccctcgttccgacggccgccgcgcgatggagatcttgaagaagcttcaggttcagggcaactggaccaatgtgctttacgacgaccaggtcggcaaagtctccctcgtgggtgctggcatgaagtctcacccaggtgttaccgcagagttcatggaagctctgcgcgatgtcaacgtgaacatcgaattgatttccacctctgagattcgtatttccgtgctgatccgtgaagatgatctggatgctgctgcacgtgcattgcatgagcagttccagctgggcggcgaagacgaagccgtcgtttatgcaggcaccggacgctaa

*aceE* (*Halomonas* TD) GenBank: OV350343.1 (protein id: CAH1045134.1)

atgtcggctttaaccacttcttccgcgcgcccaagggtgattttgaaggcgacctgatctacattcagggccacgttgccccggggatttacgcgcgttcttatctggaaggccgtctgtctgaagagcagatggacaaattccgtcgcgaagtcgacggcgatggtctgtcttcctacccgcacccttggctgatgccggattactggcagttccccacggtgtcgatgggtcttggcccgattcaggccatctatcaagcccacgtgatgaagtacctgcatcaccgtgagctgaaagacatgtacgaccgcaagatctggtgctttatgggcgacggcgagtgtgatgagccggagtcactgggcgcgatttctctggcgggtcgtgaaaacctcgataacctgatcttcgtcatcaactgcaacctgcagcgcttggacggtccggtacgcggcaactcccgcgtcatggacgagttcgaaggcgtgttccgcggtgctggctggaacgtcatcaaggtcgtctgggggcgtcactgggatccgctgttcgagaaggacaagaaaggcatcctgcaaaaacgcatggatgaagcagtcgacggtgagtaccagaactacaaggccaacggcggttcgtacacccgtgagcacttcttcggtaagtaccccgaaaccgaagcgatggtcaatgacctgtctgatgaagacatctggaagctcaaccgcggtggtcacgacccgttcaaggtctacgcggcctaccatgaagcggtcaaccagaccaacggtaagcccacggtcatcctggcgcacaccgttaaaggctacggcatgggcagcggcgatggcgaagccgccaacgaagcccaccaggtcaagagcatggagtacgaagcgctgcgcaaattccgcgaccgctttggtattccgatcaccgacgaacagctcaaagacgtgccctactacaagccggaagaagactctcccgagcttaagtacatgcacctgcagcgggaacgcttgaacggctacctgccggcccgccgcagtgactttgaggcgctggagatccccagcctggacgacaaaacctttgcctcgcaaatggttggttccaaagggcgcgaagtctcgaccaccatggcattcgtgcgcgtcctgaacggtctggtcaaggataagaagctcggcaaacatgtcgtaccgattattcctgacgaagcgcgtaccttcggcatggaaggcatgttccgtcagctcggcatctacacctcggaaggtcagaaatatgagccggtcgataaaggccagatcatgttctaccgcgaggatcagaaaggccagattctcgaagaagggattagtgaggctggcgcgatgtctgcgtggattgccgccgcgacgtcctacagcaacaacaacgttaccctgctgccgttctatgtctactactcgatgttcggcttccagcgcattggtgacttggcctgggccgcaggtgacctgcaagcccgcggctttatggtcggcggcactgccgggcgtaccacgctcaacggtgaaggtctgcagcaccaggatggtcacagcttgattcaagcctccaccatacccaactgccgcagctacgacccgacctacgcccacgaagtggcggtcatcctccaggatggtttgaagcgcatgttctccgacaaagagaactgcttctactacctgacggtgatgaacgaaaactacgagcacccagcaatcgacaacgtgcccaccgacgatatcgtcaaaggcatgtacctgctcaacgaaacgaaaggcgacaagggccgcgtgcaactgatgggctccggtaccatcctgcgcgaagtcgaagccgccgcagagctgttggccaacgactggggcattggcgcggatatctggagcgtgaccagctttaacgagctgcgtcgcgaagcgctgctgttggagcgtgaagccttcttgaacccggacgttgagggcaacaagccccacgtgacgaagtgcctggaaggccgtgacggcccggtgattgcctccaccgactacatgaagctctacgccgaccaagtgcgtgcctgggtgccaagcgagtacaccgtgctgggtacagacggctttggccgttccgacacccgcgagaagctgcgctacttctttgaagtagaccgctacttcgtcaccgtggcggcgctacgtgcgctggcggaccgcggtgagcttgatcgcaagcatgtcggcgaggcgctgaagaagtatggcatcgacgccaacaagccgaacccgctgaccagctaa

*RppA* (*Streptomyces griseus*) GenBank: AB218878.1 (protein id: BAE07216.1)

atggcgaccctgtgccgaccggccatcgctgtgcccgagcacgtcatcacgatgcagcagaccctggacctggcccgggagacccatgccgggcacccgcagcgcgacctcgtcctgaggctcatccagaacaccggcgtccagacccggcacctcgtgcagcccatcgagaagaccctggcgcaccccggattcgaggtgcgcaaccaggtgtacgaggccgaggccaagacccgggtccccgaagtcgtccggcgggcgctcgccaacgccgagaccgagccgtccgagatcgacctgatcgtctacgtctcctgcacgggtttcatgatgccctcgctgaccgcgtggatcatcaacagcatgggcttccggcccgagacccgccaactgcccatcgcccagctcggctgtgcggcgggcggcgcggcgatcaaccgcgcgcacgacttctgcgtggcctaccccgactccaacgtcctcatcgtgtcctgcgagttctgctcgctgtgctaccagcccaccgacatcggggtcggttccctgctctccaacggactcttcggcgacgcgctctccgcggccgtcgtacggggacagggcggcaccggcatgcgcctggagcgcaacggctcccacctggtgcccgacaccgaggactggatctcctacgcggtccgcgacaccgggttccacttccagctggacaagcgggtcccgggcaccatggagatgctcgccccggtgctcctggacctggtcgacctgcacggctggtccgtcccgaacatggacttcttcatcgtccacgcgggcggaccgcgcatcctggacgacctctgccacttcctcgacctgccgcccgagatgttccgctacagccgggccaccctcaccgaacgcggcaacatcgcgagctccgtcgtcttcgacgcgctggcgcgcctcttcgacgacggcggcgccgccgagtccgcgcaggggctcatcgccggcttcggtcccggcatcaccgccgaggtggccgtggggagttgggccaaggaaggcctcggggcggacgtcggacgcgacctcgacgagctggagctgaccgccggcgttgcgctgtccggctga

*NphT7* (*Streptomyces sp.*) GenBank: AB540131.1 (protein id: D7URV0.1)

Atgaccgacgtccgcttccgcatcatcggcaccggcgcctacgtgccggagcgcatcgtcagcaacgacgaggtgggcgcccccgccggcgtcgacgatgactggatcacccgcaagaccggcatccgccagcgccgctgggcggccgatgaccaggccacctccgacctggcgaccgccgccggccgcgccgccctgaaggccgccggcatcacccccgagcagctgaccgtgatcgccgtcgccacctccacccccgaccgcccccagccgccgaccgccgcctacgtccagcaccatctgggcgccaccggcaccgccgcgttcgacgtgaacgccgtctgcagcggcaccgtgttcgcgctgagctcggtggccggcaccctggtgtaccgcggcggctacgccctcgtgatcggcgccgacctgtactcccgcatcctgaatccggccgaccgcaagaccgtcgtgctgttcggcgacggcgccggcgccatggtcctgggcccgacctcgaccggcaccggcccgatcgtgcgccgcgtcgccctccataccttcggcggcctgaccgacctgatccgcgtcccggccggcggcagccgccagccgctggacaccgacggcctcgacgccggcctgcagtacttcgccatggacggccgcgaggtgcgccgcttcgtgaccgaacacctgccgcagctgatcaagggcttcctgcacgaggccggcgtcgacgccgccgacatcagccacttcgtcccgcaccaggccaacggcgtgatgctggacgaggtgttcggcgagctgcatctgccgcgcgccacgatgcaccgcaccgtggaaacctacggcaacaccggcgccgccagcatccccatcaccatggacgccgccgtccgcgccggcagcttccgccccggcgagctggtgctgctggccggcttcggcggcggcatggccgcctccttcgccctgatcgagtggtga

*Ftl* (Methylobacterium extorquens AM1) GenBank: CP001510.1 (protein id: ACS38281.1)

atgccgtctgacatcgagatcgctcgtgctgctaccctgaagccgatcgctcaggttgctgagaagctgggtatcccggacgaagctctgcacaactacggtaaacacatcgctaaaatcgaccacgacttcatcgcttctctggaaggtaaaccggaaggtaaactggttctggttaccgctatctctccgaccccggctggtgaaggtaaaaccaccaccaccgttggtctgggtgacgctctgaaccgtatcggtaaacgtgctgttatgtgcctgcgtgaaccgtctctgggtccgtgctttggtatgaagggtggtgctgctggtggtggtaaagctcaggttgttccgatggaacagatcaacctgcacttcaccggtgacttccacgctatcacctctgctcactctctggctgctgctctgatcgacaaccacatctactgggctaacgaactgaacatcgacgtgcgtcgtatccactggcgtcgtgttgttgacatgaacgaccgtgctctgcgtgctatcaaccagtctctgggtggtgttgctaacggtttcccgcgtgaagacggttttgacatcaccgttgcttctgaggttatggctgtgttctgcctggccaaaaacctggctgacctggaagaacgtctgggtcgtatcgttatcgctgagacccgtgaccgtaaaccggttaccctggctgacgttaaagctaccggtgctatgaccgttctgctgaaggacgctctgcaaccgaacctggttcagaccctggaaggtaacccggctctgatccacggtggtccgtttgctaacatcgctcacggttgcaactctgttatcgctacccgtaccggtctgcgtctggctgactacaccgttaccgaagctggttttggtgctgacctgggtgctgagaagttcatcgacatcaaatgccgtcaaactggtctgaagccgtctgctgttgttatcgttgctaccatccgtgctctgaagatgcacggtggtgttaacaaaaaagacctgcaagctgagaacctggacgctctggagaagggttttgctaacctggaacgtcacgttaacaacgtgcgttcttttggtctgccggttgttgttggtgttaaccacttcttccaggacaccgacgctgaacacgctcgtctgaaggaactgtgccgtgaccgtctgcaagttgaagctatcacctgcaaacactgggctgaaggtggtgctggtgctgaagctctggctcaggctgttgttaaactggctgaaggtgaacagaagccgctgacctttgcttacgagaccgagaccaaaatcaccgacaaaatcaaagctatcgctaccaaactgtacggtgctgctgacatccagatcgaatctaaagctgctaccaaactggctggttttgagaaggacggttacggtggcctgccggtctgcatggcaaaaacccagtactctttctctaccgacccgaccctgatgggtgctccgtctggccacctggtgagcgtgcgtgacgtgcgtctgtctgctggtgctggttttgttgttgttatctgcggtgagatcatgaccatgccgggtctgccgaaggttccggctgctgacaccatccgtctggacgctaacggtcagatcgacggtctgttctaa

*fch* (Methylobacterium extorquens AM1) GenBank: CP001510.1 (protein id: ACS39573.1)

atggctggtaacgaaaccatcgaaaccttcctggacggtctggcttcttctgctccgaccccgggtggtggtggtgctgctgctatctctggtgctatgggtgctgctctggtttctatggtttgcaacctgaccatcggtaaaaaaaaatacgttgaagttgaagctgacctgaaacaggttctggaaaaatctgaaggtctgcgtcgtaccctgaccggtatgatcgctgacgacgttgaagctttcgacgctgttatgggtgcttacggtctgccgaaaaacaccgacgaagaaaaagctgctcgtgctgctaaaatccaggaagctctgaaaaccgctaccgacgttccgctggcttgctgccgtgtttgccgtgaagttatcgacctggctgaaatcgttgctgaaaaaggtaacctgaacgttatctctgacgctggtgttgctgttctgtctgcttacgctggtctgcgttctgctgctctgaacgtttacgttaacgctaaaggtctggacgaccgtgctttcgctgaagaacgtctgaaagaactggaaggtctgctggctgaagctggtgctctgaacgaacgtatctacgaaaccgttaaatctaaagttaactaa

*mtdA* (Methylobacterium extorquens AM1) GenBank: AB540131.1 (protein id: ACS39572.1)

atgtctaaaaaactgctgttccagttcgacaccgacgctaccccgtctgttttcgacgttgttgttggttacgacggtggtgctgaccacatcaccggttacggtaacgttaccccggacaacgttggtgcttacgttgacggtaccatctacacccgtggtggtaaagaaaaacagtctaccgctatcttcgttggtggtggtgacatggctgctggtgaacgtgttttcgaagctgttaaaaaacgtttcttcggtccgttccgtgtttcttgcatgctggactctaacggttctaacaccaccgctgctgctggtgttgctctggttgttaaagctgctggtggttctgttaaaggtaaaaaagctgttgttctggctggtaccggtccggttggtatgcgttctgctgctctgctggctggtgaaggtgctgaagttgttctgtgcggtcgtaaactggacaaagctcaggctgctgctgactctgttaacaaacgtttcaaagttaacgttaccgctgctgaaaccgctgacgacgcttctcgtgctgaagctgttaaaggtgctcacttcgttttcaccgctggtgctatcggtctggaactgctgccgcaggctgcttggcagaacgaatcttctatcgaaatcgttgctgactacaacgctcagccgccgctgggtatcggtggtatcgacgctaccgacaaaggtaaagaatacggtggtaaacgtgctttcggtgctctgggtatcggtggtctgaaactgaaactgcaccgtgcttgcatcgctaaactgttcgaatcttctgaaggtgttttcgacgctgaagaaatctacaaactggctaaagaaatggcttaa

*ftl* (Vibrio natriegens) GenBank: CP016345.1 (protein id: ANQ14326.1)

atgcagtccgacattgaaatttgccgaaacacccctctatcctcaatcgatactattgccgcgaatgcaggtttacagcctgatgaatatgatacgcacggcaaacacaaagccaaagttcatccaagatgtttaaaccgtctcaaagaaaacaaagacggtaaattagtattagtcacggccataacaccaacgccactcggcgaaggtaagacagtcacaactatcggccttgcacaagggttagcaaagttaaaccaatccgtgatggcctgcatcagacagccttcaatgggaccagtatttggaatcaaaggtggtgcggccggcggtggttactctcaagtcgcaccgatggaagagttaaacttacatttaactggcgatattcacgccgttaccgctgctcataaccttgcatcagccgcgctagatgcccgtctataccacgagcaacgtgaaggctacgacgcattcgaagctcgtactggtttaaaagcattaaaaatagatgtcgacagtatcacatggaagcgcgtaatggatcataatgatcgcgcactacgcatggtaaaaattggtctaaacgaaccaggtaaaaccattaatggcatcgaaagaagcgaaggtttcgacatttctgccgcatcagaactcatggcaatcattgcactcgccaaagatttagcagacctgcgtaaacgaatcggacgaattgttgttgcttgtgatttagatggccatccggtgacgactgaagaccttcaagtcgcgggtgctatggctgtaacgttgaaagaagccattgcgcctacactaatgcagactttagaaggtgtccctactcttattcacgctggtccctttgcgaatattgctcacggcaactcttctatcatcgctgatgaaattgcactaaaactttctagttacactgtaactgaagccggttttggatcggatatggggctggagaaagcatgtaatattaaagcagcagcctcaaaccacgctcctgattgtgtggtgattgttgccaccttacgtggcctaaaagcgaattcggggcattatgatcttcgcccgggcactgctattcctgaatcgatatttaaccccgataagccagcgctagaagccggatttgaaaacctgaagtggcatatcaataacgttaagaaatacggccttcctgccgttgtcgcgatcaaccagtttccgcaagactgcgaacaggaactctcagtactgcgtcagatgattcgtgactatgacccaagtgtcaatgtggccatcagtaccgctttctcaaacggtggtgaaggtaccgtagagctcgctcaatttgtggtagaagcatgtaatactcaaacggatttccgcccgctttatacaaaagaacaaaagctggaagagaaattaatgtcggtttgtgaagctgggtatggcgcgtcaaatgtcgaattgagtgatctgagcatttcacagcttaagaggtttgaagctttaggttttaatgacctcgcagtgtgtattgcgaaaacgccactttctatcactaccgactccagcgtcaaaggggctccgcgtggatttacggttccaatacgtgaattacgtttatgcgctggcgcagggttcatttatgcactatcaggcagtgtgatgacgatgccagggcttccggacaaacctgcttttatgaacttggatttagatgaggaaggcaacattattggtttgtcataa

*folD* (Vibrio natriegens) GenBank: CP016345.1 (protein id: ANQ12003.1)

Atgactgctcaaaatattgatgggactctcatttcccaaactgttcgttctgaagttgcagcacgagtaaaagctcgcgttgaagctggattacgtgcccctggccttgcagttgttctagttggtgaagaccctgcttctcaagtttacgttggcagtaaacgccgcgcttgtgaagaagtgggttttgtttccaagtcttttgaccttccggcatcaaccactgaagcagaattgctagcgttgatcgatgagttgaacaatgacaacgaaattgatggcattttagttcagcttccacttcctgctggaattgatagcactcatgttttagaacgtattcatccagagaaagacgtggatggcttccacccttataacgtaggtcgtcttgctcagcgtatccctaaactacgctcttgtacgccgaaaggtattattactctgctcgaccgctacaacattaatcttcgtggtaagcatgctgtggttgttggtgcttcaaatatcgttggtcgtccaatgacacttgagttgctattggcgggctgcacaacgacgacctgtcaccgctttactaaagacttagagagctacgttcgtcaagccgacgtcgttgttgtcgcagtaggtaaaccaaacttcattcctggcgaatggattaagaaaggcgctgtggttgtcgatgtgggtatcaaccgtcttgactctggtaagctggttggcgacgtggaatacgataaagcacgtgaaagcgcgagcttcattactccagtacctggtggcgttggtccaatgacagtagcaagcctgattgaaaacacaatgctggcttgcgagcagttccatacggacaagtaa

*vioABCDE* (*Chromobacterium violaceum*)

atgaagcactcttctgacatctgtatcgttggtgctggtatctctggtttgacctgtgcttctcacttgttggactctccagcttgtagaggtttgtctttgagaatcttcgacatgcaacaagaagctggtggtagaatccgatctaagatgttggacggtaaggcttctatcgaattgggtgctggtagatactctccacaattgcacccacacttccaatctgctatgcaacactactctcaaaagtctgaagtttacccattcacccaattgaagttcaagtctcacgttcaacaaaagttgaagagagctatgaacgaattgtctccaagattgaaggaacacggtaaggaatctttcttgcaattcgttagtagataccaaggtcacgactctgctgttggtatgatccgatctatgggttacgacgctttgttcttgccagacatctctgctgaaatggcttacgacatcgttggtaagcacccagaaatccaatctgttaccgacaacgacgctaaccaatggttcgctgctgaaacaggtttcgctggtttgatccaaggtatcaaggctaaggttaaggctgctggtgctagattctctttgggttacagattgttgtctgttagaaccgacggtgacggttacttgttgcaattggctggtgacgacggttggaagttggaacacagaaccagacacttgatcttggctatcccaccatctgctatggctggtttgaacgttgacttcccagaggcttggtctggtgctagatacggttctttgccattgttcaagggtttcttgacctacggtgaaccatggtggttggactacaagttggacgaccaagttttgatcgttgacaacccattgagaaaaatctacttcaagggtgacaagtacttgttcttctacaccgactctgaaatggctaactactggagaggttgtgttgctgaaggtgaagacggttacttggaacaaatcagaacccacttggcttctgctttgggtatcgttagagaaagaatcccacaaccattggctcacgttcacaagtactgggctcacggtgttgagttctgtagagactctgacatcgaccacccatctgctttgtctcacagagactctggtatcatcgcttgttctgacgcttacaccgaacactgtggttggatggaaggtggtttgttgtctgctagagaggctagtagattgttgttgcaaagaatcgctgctttgagaagataggccttactagagaaagaggagaaatactagatgagcattctggatttcccgcgtatccacttccgtggctgggcccgtgtcaatgcgccgaccgcgaaccgcgatccgcacggccacatcgatatggccagcaataccgtggcgatggcgggtgagccgttcgacctggcacgccatcctacggagttccaccgtcacctgcgctccctgggtccgcgcttcggcttggatggtcgtgctgacccggaaggcccgttcagcctggccgagggctacaacgctgccggtaacaaccacttttcgtgggagagcgcaaccgttagccacgtgcaatgggatggcggtgaggcggatcgtggtgacggtctggtcggtgctcgtttggcactgtggggtcactacaatgattatctgcgtaccaccttcaatcgtgctcgttgggtcgacagcgacccgacgcgccgtgacgctgcacaaatctatgcgggccaattcaccattagcccggctggtgccggtccgggtacgccgtggctgtttacggcagacattgatgatagccatggtgcacgttggacgcgtggcggccacattgcagagcgtggcggccacttcttggatgaagagtttggtctggcacgcctgtttcagttctctgtgccgaaagatcacccacattttctgtttcacccgggtccgtttgattccgaggcctggcgtcgtctgcaattggctctggaggatgacgacgttctgggtctgaccgtgcaatatgcgttgttcaatatgagcaccccgcctcagccgaacagcccggtttttcacgatatggtcggtgttgtcggtctgtggcgtcgtggtgaactggcgagctacccggctggtcgtctgctgcgtccgcgtcaaccgggtctgggtgacctgaccctgcgcgtcaacggtggtcgcgttgcgctgaatttggcgtgtgccattccgttcagcactcgtgccgcgcagccaagcgcaccggaccgcctgaccccggacctgggtgccaaactgccgctgggcgatctgctgctgcgtgatgaggacggcgcactgttggcacgtgtgccgcaggctctgtaccaagactattggacgaatcacggtattgtggacctgccgctgctgcgcgaaccgcgtggtagcttgaccctgagcagcgaactggcggagtggcgtgagcaagactgggtcacccaaagcgacgcgtctaacctgtacctggaggcaccggatcgccgtcacggtcgctttttccctgagagcatcgcgctgcgcagctactttcgcggtgaagcgcgtgcgcgtccggatatcccgcatcgtatcgagggcatgggcctggtcggcgtcgaatctcgtcaggatggcgacgctgcggaatggcgtctgacgggtctgcgtccgggtccggcacgcattgttctggacgatggtgccgaggcgatccctctgcgtgttctgcctgacgattgggcgctggatgacgcgaccgtcgaagaagtggattacgcctttttgtaccgccacgttatggcgtattacgagctggtgtatccattcatgagcgacaaggtgttttccctggctgatcgttgcaaatgtgaaacgtacgcacgtctgatgtggcagatgtgtgatccgcagaaccgcaacaagtcctattacatgccgagcacccgcgaactgtcggcaccgaaagctcgtttgttcttgaagtatctggcccacgtggaaggccaggcacgcctgcaagcacctccgccagcgggtccggcacgcattgaatctaaagcccagttggcggcagagctgcgtaaagccgtcgacctggagctgtctgtgatgctgcaatacctgtacgcggcgtatagcattccgaactatgcacagggccaacaacgtgttcgtgacggtgcgtggaccgccgagcagctgcaactggcgtgcggtagcggtgaccgtcgccgtgatggcggtattcgtgcagcactgctggaaattgctcatgaagaaatgattcattacctggtcgttaacaacctgctgatggccctgggcgagccgttctacgcgggtgtcccgctgatgggcgaagcggcacgtcaggcgtttggcctggacaccgagttcgctctggaaccgtttagcgaaagcacgctggcacgttttgttcgtctggaatggccgcactttatcccagcaccgggcaaatccatcgcggactgctatgccgccattcgtcaggcgtttttggatctgccggacttgtttggtggcgaggcaggtaagcgtggcggtgaacaccacctgttcctgaatgagctgaccaaccgtgcgcatccgggttatcaactggaagttttcgatcgcgactcggcgctgtttggtattgcatttgtgaccgatcagggcgaaggtggcgctctggacagcccgcactacgaacatagccattttcaacgtctgcgtgaaatgagcgcgcgtatcatggctcaaagcgcaccgttcgaaccggcgctgccggcgttgcgtaatccggttctggatgagagcccgggttgccaacgtgtcgcagacggtcgtgcgcgtgcgctgatggcattgtaccaaggcgtttatgagctgatgtttgcgatgatggcgcagcacttcgccgtgaaaccgctgggtagcttgcgtcgcagccgcctgatgaacgcagcaatcgatctgatgaccggtctgttgcgtccgctgagctgcgcgctgatgaacctgccaagcggcatcgccggtcgcacggccggtccgccgctgccgggtccggttgacacccgtagctatgacgactacgcgctgggctgtcgcatgctggcacgccgttgcgagcgtctgctggagcaggcgagcatgctggaaccgggttggctgccggatgcgcagatggagctgctggatttctatcgtcgccaaatgctggacttggcgtgcggcaaactgagccgcgaggcctaatagccactactagagaaagaggagaaatactagatgaaaagagctatcatcgttggtggtggtttggctggtggtttgaccgctatctacttggctaagagaggttacgaagttcacgttgttgaaaagagaggtgacccattgagagacttgtcttcttacgttgacgttgtttctagtagagctatcggtgtttctatgaccgttagaggtatcaagtctgttttggctgctggtatcccaagagctgaattggacgcttgtggtgaaccaatcgttgctatggctttctctgttggtggtcaatacagaatgagagaattgaagccattggaagacttcagaccattgtctttgaacagagctgctttccaaaagttgttgaacaagtacgctaacttggctggtgttagatactacttcgaacacaagtgtttggacgttgacttggacggtaagtctgttttgatccaaggtaaggacggtcaaccacaaagattgcaaggtgacatgatcatcggtgctgacggtgctcactctgctgttagacaagctatgcaatctggtttgagaagattcgagttccaacaaaccttcttcagacacggttacaagaccttggttttgccagacgctcaggctttgggttacagaaaggacaccttgtacttcttcggtatggactctggtggtttgttcgctggtagagctgctaccatcccagacggttctgtttctatcgctgtttgtttgccatactctggttctccatctttgaccaccaccgacgaaccaaccatgagagctttcttcgacagatacttcggtggtttgccacgcgacgctcgcgacgaaatgttgagacaattcttggctaagccatctaacgacttgatcaacgttcgatcttctaccttccactacaagggtaacgttttgttgttgggtgacgctgctcacgctaccgctccattcttgggtcaaggtatgaacatggctttggaagacgctagaaccttcgttgaattgttggacagacaccaaggtgaccaagacaaggctttcccagagttcaccgaattgagaaaggttcaagctgacgctatgcaagacatggctagagctaactacgacgttttgtcttgttctaacccaatcttcttcatgagagctagatacaccagatacatgcactctaagttcccaggtttgtacccaccagacatggctgaaaagttgtacttcacctctgaaccatacgacagattgcaacaaatccaaagaaagcaaaacgtttggtacaagatcggtagagttaactagaccgtactagagaaagaggagaaatactagatgaaaatcttggttatcggtgctggtccagctggtttggttttcgcttctcaattgaagcaagctagaccattgtgggctatcgacatcgttgaaaagaacgacgaacaagaagttttgggttggggtgttgttttgccaggtagaccaggtcaacacccagctaacccattgtcttacttggacgctccagaaagattgaacccacaattcttggaagacttcaagttggttcaccacaacgaaccatctttgatgtctaccggtgttttgttgtgtggtgttgaaagaagaggtttggttcacgctttgagagacaagtgtcgatctcaaggtatcgctatcagattcgaatctccattgttggaacacggtgaattgccattggctgactacgacttggttgttttggctaacggtgttaaccacaagaccgctcacttcaccgaggctttggttccacaagttgactacggtagaaacaagtacatctggtacggaacctctcaattgttcgaccaaatgaacttggttttcagaacccacggtaaggacatcttcatcgctcacgcttacaagtactctgacaccatgtctaccttcatcgttgaatgttctgaagaaacctacgctagagctagattgggtgaaatgtctgaagaggcttctgctgaatacgttgctaaggttttccaagctgaattgggtggtcacggtttggtttctcaaccaggtttgggttggagaaacttcatgaccttgtctcacgacagatgtcacgacggtaagttggttttgttgggtgacgctttgcaatctggtcacttctctatcggtcacggaaccacaatggctgttgttgttgctcaattgttggttaaggctttgtgtaccgaagacggtgttccagctgctttgaagagattcgaagaaagagctttgccattggttcaattgttcagaggtcacgctgacaacagtagagtttggttcgaaaccgttgaagaaagaatgcacttgtcttctgctgagttcgttcaatctttcgacgctagaagaaagtctttgccaccaatgccagaggctttggctcaaaacttgagatacgctttgcaaagatagctcgtactagagaaagaggagaaatactagatggaaaacagagaaccaccattgttgccagctagatggtcttctgcttacgtttcttactggagtccaatgttgccagacgaccaattgacctctggttactgttggttcgactacgaaagagacatctgtagaatcgacggtttgttcaacccgtggtctgaaagagacaccggttacagattgtggatgtctgaagttggtaacgctgcttctggtagaacctggaagcaaaaggttgcttacggtagagaaagaaccgctttgggtgaacaattgtgtgaaagaccattggacgacgaaaccggtccattcgctgaattgttcttgccacgcgacgttttgagaagattgggtgctagacacatcggtagaagagttgttttgggtagagaagctgacggttggagataccaaagaccaggtaagggtccatctaccttgtacttggacgctgcttctggtaccccattgagaatggttaccggtgacgaggctagtagagcttctttgagagacttcccaaacgtttctgaagctgaaatcccagacgctgttttcgctgctaagagatag

*sod* (*Escherichia coli*): GenBank: CP062832 (protein id: QPE32704.1)

atgtcattcgaattacctgcactaccatatgctaaagatgctctggcaccgcacatttctgcggaaaccatcgagtatcactacggcaagcaccatcagacttatgtcactaacctgaacaacctgattaaaggtaccgcgtttgaaggtaaatcactggaagagattattcgcagctctgaaggtggcgtattcaacaacgcagctcaggtctggaaccatactttctactggaactgcctggcaccgaacgccggtggcgaaccgactggaaaagtcgctgaagctatcgccgcatcttttggcagctttgccgatttcaaagcgcagtttactgatgcagcgatcaaaaactttggttctggctggacctggctggtgaaaaacagcgatggcaaactggctatcgtttcaacctctaacgcgggtactccgctgaccaccgatgcgactccgctgctgaccgttgatgtctgggaacacgcttattacatcgactatcgcaatgcacgtcctggctatctggagcacttctgggcgctggtgaactgggaattcgtagcgaaaaatctcgctgcataa
